# Supplementary material for: A phase I/II study of adoptive immunotherapy using donor liver graft-derived NK cell-enriched immune cells to prevent severe infection after liver transplantation
Source: PLoS One. 2025 Jan 16;20(1):e0313102. doi: 10.1371/journal.pone.0313102 (PMC11737672; doi:10.1371/journal.pone.0313102)
Supplement: S1 File — (PDF) [file pone.0313102.s001.pdf]

Clinical Research Implementation Plan

Adoptive immunotherapy using donor liver graft derived natural killer (NK) cells for preventing severe infection after liver transplantation

Principal Investigator Hideki Ohdan

Affiliation / Position Professor, Department of Gastrointestinal and Transplantation Surgery, Hiroshima University Graduate School of Medical Sciences

Created on May 20, 2019, 8th edition.

Approval date	
Signature of approver	

Enactment and revision history table					
version number	Date of enactment or revision	reason	approval	confirmation	creation
1	2015.8.3	Newly enacted			
2	2015.9.14	Revision by committee opinion			
3	2015. 11.20	Revision by committee opinion			
4	2016.2.16	Revision due to change of project leader			
5	2017.3.13	Revision due to change of project leader			
6	2018.10.1	Revision due to extension of research period			
7	2018.10.26	Revision by committee opinion			
8	2019.5.20	Revision due to changes in the Ministerial Ordinance for Regenerative Medicine and IL-2 formulation			

contents

0 Overview 8
Schema 13
Observation and Inspection Schedule 14
Word definitions 16
Background (plain language) 17
Content (plain language): 1. 17
Objective 1 ....Research Objectives 18
2 Longitude and latitude 18
2.1 Target disease 18
2.1.1 Concepts, definitions, etiology, and pathogenesis 18
2.1.2 Epidemiology 18
2.1.3 Standard Treatment and Prognosis 19
2.1.4 Rationale for setting target diseases 19
2.2 Name of the specified cell processing product and its summary 19
2.2.1 2.2.1 Name of specified cell processing product 19
2.2.2 Overview of the test object 19
2.2.3 Summary of results of preclinical studies, clinical studies, and clinical trials to date 20
2.2.4 Reasons for judging that it is possible to conduct clinical research 24
2.3 Anticipated benefits and disadvantages for enrolled patients 25
2.3.1 Projected profit 25
2.3.2 Anticipated disadvantages 25
2.4 Significance of this clinical study 26
3 Target Diseases and Eligibility Criteria 26
3.1 Target disease 26
3.2 Selection criteria 26
3.3 Exclusion Criteria 27
4 Consent obtained 28
4.1 Preparation of consent explanatory documents and consent forms 28
4.2 Revision of consent explanatory document and consent form 29
4.3 Time and method of explaining consent and obtaining consent 29
5 Registration 29

5.1 Subjects enrolled 29
6 Specified cell processing products used in research 30
6.1 Name of specified cell processing products 30
6.2 Composition, structure, properties, and manufacturing method 30
6.2.1 Standard 30
6.2.2 Manufacturing method 31
6.3 Container, packaging and storage conditions 32
6.4 Delivery/transportation 32
6.5 Management and storage 32
7 Test Design 33
7.1 Study Design 33
7.2 Target number of enrolled subjects and enrollment period 33
7.3 Possibility of accumulating the target number of enrolled subjects 34
7.4 Protocol treatment plan 34
7.4.1 Definition of Protocol Treatment 34
7.4.2 Method 35
7.4.3 Combination therapy 35
7.4.4 Infectious disease treatment 35
7.4.5 Rationale for establishing a protocol treatment plan 35
7.4.6 Duration of study participation of enrolled subjects 35
7.5 Interim total 36
Primary and secondary endpoints ..... Primary and secondary endpoints 36
8.1 Primary endpoint 36
8.2 Secondary endpoints 36
9 Observation and Inspection Items and Schedule 37
9.1 Inspection Schedule 37
9.2 Observation and examination items 39
9.2.1 Screening test 39
9.2.2 Preoperative examination 40
9.2.3 Registration 41
9.2.4 Date of surgery 41
9.2.5 Day of NK cell administration (postoperative day 3) 41
9.2.6 1, 2 days after NK cell administration (postoperative days 4, 5) 42
9.2.7 1, 2 weeks after NK cell administration 43
9.2.8 4 weeks after NK cell administration 43

9.2.9 After 8 weeks and 12 weeks of NK cell administration 44
9.2.10 6 months after NK cell administration, and every 6 months thereafter until 3 years later........ 44
9.2.11 When aborted 45
10 Ensuring the safety of subjects........ 45
10.1 Basic matters 45
10.2 Definition of adverse events........ 45
10.3 Assessment of adverse events 46
10.4 Anticipated adverse events and response........ 46
10.4.1 Predicted adverse events 46
10.4.2 Dealing with adverse events........ 46
10.5 Response to outbreaks of disease, etc........ 47
10.5.1 Measures to be taken in case of outbreak of disease, etc 47
10.5.2 Report of diseases, etc. to the Accreditation Committee for Regenerative Medicine, etc........ 48
10.5.3 Report of illness, etc. to the Minister of Health, Labour and Welfare 49
11. Criteria and procedures for discontinuation of clinical research for each subject........ 50
11.1 Criteria for discontinuation of clinical research for each subject........ 50
11.2 Procedures for discontinuing clinical research for each subject........ 51
12 Compliance with, deviation from, or modification of clinical research protocol 51
12.1 Compliance with the clinical research protocol........ 51
12.2 Deviation or change from the implementation plan........ 51
13 Termination or discontinuation of the entire clinical study........ 52
13.1 Completion of clinical research........ 52
13.1.1 Procedures for terminating research in clinical studies 52
13.2 Criteria and procedures for interruption or discontinuation of the entire clinical study........ 52
13.2.1 Criteria for interruption or discontinuation of clinical research as a whole 52
13.2.2 Procedures for interruption or discontinuation of the entire clinical study 53
14 Case report........ 53
14.1 Data Management 53

14.2 Case report writing and data collection 53
14.3 Notes on the case report form 53
14.4 Change or modification of case report form 54
14.5 Review of case report forms 54
14.6 Submission of case report form 54
15 Statistical analysis 55
15.1 Statistical analysis method 55
15.2 Definition of the population for analysis 55
15.2.1 Efficacy analysis population 55
15.2.2 Safety analysis population 55
15.3 Handling of missing values 55
15.4 Subject background and baseline characteristics 56
15.5 Efficacy analysis 56
15.5.1 Primary endpoint 56
15.5.2 Secondary endpoints 56
15.6 Safety analysis 57
15.7 Procedure for changing the analysis plan 58
16 Quality control for clinical research 58
16.1 Quality control 58
16.2 Monitoring 58
16.3 Data management 59
16.4 Effectiveness and Safety Evaluation Committee 59
16.4.1 Content of deliberations by the Efficacy and Safety Evaluation Committee 59
16.4.2 Recommendations by the Effectiveness and Safety Assessment Committee 59
16.4.3 Audit 60
16.5 Education and training of researchers 60
17 Committee for Regenerative Medicine, Hiroshima University 60
17.1 Deliberation by the Hiroshima University Committee for Regenerative Medicine 60
17.2 Deliberations of the Hiroshima University Committee for Regenerative Medicine 61
17.3 Recommendation by the Hiroshima University Committee on Frontier Medical Sciences 61
18 Ethical Conduct of Clinical Research 61

18.1 Hiroshima University Committee for Regenerative Medicine ... 61
18.2 Progress report on clinical research ... 62
18.3 Matters related to the protection of human rights and personal information of subjects ... 62
18.3.1 Human rights of subjects ... 62
18.3.2 Protection of personal information ... 62
Records, etc ... Preservation of records, etc. 63
19.1 Preservation of samples ... 63
19.2 Preservation of materials ... 63
20 Preparation of clinical research summary report ... 64
21 Understanding of subject information after completion of clinical research . 64
22 Compensation for clinical research expenses and health damage ... 65
22.1 Sources of funding for clinical research and conflicts of interest ... 65
22.2 Cost burden related to clinical research ... 65
22.3 Compensation for health damage, etc ... 65
23 Arrangements for attribution of clinical research results and registration and publication of clinical research results ... 66
Clinical research implementation system ... 66
23.1 Principal Investigator ... 66
23.2 Research subcontractor ... 66
23.3 Contact ... 68
24 literature ... 68

0 Overview

Item	contents
Subject name	Clinical application of postoperative immunostimulatory therapy using donor liver-derived activated natural killer (NK) cells in liver transplantation for the prevention of infectious diseases
Purpose of the test	In an open-label study, we will investigate whether immunostimulatory therapy using activated natural killer (NK) cells derived from liver donors on day 3 after living donor liver transplantation reduces the incidence of bacteremia during the first month after liver transplantation compared with historical data in patients with uncompensated cirrhosis who have no treatment other than liver transplantation. The results of the study were as follows
Test Design	Single-center, open-label, historical data controlled study, Hiroshima University Hospital
Specific Cell Processing Products	Activated natural killer (NK) cells from liver transplant donor livers
selection criteria	Selection criteria for recipients (those who receive regenerative medicine)<br>1) Patients undergoing living partial liver transplantation for the treatment of non-compensated cirrhosis refractory to medical therapy<br>2) Patients aged 20 years or older at the time of obtaining consent<br>3) Patients for whom written consent has been obtained from the patient or a substitute.<br><br>Selection criteria for cell donors<br>1) Those who meet the criteria of the Liver Transplant Study Group's "Guidelines for Living Donor Surgery" and undergo living donor surgery as liver donors.<br>2) Those who are at least 20 years old at the time of obtaining consent<br>3) A person who has given written consent to the preparation of liver-derived natural killer (NK) cells from the perfusate of a donor liver graft and their administration to the recipient.
exclusion criteria	Recipient exclusion criteria

	1) Patients undergoing re-liver transplantation<br>2) Patients undergoing brain-dead liver transplantation<br>3) Other patients whose participation in this clinical research is deemed inappropriate by the principal investigator or sub-investigator.<br><br>Exclusion criteria for cell donors (donors)<br>1) Persons undergoing living donor liver transplant surgery for re-liver transplantation<br>2) A person who is judged by the principal investigator or sub-investigator to be inappropriate to participate in this clinical research.
Subjects' consent	Before the liver transplantation procedure, an explanation of consent will be given and consent will be obtained from the subject (donor or recipient) or a substitute.<br>The principal investigator or sub-investigator shall provide and use the consent explanatory document to the subject himself/herself or his/her substitute who is a candidate for participation in this clinical research, and obtain his/her written consent for participation in this clinical research after providing sufficient oral explanation.
Evaluation Items	Primary endpoints<br>Incidence of bacteremia during the first month after surgery<br>Secondary endpoints<br>① Overall survival (6 months, 1, and 3 years postoperatively)<br>② Effect on immune response<br>Evaluation of donor-specific immune responsiveness by CFSE-MLR (1, 2, 3, and 4 weeks postoperatively)<br>Detection of DSA (donor specific antibody) (annual screening)<br>Incidence of rejection<br>Evaluation of NK cell activity in peripheral blood of recipients<br>③ Presence of hepatocellular carcinoma recurrence (hepatocellular carcinoma cases only)<br>Presence and timing of de novo carcinogenesis<br>④ Incidence of infectious diseases (bacterial infections, cytomegalovirus infections, fungal infections, analysis by

	genetic polymorphism)<br>⑤ Safety assessment (type and severity of adverse events, frequency of occurrence, duration of occurrence, causal relationship)
Research Methods<br>(Treatment)<br>(Schedule)	1 . Cell harvesting<br>In the operating room of Hiroshima University Hospital, the perfusate of the donor liver graft is collected by clean operation. The collected liver perfusate is collected in a 500 ml polypropylene (PP) centrifuge tube (500 ml tube). 500 ml tube is sealed with three layers of clean bags and placed in an ice-packed cooler box. The tubes are sealed in triplicate with clean bags and placed in an ice-packed cooler box for storage on ice during transport.<br>2 . Acceptance of cells<br>A 500 ml tube containing liver perfusate collected in the operating room is received by a quality control person or a cell coordinator via a pass box in the Cell Therapy Section of the Center for Frontier Medical Sciences, Hiroshima University Hospital. Processing of the received liver perfusate begins immediately.<br>3 . Cell Processing<br>All cell processing operations are performed in the Cell Therapy Room, Center for Frontier Medical Sciences, Hiroshima University Hospital according to the standard procedures. 500 ml tubes are centrifuged, and the supernatant is discarded in a safety cabinet for biohazard prevention. After diluting the blood cell component with saline, extract the lymphocyte fraction by centrifugation using specific gravity centrifugation with a sterile reagent for mononuclear cell separation (Ficoll-Paque PREMIUM). After confirming the number of cells and viability of the liver-derived lymphocytes, suspend the liver-derived lymphocytes in cell medium (X-VIVO medium) mixed with human IL-2 (Interleukin-2) preparation (ImmunesNote 35) and donor plasma (final concentration 2%)$_2$. Before starting culture, collect a portion of the cells and perform endotoxin test and bacterial culture test. One day before collection, mix the cell culture medium with anti-CD3 antibody according to GMP (good manufacturing practice) in order to remove the CD3 positive T cell fraction. At this time, a portion of the culture supernatant is also collected for endotoxin test and bacterial culture test.

	On the third day of culture, the cell suspension is centrifuged and washed with saline. Check the number of cells and viability after culture. At this time, the cell status, sterility test by Gram staining, endotoxin test, mycoplasma negative test, and bacterial culture test are performed. Suspend the cell suspension to be administered in albumin-containing saline.<br>４．Cell shipment<br>The clinical research manager and the quality manager shall make the shipping judgment of the test article. For the infection test, the results of endotoxin test and bacterial culture test at the start of culture, as well as the results of cell condition and Gram stained sterility test method at the time of cell collection shall be used for the shipment judgment.<br>５．Cell administration<br>On the third postoperative day after liver transplantation, with the permission of the recipient's primary physician, cell suspension is administered to the liver transplant recipient intravenously over a period of 30 minutes to 1 hour. The cells are administered only once, on the third postoperative day.
Concomitant use of prohibited drugs and therapies	None in particular
Outline of the observation and inspection schedule	Refer to the Observation and Inspection Schedule
Target number of registered subjects	37 cases<br>＜Basis for setting<br>In a previous study comparing the incidence of bacteremia at 1 month after living donor liver transplantation in 21 patients in the NK therapy group and 21 patients in the matched non-NK therapy group for 114 living donor liver transplants performed from January 2004 to December 2009, the incidence of bacteremia in the NK therapy group was 10% compared to 30% in the non-NK therapy group. The incidence of bacteremia in the NK therapy group was 10% compared to 30% in the non-NK

	therapy group (Tashiro et al., Transplantation, 2011).<br>Based on these results, the expected incidence of bacteremia in NK therapy at 1 month after living donor liver transplantation was set at 10%, and the number of cases required to examine the threshold of 30% incidence in non-NK therapy at 5% significance level (both sides) and 80% power was calculated to be 34. Considering the dropout cases at the time of enrollment, we set the target number of cases at 37.
Research period	Research period: From the date of research notification to December 31, 2024 (9 years)<br>Period of case registration: From the date of research notification to December 31, 2021 (6 years)

schema

試験デザイン

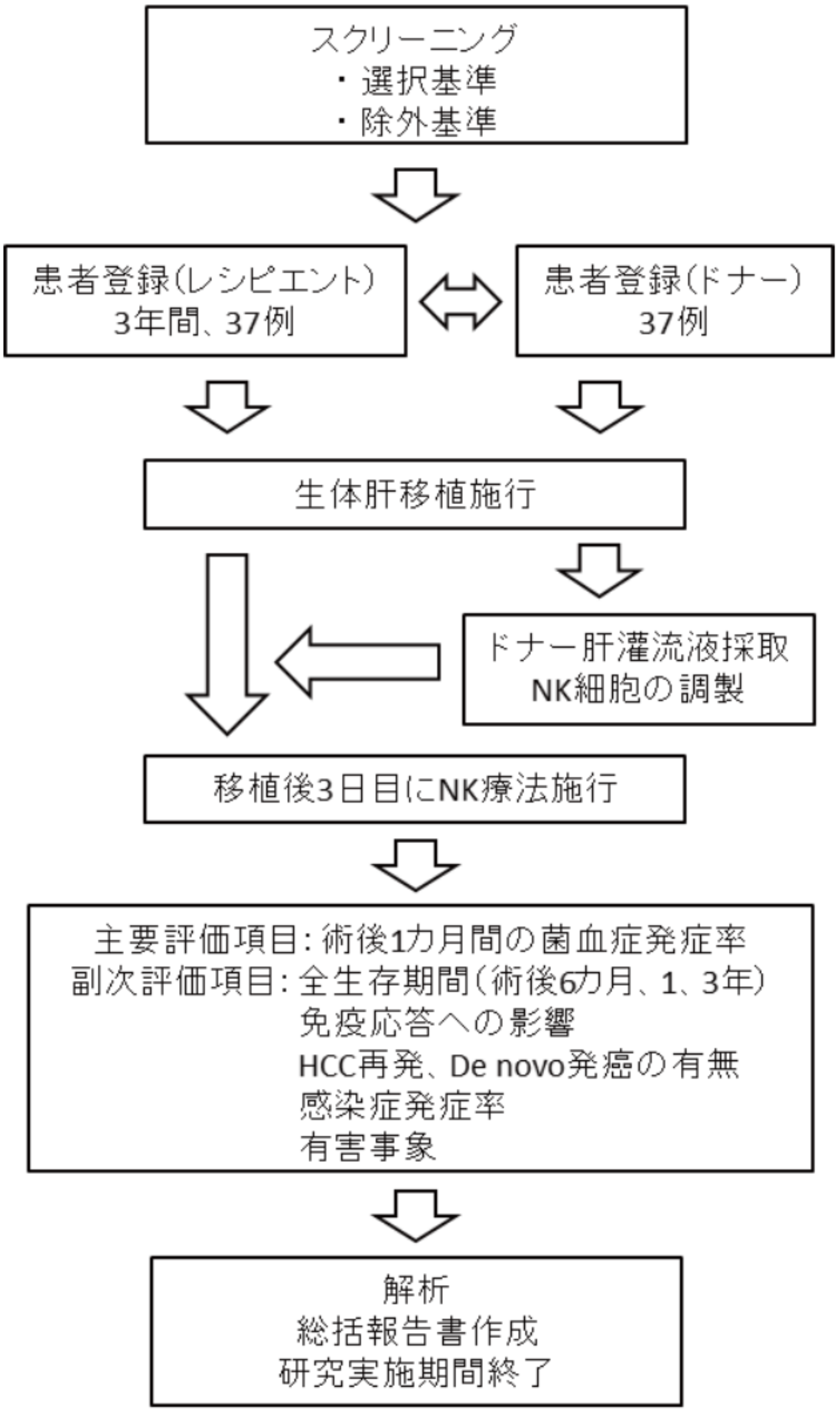

Observation and Inspection Schedule

Recipient.

観察日・評価日	同意取得	スクリーニング	術前検査	登録	手術日	細胞投与日	細胞投与日	4日後	5日後	1週間後	2週間後	4週間後	8週間後	12週間後	6か月後	以後6カ月毎3年まで
許容範囲	手術前4週間以内	手術前4週間以内	手術前4週間以内	手術前4週間以内		投与前	投与後	投与翌日	投与2日後	±2日	±2日	±1週	±1週	±1週	±8週	±8週
同意取得	○															
登録				○												
被験者情報		○			○											
臨床症状			○			○	○	○	○	○	○	○	○	○	○	○
バイタルサイン			○			○	○	○	○	○	○	○	○	○	○	○
血液検査			○			○	○	○	○	○	○	○	○	○	○	○
尿検査			○			○		○	○	○	○	○				
胸部Xp			○			○		○	○	○	○	○				
培養			○							○	○	○				
CT			○									○		○	○	○
フローサイト			○			○				○	○	○				
MLR			○							○	○	○				
有害事象							○	○	○	○	○	○	○	○	○	○
併用治療							○	○	○	○	○	○	○	○	○	○

Cell donor (donor)

Date of observation and evaluation	Obtaining Consent	preoperative scan	Registration	procedure
tolerance level	Within 4 weeks of surgery			
Obtaining Consent	p-sound			
Subject information *		p-sound		
clinical condition (of a patient's illness) *		p-sound		
vital signs *		p-sound		
blood test *		p-sound		
Collection of donor graft liver perfusion				p-sound

*: If the patient has been observed within 3 months prior to surgery to confirm the suitability for living donor liver transplantation, the observation and examination data will be used.

definition of a word

▪ Liver-derived natural killer (NK) cells

The liver is known to have a much higher content of various lymphocytes in its tissues, especially NK cells, than other organs. We have reported that liver-derived NK cells collected from normal liver have high antitumor activity against hepatocellular carcinoma cell lines.

▪ Vivo partial liver transplantation

Partial liver transplantation from a donor after removal of the recipient's entire liver is a fundamental treatment for non-compensated liver failure. In many other countries, brain-dead liver transplantation from brain-dead donors is common, but in Japan, partial liver transplantation from living donors is the most common procedure because of the low number of organ donations from brain-dead donors.

▪ Test article preparation facility

A facility with the structure, equipment, and management system for aseptic preparation of donor liver-derived lymphocytes, the test article, in accordance with the standard procedures. In this clinical study, the test material used for the protocol treatment will be prepared in the Center for Translational Medicine, Hiroshima University Hospital.

Background (plain language).

For patients with end-stage liver failure, liver transplantation is the only treatment available. When a patient undergoes a liver transplant surgery, he or she needs to take a drug called immunosuppressant to prevent rejection. Immunosuppressive drugs lower the body's resistance to infection, making it more susceptible to infections caused by germs and viruses. Especially right after the surgery, you need a lot of immunosuppressive drugs, which increases the probability of getting a life-threatening infection.

Content (in plain language).

The liver of a donor used in a liver transplant contains many cells (natural killer cells) that have a strong ability to attack cancer and viruses. During the liver transplant surgery, these cells are removed and cultured for three days to enhance their attacking power. On the third day after the liver transplant, the activated natural killer cells are injected into the recipient. Natural killer cells are relatively resistant to immunosuppressive drugs and are expected to be effective in preventing infections immediately after surgery.

1 Research Objectives

The purpose of this clinical study was to evaluate whether immunostimulatory therapy using activated natural killer (NK) cells derived from liver donor on day 3 after living donor partial liver transplantation can reduce the incidence of severe infections after liver transplantation in patients with uncompensated cirrhosis for whom no other treatment is available. This is an open-label study to compare the results. In this study, we will investigate the frequency of bloodstream infection (bacteremia) after liver transplantation and the safety of NK cell administration.

2 warp and woof

2.1 Target disease

Patients with uncompensated cirrhosis requiring liver transplantation

2.1.1 Concepts, definitions, etiology, and pathogenesis

Liver transplantation is the only curative treatment for uncompensated cirrhosis, and in recent years, post-transplant outcomes have improved significantly due to standardization of the procedure, advances in immunosuppressive agents, and improved perioperative management. In recent years, however, post-transplant outcomes have improved significantly due to standardization of surgical techniques, advances in immunosuppressive agents, and improved perioperative management. Bloodstream infection is a condition in which infection spreads from local to systemic and is defined as bacteremia with clinical symptoms (CDC guidelines). The prognosis of bacterial and fungal bloodstream infections is poor, especially in the early postoperative period under strong immunosuppression, and is one of the issues that must be overcome to further improve transplantation outcomes.

2.1.2 epidemiology

Sixty to eighty percent of liver transplant recipients will experience some type of infection within the first year after surgery. Most of these infections are curable, but some are fatal.(Colonna et al., Arch Surg, 123,p.360, 1988; Kawecki et al., Med Sci Monit, 15,p.CR628, 2009) The breakdown of infections is as follows About half of the infections are bacterial, 40% are viral, and 10% are fungal.(Fishman & Rubin, N Engl J Med, 338,p.1741, 1998;) This is especially true within the first month after transplant. In particular, the frequency of infections is highest within the first

month after transplantation. Bloodstream infections have been reported to be responsible for 30% of post-transplant deaths. The most common causes of bacteremia are central venous catheter infection (30%), cholangitis (30%), and intra-abdominal infection (23%), with Gram-positive cocci and Gram-negative rods being the most commonly detected organisms.( ) The most common species detected are gram-positive cocci and gram-negative rods.

2.1.3 Standard treatment and prognosis

Prophylactic antibacterial and antifungal therapy for bacterial and fungal infections after liver transplantation has little evidence based on randomized clinical trials, and there is no consensus on the choice of drugs and methods. Although the drug of choice varies by institution, most institutions use a single 2-3 generation cephalosporin for 2-3 days of prophylaxis.(Soave, Clin Infect Dis, 33 Suppl 1,p.S26, 2001) The choice of drug varies by institution, but most institutions use a single 2-3 generation cephalosporin for 2-3 days of prophylaxis.

2.1.4 Basis for setting target diseases

Infectious diseases are the leading cause of death after liver transplantation, and the objective of this study is to reduce deaths due to infectious diseases. The aim of this study is to reduce the number of deaths due to infections. Since bacteremia is generally regarded as a serious prognostic factor among infections after liver transplantation, this clinical study will focus on bacteremia in the early postoperative period.

2.2 Name of the specified cell processing product and its outline

2.2.1 Name of specified cell processing product

Activated natural killer (NK) cells from liver transplant donor livers

2.2.2 Overview of the test object

For living donor partial liver transplantation, transportal hepatic perfusion of the grafted liver is mandatory for prevention of GVHD (graft-versus-host disease) and organ preservation. The liver perfusion fluid, which is usually discarded, contains a much higher concentration of liver-derived lymphocytes (T cells, B cells, NK cells, and NKT cells) compared to peripheral blood. By culturing these liver-derived lymphocytes in the presence of IL-2 (100 IU/ml) for 3 days, it is possible to induce proliferation and activation of the NK cell fraction. Activated NK cells contained in

activated intrahepatic lymphocytes have been confirmed to have high cytotoxic activity against hepatoma cell lines in vitro. In addition, 70% hepatectomy in mice induces a decrease in the expression of cytotoxic molecules of intrahepatic NK cells, and when a hepatocellular carcinoma tumor line is administered through the transportal vein, cancerous tissue is observed to grow in the liver (intrahepatic metastasis model). We have confirmed that NK cells in the perfusate of the liver of normal mice can be activated and administered to the mice to inhibit cancer growth. Since NK cells are the most abundant fraction of activated liver-derived lymphocytes, but there is also a T cell fraction that can induce GVHD, anti-CD3 antibody (1 μg/ml) is added to the culture medium 1 day before administration to remove CD3-positive T cells.

The advantages of these cells are the use of donor liver perfusate, which is essential for surgical procedures and is usually discarded, and the ability to preferentially grow NK cells, which are highly cytotoxic as well as active, by simple in vitro culture. Therefore, cell collection is not invasive, and clinical application is relatively easy.

2.2.3 Summary of results of preclinical studies, clinical studies, and clinical trials to date

preclinical study

- **TRAIL molecule-mediated cytotoxicity of intrahepatic NK cells against autologous hepatocytes in mice(Ochi et al., Hepatology, 39,p.1321, 2004)**

  In vitro cytotoxicity assays showed that mouse liver-derived NK cells were cytotoxic to autologous hepatocytes even in the naïve state. In vitro cytotoxicity assays showed that mouse liver-derived NK cells were cytotoxic to autologous hepatocytes even in naïve state, and their cytotoxic activity was further enhanced after Poly I:C stimulation. The cytotoxicity was attenuated by the administration of anti-TRAIL antibody. Mouse liver-derived TRAIL-positive NK cells lacked expression of Ly49, an inhibitory receptor that recognizes MHC class I. In addition, Poly I:C stimulation enhanced the expression of Ly49 on TRAIL-negative NK cells, but not on TRAIL-positive cells. It is thought that mouse liver-derived TRAIL-positive NK cells develop cytotoxicity against autologous hepatocytes due to decreased self-recognition, confirming the importance of the TRAIL molecule in the cytotoxic mechanism of NK cells.

- **Cytotoxicity of human intrahepatic NK cells against hepatocellular carcinoma(Ishiyama et al., Hepatology, 43,p.362, 2006)**

Hepatocyte-derived NK cells collected from human liver perfusate did not express TRAIL as well as peripheral blood NK cells under naïve conditions, but stimulation with IL-2 induced a higher level of TRAIL expression compared with peripheral blood NK cells. Human normal liver-derived NK cells showed higher cytotoxic activity against hepatocellular carcinoma cell lines when stimulated with IL-2 compared to peripheral blood- or cirrhotic liver-derived NK cells. In humans, IL-2 stimulation induced the expression of HLA class I inhibitory receptors as well as the expression of TRAIL. The expression of decoy receptor (DcR) 1/DcR2, which act competitively with death receptor (DR) 4/DR5, the functional receptors for TRAIL, was observed in normal liver tissue, whereas the expression of DR4/DR5 was enhanced with decreasing differentiation in hepatocellular carcinoma tissue. In contrast, in hepatocellular carcinoma tissues, the expression of DR4/DR5 was enhanced with decreasing differentiation, and the expression of DcR1/DcR2 was decreased.

In cytotoxicity studies, normal liver-derived NK cells after IL-2 stimulation showed little cytotoxicity against autologous and HLA haplotype-matched hepatocytes.

These results indicate that normal liver-derived NK cells may selectively damage hepatocellular carcinoma by IL-2 stimulation and suppress hepatocellular carcinoma recurrence after liver transplantation.

- **Inhibition of liver metastasis by TRAIL intrahepatic NK cells in a mouse liver metastasis model(Ohira et al., Transplantation, 82,p.1712, 2006)**

  In the mouse massive hepatectomy model, the number of TRAIL-positive NK cells in the liver was reduced, and the antitumor activity of liver-derived NK cells was decreased. However, intrahepatic metastasis was induced in the above-mentioned massive hepatectomy mouse model by transportal administration of hepatoma cell lines. However, intrahepatic metastasis was induced by transportal administration of hepatocellular carcinoma cell lines to the above-mentioned massive hepatectomized mice, and tumor growth was suppressed by simultaneous administration of activated liver-derived NK cells from mice stimulated with Poly I:C.

- **Inhibition of HCV (hepatitis C virus) infection by donor liver-derived activated lymphocytes in human liver chimeric mice(Ohira et al., J Clin Invest, 119,p.3226, 2009)**

  Lymphocytes obtained from human liver perfusate are rich in NK and NKT cells. Using the protocol of this clinical study, the addition of IL-2 (100 IU/ml) and anti-CD3 antibody (1ug/ml) to liver-derived lymphocytes showed potent anti-HCV effects in an assay using HCV replicon cells. In addition, administration of human liver-derived activated lymphocytes to HCV-infected human liver chimeric mice suppressed HCV infection. Furthermore, simultaneous administration of anti-IFN- γ(interferon gamma) antibodies counteracted the anti-HCV effect, indicating that IFN-γ was the responsible molecule.

- **NK cells obtained from liver perfusate of a brain-dead liver transplant donor have potent antitumor activity(Ohira et al., Cell Transplant, 21,p.1397, 2012)**

  In order to apply this clinical study to brain-dead liver transplant patients, liver perfusion fluid from brain-dead liver transplant donors was collected at the University of Miami, USA, and cell processing was performed at a cGMP facility. As in the case of living liver transplant donors, we confirmed that the cells were rich in NK cells and acquired potent antitumor activity upon IL-2 stimulation. In addition, good expression of TRAIL molecules, which efficiently attack hepatocarcinoma cells, was obtained, and the content of T cells, which cause GVHD, was low at 1.8×10^{4}cells/kg, which is an acceptable amount for

administration to allo. Based on these results, the U.S. Food and Drug Administration (FDA) has approved the initiation of Phase I trials.

clinical research

Based on the results obtained from the basic experiments, we started a safety study of donor liver-derived activated NK cell therapy for the control of hepatocellular carcinoma recurrence after liver transplantation in liver transplantation patients with hepatocellular carcinoma before the notification of "Guidelines for Clinical Research Using Human Stem Cells" on July 3, 2006. The clinical results are shown below.

- **Activated NK cell therapy for the prevention of hepatocellular carcinoma recurrence after living donor liver transplantation at Hiroshima University (paper in preparation)**

  Twenty-four patients with hepatocellular carcinoma (HCC) within the preoperative Milan criteria underwent donor liver-derived activated lymphocyte transfer as adjuvant immunotherapy after liver transplantation. The median age of the patients was 58 years, 17 were males and 7 were females. The median number of activated lymphocytes administered was 270 (38-820) $\times 10^6$cells/body. 5-year overall survival rate and 5-year relapse-free survival rate were 82.5% and 74.5%, respectively. No GVHD was observed in patients receiving immunotherapy, and no other serious dose-related adverse events were observed. There was no significant difference in the incidence of Grade 3/4 (CTCAE ver. 4) or higher adverse events between the high-dose group ($> 270 \times 10^6$cells/body) and the low-dose group ($\leq 270 \times 10^6$cells/body). There was no significant difference in the frequency of Grade 3/4 (CTCAE ver.4) or higher adverse events between the immunotherapy group and the non-immunotherapy group. The quantitative antidonor response by CFSE-MLR (lymphocyte mixing test using CFSE dye) did not differ between immunotherapy and non-immunotherapy groups.

- **Effect of postoperative adjuvant therapy with donor liver-derived activated lymphocytes on bloodstream infections after living donor liver transplantation(Tashiro et al., Transplantation, 92,p.575, 2011)**

  We analyzed the incidence of early postoperative bloodstream infections in 114 living donor liver transplant cases performed at Hiroshima University Hospital from 2004 to 2009. The frequency of bloodstream infections was

significantly lower in the immunosupplementation group compared with 21 patients who received immunosupplementation using activated lymphocytes derived from donor liver and 21 patients who did not receive immunosupplementation adjusted for background factors using the propensity score matching method.

- **Anti-HCV effect of postoperative adjuvant therapy using activated lymphocytes derived from donor liver (Ohira et al., J Clin Invest, 119,p.3226, 2009)**

  In this study, we compared the postoperative HCV viral load in seven HCV-infected liver transplant recipients who received activated NK cell therapy and five HCV-infected patients who underwent liver transplantation at the same time. In the group treated with NK cell therapy, the HCV viral load was significantly reduced for one month after surgery compared with the control group. However, although there was a strong anti-HCV effect immediately after immunotherapy, HCV-RNA gradually increased over time, suggesting that the effect may be temporary.

- Activated NK cell therapy for the prevention of hepatocellular carcinoma recurrence after brain-dead liver transplantation at the University of Miami, USA (paper in preparation)

  In collaboration with Hiroshima University and the University of Miami (Miami, FL, USA), we have been conducting a clinical trial (Phase I) of cell therapy using activated liver NK cells derived from brain-dead donors since July 2010. Eighteen brain-dead liver transplant recipients with unresectable hepatocellular carcinoma were included in the study. The median age of patients was 60 years, 16 males and 2 females, and no serious adverse events, including GVHD, were observed. 9 of the 18 patients exceeded the Milan criteria for postoperative pathological diagnosis, but none had HCC recurrence at the mean observation period of 31 months. The survival rate of patients treated with NK therapy was better than that of the historical control at the University of Miami.

2.2.4 Reasons for judging that it is possible to conduct clinical research

Regarding the safety of postoperative adjuvant immunotherapy using activated lymphocytes derived from donor livers, no adverse events associated with cell transfer were observed clinically in Phase I clinical trials initiated before the

implementation of the Ministry of Health, Labor and Welfare's "Guidelines for Clinical Research Using Human Stem Cells. In addition, in preclinical studies, it has been shown to improve the prognosis of recurrence in patients who are within the preoperative Milan criteria and deviate from the pathological Milan criteria, as well as to suppress the frequency of bloodstream infection in the early stage after liver transplantation, suggesting that it may contribute to the prognosis of survival after living donor liver transplantation. Severe infections, including bloodstream infections, remain one of the most common life-threatening acute complications after liver transplantation. Even though various measures have been taken to prevent infection, there are still many cases in which severe infections cannot be avoided due to poor preoperative patient conditions or the use of immunosuppressive drugs, and effective prevention is expected. In view of the above, we judged that it could be implemented in clinical research.

2.3 Anticipated benefits and disadvantages for enrolled patients

2.3.1 Projected profit

This clinical study was conducted to evaluate the clinical efficacy of activated NK cells contained in activated lymphocytes derived from donor liver after living donor liver transplantation. Although living donor liver transplantation has become popular as the only fundamental treatment for uncompensated cirrhosis, its prognosis is extremely poor if complicated by severe infections under severe surgical invasion and postoperative immunosuppression. If the clinical efficacy of donor liver-derived activated NK cells after living donor liver transplantation in this clinical study is confirmed, the occurrence of severe infections will be reduced, and this will greatly contribute to the postoperative prognosis of living donor liver transplantation.

The subjects will not receive any compensation or any other benefits by participating in this clinical research. In addition, any intellectual property rights arising from this clinical research will belong to the researcher, and the subject will not receive any benefit other than the therapeutic effect.

2.3.2 Anticipated disadvantages

In addition to the adverse events listed in "Anticipated adverse events," subjects in this clinical study may experience unexpected adverse events, which will be dealt with promptly with appropriate measures and treatment even after the end of the study period. There is a risk of false negative results in the donor infection test due to window period, and if infection is found, appropriate measures will be taken.

The cost of liver transplantation is borne by the patient as a normal medical treatment, but the cost of treatment in this clinical study will be covered by research funds and will not be borne by the subject. For the donor of the test material, the living donor liver transplant, there is no risk except for the risk associated with the donor hepatectomy because the perfusion fluid that is normally discarded is collected and used.

2.4 Significance of this clinical study

The significance of this clinical study is to clarify the clinical efficacy of using donor liver-derived activated NK cells after living donor liver transplantation on the third day after living partial liver transplantation for patients with uncompensated liver cirrhosis for whom there is no treatment other than liver transplantation, and to improve the results of living donor liver transplantation. After confirming the clinical efficacy and safety of donor liver-derived activated NK cells after living donor liver transplantation in this clinical study, we aim to further verify the efficacy of this treatment method as an advanced medical treatment B.

This is expected to ultimately contribute significantly to improving the prognosis of liver transplant patients.

3 Target diseases and eligibility criteria

3.1 Target disease

Patients with uncompensated cirrhosis requiring living donor liver transplantation

3.2 selection criteria

○ Selection criteria for recipients (those who receive regenerative medicine)

Select patients who meet all of the following criteria.

1. Patients undergoing living partial liver transplantation for the treatment of non-compensated cirrhosis refractory to medical therapy
2. Patients aged 20 years or older at the time of obtaining consent
3. Patients whose written consent to participate in this study has been obtained from the patient or a substitute.

Basis for setting selection criteria for recipients (recipients of regenerative medicine)

1. This clinical study was set as a selection criterion to evaluate the therapeutic efficacy in preventing the development of severe infections after living donor liver transplantation in patients with uncompensated cirrhosis.
2. We did not think it necessary to set an upper age limit for subjects who would be able to undergo living donor liver transplantation, so we set the age at 20 years or older.
3. It was set up to incorporate patients who understand clinical research and can cooperate with clinical research throughout its duration, taking ethics into consideration.

○ Selection criteria for cell donors

Select patients who meet all of the following criteria.

1. Those who meet the criteria of the Liver Transplant Study Group's "Guidelines for Living Donor Surgery" and undergo living donor surgery as liver donors.
2. Those who are at least 20 years old at the time of obtaining consent
3. A person who has given written consent to the preparation of liver-derived natural killer (NK) cells from the perfusate of a donor liver graft and their administration to the recipient.

[Basis for setting criteria for selecting cell donors (donors)

1. The cells used in this clinical study were collected from within the grafted liver during liver transplantation surgery, and the liver donor was set to be a person undergoing living donor liver transplantation surgery.
2. For cell donors, who are assumed to be able to undergo the living donor liver transplantation procedure, the age of 20 years or older was chosen to guarantee safety.
3. To incorporate donors who fully understand this clinical study of administering liver transplant donor liver-derived activated natural killer cells to recipients and who can cooperate with the study, taking into account ethical considerations

3.3 exclusion criteria

○ Exclusion Criteria for Recipients

Patients who meet any of the following criteria will be excluded from the study.

1. Patients undergoing re-liver transplantation
2. Patients undergoing brain-dead liver transplantation

3. Other patients whose participation in this clinical research is deemed inappropriate by the principal investigator or sub-investigator.

(Basis for establishing exclusion criteria for recipients)

1. Because treatment with initial liver transplantation will affect the evaluation of this clinical study
2. Because it is not possible to collect and adjust test materials according to this test protocol.
3. To promote research and take appropriate and flexible measures with attention to the dignity of patients

○ Exclusion criteria for cell donors (donors)

Those who fall under any of the following items shall be excluded from the scope of the program.

1. Persons undergoing living donor liver transplant surgery for re-liver transplantation
2. A person who is judged by the principal investigator or sub-investigator to be inappropriate to participate in this clinical research.

The basis for setting the exclusion criteria for cell donors.

1. Because treatment with initial liver transplantation will affect the evaluation of this clinical study
2. To promote research and take appropriate and flexible measures with attention to the dignity of patients

4 Obtaining Consent

4.1 Preparation of consent explanatory documents and consent forms

Principal investigators will prepare consent explanatory documents, consent forms, and consent withdrawal forms to be used to obtain consent for research participation from subjects (donors and recipients) and their family members or other surrogates, using plain language and terminology whenever possible.

The principal investigator will provide the subjects (donors and recipients) and their alternates with a consent explanation document approved by the Hiroshima University Regenerative Medicine Committee, provide sufficient written and oral

explanations, and obtain the research subjects' free and voluntary consent in writing.

4.2 Revision of the consent explanatory document and consent form

When information on efficacy or safety is obtained that may affect the consent of subjects (donors and recipients), or when changes are made to the research plan that may affect the consent of subjects (donors and recipients), the principal investigator will promptly provide information to the subjects (donors and recipients) and their alternates. In addition, the consent explanatory document, etc. will be revised with the prior approval of the Hiroshima University Regenerative Medicine Committee, and re-consent will be obtained. This does not apply to subjects (donors and recipients) for whom clinical research has already been completed.

4.3 Time and method of consent explanation and consent acquisition

The principal investigator or sub-investigator shall provide and use the consent explanatory document approved by the Hiroshima University Committee for Regenerative Medicine, etc., to subjects (donors and recipients) who are candidates for participation in this clinical research and their substitutes, and after providing sufficient oral explanation, obtain their consent for participation in this clinical research in The consent for participation in this clinical research shall be obtained in writing. After confirming that the subjects (donors and recipients) and their substitutes have a good understanding of the contents of this clinical research, free consent will be obtained in writing before the screening test is conducted.

5 Registration

5.1 Subject Registration

After obtaining written consent, the principal investigator or sub-investigator shall confirm the eligibility of subjects and register them according to the following procedures

1. Preparation of the Subjects' List

   The principal investigator, sub-investigator, or research collaborator reports patients who have given written consent to participate in clinical research to the research secretariat, and

the personal information manager assigns a linkable anonymized subject identification code and collects only the data in an anonymized form. The personal information manager will manage the personal identification information and the correspondence table on a personal computer that is independent of the outside world, set a password that only the personal information manager knows, and store the computer in a room with strict security, thereby taking safety measures against information leakage. In addition, the correspondence chart will not be provided to outside parties.

2. Determination of eligibility

The principal investigator or sub-investigator will conduct screening and preoperative examinations after obtaining consent. Based on the patient background and the results of the preoperative examination, it will be confirmed that the patient meets all of the selection criteria at the time of enrollment as specified in "Target Diseases and Eligibility Criteria" and does not fall under any of the exclusion criteria.

3. Registration of subjects

To register a subject, enter the necessary information according to the case report form, and the research secretariat will confirm the subject and register him/her.

4. Initiation of Protocol Treatment

The principal investigator or sub-investigator will begin collection and adjustment of test materials along with the execution of the living donor liver transplantation procedure after receiving a report on the completion of enrollment from the research secretariat.

6 Specified cell products used in research

6.1 Name of specified cell processing product

Activated natural killer (NK) cells from liver transplant donor livers

6.2 Composition, structure, characteristics, and manufacturing method

6.2.1 Standard

1） Number of cells: 10 10 $\times^{6}$cells or more

2） Trypan blue dye exclusion test Cell viability: 80% or more

3 ） Gram stain*: negative

4） Endotoxin test: 5.0 EU/mL or less

*： 3) is based on the test results of the culture supernatant on the day of shipment.

6.2.2 Manufacturing method

All preparations of test materials used in the cell transplantation group will be in accordance with the separately established standard operating procedures.

Collection of donor liver perfusate

Transportal intrahepatic perfusion is performed on the donor graft according to the procedures established by the protocol treatment provider, and the perfusion drainage fluid is collected by clean handling, immediately stored in a specimen transport container, and transported to the test article preparation facility. During cell collection, no changes in medical procedures, surgery, or other treatment strategies will be made with priority given to cell collection.

Preparation of cells

Cell preparation should be performed by cell conditioning personnel who have received training in the use of the test article preparation facility, according to the predetermined procedures. Centrifuge 500 ml tubes containing liver perfusion solution and discard the supernatant in a safety cabinet for biohazard prevention. After diluting the blood cell component with saline, extract the lymphocyte fraction by centrifugation using specific gravity centrifugation with a sterile reagent for mononuclear cell separation (Ficoll-Paque PREMIUM). After confirming the number of cells and viability of the liver-derived lymphocytes, suspend the liver-derived lymphocytes in cell medium (X-VIVO medium) mixed with human IL-2 preparation (Immunes Note 35) and donor plasma (final concentration 2%)$_2$. Before starting culture, collect a portion of the cells for endotoxin test and bacterial culture test. One day before collection, mix the cell culture medium with anti-CD3 antibody (Miltenyi) according to GMP for the purpose of removing the CD3 positive T cell fraction. At this time, a portion of the culture supernatant is also collected for endotoxin test and bacterial culture test. On the third day of culture, the cell suspension is centrifuged and washed with saline. Check the number of cells and viability after culture. At this time, the cell status, sterility test by Gram staining, endotoxin test, mycoplasma negative test, and

bacterial culture test are performed. Suspend the cell suspension to be administered in albumin-containing saline.

Infectious Disease Testing

Submit the culture medium at the time of liver-derived lymphocyte isolation for aerobic culture test, anaerobic culture test, and endotoxin test. Submit 2 ml of cell culture medium to culture test again 12 hours before administration on the second day of culture. On the day of administration, dispense 2 ml from the cell culture supernatant to the culture test, and confirm the presence of infection by Gram staining in the culture laboratory, and make a judgment based on the results of the infectious disease test submitted during the culture process. A mycoplasma negative test, endotoxin test, and culture test will be submitted from the culture supernatant on the day of shipment, but will not be included in the judgment of shipment.

6.3 Container, packaging and storage conditions

Label the container containing the specified cell processing product with a label indication that can be identified by the subject, and store it at room temperature until the shipping decision based on the results of the test inspection is completed.

After the shipping decision is made, the container containing the specified cell processed product is packaged and shipped.

6.4 Delivery and transport

The container containing the specified cell products with labels attached is packaged, removed from the cell culture and processing facility, and transported or prepared for transport. The person in charge of the transport transports the specified cell products in the donor transport container to the site of the transplantation surgery. After arriving at the transplantation site, the person in charge of transportation hands over the specified cell products to the person in charge.

6.5 Management and storage

Upon arrival at the transplantation site, the physician in charge confirms and receives the number used to identify the subject, the number on the label of the specified cell processing product, and the quality assurance certificate. The specified cell products shall be stored in the designated area of the transplantation site until

use. The expiration date for use shall be within the day of shipment.

7 Test Design

7.1 Test Design

Single-center, historical data-controlled, open-label study

Basis for Design Settings]

This clinical study is a Phase I/II trial of immunostimulatory therapy using activated natural killer (NK) cells derived from liver transplant donors to prevent severe infections after liver transplantation. The safety and efficacy of the administration of liver-derived activated lymphocytes from transplant grafts have been confirmed in a previous Phase I study and in a Phase I study conducted by a collaborative research group in the U.S. in brain-dead liver transplant patients. In this study, based on the results of the previous studies, we set a threshold of 30% incidence of bacteremia in the non-NK group, and conducted an open-label study to ensure that the incidence of bacteremia (percentage) during NK therapy was below the threshold and to collect more safety information during NK therapy.

As for the number of cells to be transplanted, we decided not to set an upper limit because the preclinical studies have shown that the effect depends on the number of cells administered, but there is no difference in the occurrence of adverse events depending on the amount of cells administered, and the number of cells available depends on the donor liver graft, which is difficult to control. In addition, the number of cells available depends on the donor liver graft and is difficult to control.

7.2 Target number of enrolled subjects and enrollment period

Target number of enrolled subjects: 37 cases

In a previous study comparing the incidence of bacteremia at one month after living donor liver transplantation in 21 patients in the NK therapy group and 21

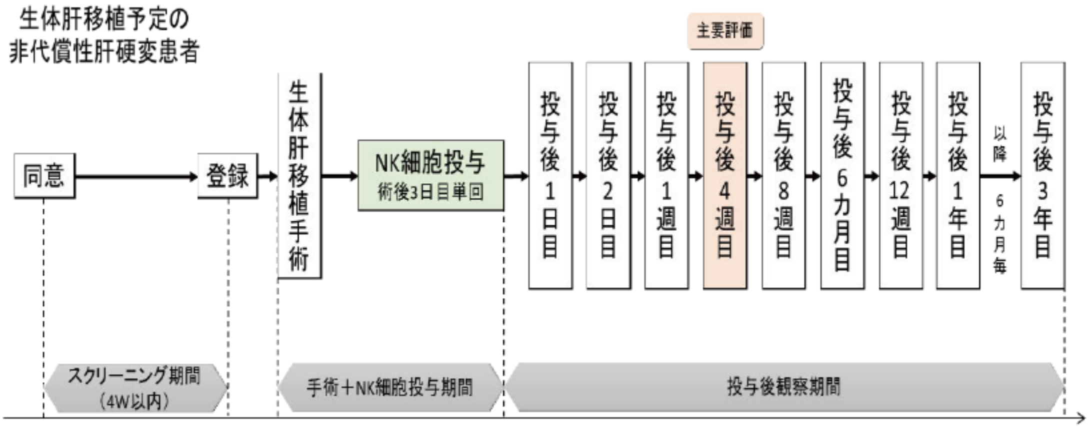

patients in the matched non-NK therapy group among 114 living donor liver transplants performed from January 2004 to December 2009, the incidence of bacteremia in the non-NK therapy group was 30%, while that in the NK therapy group was 10% (Tashiro et al., Transplantation, 92,p.575, 2011). (Tashiro et al., Transplantation, 92,p.575, 2011). Based on these results, the primary endpoint of this study was the incidence of bacteremia at 1 month after living donor liver transplantation, and the expected incidence rate in the NK therapy group was set at 10%. The number of patients needed to be below the threshold of 30% incidence in the non-NK therapy group at 5% significance level (both sides) and 80% power was calculated to be 34. Considering the dropout cases at the time of enrollment, we set the target number of cases at 37.

Subject enrollment period: From the date of notification of the study to December 31, 2021 (3 years)

7.3 Possibility of accumulating the target number of enrolled subjects

The total number of liver transplant cases performed at Hiroshima University Hospital from 2008 to 2012 was 97. Since Hiroshima University Hospital performs about 15 liver transplant surgeries every year, if we estimate that the number of patients eligible for this study is about 90% of the above cases, we can expect to obtain about 40 subjects during the 3-year enrollment period, and 37 subjects can be enrolled during the 3-year period even if we take into account the acquisition of consent for participation in the study.

7.4 Protocol Treatment Plan

7.4.1 Definition of Protocol Treatment

Lymphocyte fractions are extracted from donor graft liver perfusion effluent collected at the time of living partial liver transplantation surgery by the specific gravity centrifugation method. The liver-derived lymphocytes are cultured in complete medium with IL-2 (100 IU/ml) and donor plasma (final concentration 2%) for 3 days. One day prior to administration, anti-CD3 antibody (1 μg/ml) is added to the medium to remove CD3 positive T cells. The cell suspension is collected, centrifuged, and washed with saline, and then the cells are suspended in albumin-containing saline and administered as a single dose to the liver transplant recipient

transvenously on postoperative day 3. Protocol treatment is defined as from donor graft liver perfusion to the completion of donor liver-derived activated NK cell administration.

7.4.2 Method

1) Preparation of Activated Natural Killer (NK) Cells from Liver Transplant Donor Livers
It is shown in the section "Manufacturing Methods.

2) Administration of activated natural killer (NK) cells derived from liver transplant donor livers
After culture and confirmation of safety by infectious disease testing, a single dose of cell suspension is administered intravenously over 30 minutes to 1 hour to liver transplant recipients on day 3 after living partial liver transplantation.

7.4.3 combination therapy

Treatment considered necessary before and after living donor liver transplantation surgery should follow the usual postoperative management of living donor liver transplantation performed at Hiroshima University Hospital, and can be performed at any time.

7.4.4 Infectious Disease Treatment

Treatment of infectious diseases is left to the judgment of the physician in charge, based on a comprehensive assessment of the site, extent, and tolerability of treatment in each case.

7.4.5 Rationale for establishing a protocol treatment plan

Since this clinical study is not expected to have any impact on the surgical management of living partial liver transplantation, all procedures other than this postoperative adjuvant immunotherapy will be performed in accordance with the conventional perioperative management.

7.4.6 Duration of study participation of enrolled subjects

The treatment period is from donor graft liver perfusion to the completion of donor liver-derived activated NK cell administration, and the observation period is up to 3 years after living partial liver transplantation surgery.

7.5 in-progress total

An interim tally will be made at the end of the observation period of one month after the surgery in the 15th case in which NK cells were administered, and the Effectiveness and Safety Evaluation Committee will consider whether the study should be continued. The Efficacy and Safety Evaluation Committee will determine whether or not to continue the study, based on the achievement of the primary endpoint and from the safety perspective, if the incidence of bacteremia within 1 month after surgery is 6 or more cases (40% or more) out of 15 cases, and if the incidence of adverse events of Stage 3 or higher that cannot be ruled out as causally related to NK cell administration is 2 or more cases. The efficacy and safety evaluation committee will consist of three physicians, excluding the research subcontractor.

8 Primary and secondary endpoints

8.1 Primary endpoints

Incidence of bacteremia during the first month after surgery

Basis for setting primary endpoints

The onset of bacteremia, a severe infection after liver transplantation, is more likely to occur within one month after surgery (63%) based on the results of previous studies. Since the administered NK cells were confirmed in the recipient's body for up to one month, we chose one month as the period to evaluate the inhibition rate of bacteremia development by NK cell therapy.

8.2 Secondary endpoints

① Overall survival (6 months, 1, and 3 years postoperatively)

② adverse event

Type and severity of adverse events that occurred in this clinical study, frequency of occurrence, duration of occurrence, and causal relationship

③ Effect on immune response

Evaluation of donor specific immunoreactivity by CFSE-MLR (1, 2, 3, 4 weeks postoperatively), detection of donor specific antibody (DSA) (annual screening), incidence of rejection, evaluation of NK cell activity in recipient peripheral blood

④ Presence and timing of hepatocellular carcinoma recurrence (hepatocellular carcinoma cases only) and De novo carcinogenesis

⑤ Incidence of infectious diseases (bacterial infections, cytomegalovirus infections, fungal infections, analysis by genetic polymorphism)

Rationale for setting secondary endpoints

In order to re-evaluate the efficacy and safety of this treatment, the following secondary endpoints were included

① To evaluate its efficacy in long-term survival after liver transplantation

② To re-evaluate the safety of this treatment

③ To evaluate the effect of this treatment on the immune system of the recipient

④ To evaluate the effect of this treatment on carcinogenesis

⑤ To evaluate the effect of this treatment on infections other than bacteremia, and to evaluate the difference in the effect of genetic polymorphisms.

9 Observation and inspection items and schedule

9.1 Inspection Schedule

The schedule for conducting observations, examinations, and evaluations is shown in Table 1 and Table 2 below. The principal investigator and subcontractors will conduct observations, examinations, etc. according to the schedule. Items that can be conducted by research collaborators, such as subject information (background investigation) and clinical examinations, may be conducted by research collaborators under the supervision of the principal investigator.

Table 1: Observation and examination schedule (Subject: Recipient)

観察日・評価日	同意取得	スクリーニング	術前検査	登録	手術日	細胞投与日	細胞投与日	4日後	5日後	1週間後	2週間後	4週間後	8週間後	12週間後	6か月後	以後6カ月毎3年まで
許容範囲	手術前4週間以内	手術前4週間以内	手術前4週間以内	手術前4週間以内		投与前	投与後	投与翌日	投与2日後	±2日	±2日	±1週	±1週	±1週	±8週	±8週
同意取得	○															
登録				○												
被験者情報		○			○											
臨床症状			○			○	○	○	○	○	○	○	○	○	○	○
バイタルサイン			○			○	○	○	○	○	○	○	○	○	○	○
血液検査			○			○	○	○	○	○	○	○	○	○	○	○
尿検査			○			○		○	○	○	○	○				
胸部Xp			○			○		○	○	○	○	○				
培養			○							○	○	○				
CT			○									○		○	○	○
フローサイト			○			○				○	○	○				
MLR			○							○	○	○				
有害事象							○	○	○	○	○	○	○	○	○	○
併用治療							○	○	○	○	○	○	○	○	○	○

Table 2: Observation and Inspection Schedule (Subject: Donor)

Date of observation and evaluation	Obtaining Consent	preoperative scan	Registration	procedure
tolerance level	Within 4 weeks of surgery			
Obtaining Consent	p-sound			
Subject information *		p-sound		
clinical condition (of a patient's illness) *		p-sound		
vital signs *		p-sound		
blood test *		p-sound		
Collection of donor graft liver perfusion				p-sound

*: If the patient has been observed within 3 months prior to surgery to confirm the suitability for living donor liver transplantation, the observation and examination data will be used.

9.2 Observation and inspection items

In this study, the donor (the cell donor) and the recipient (the recipient to whom the specific cell product will be administered) are the subjects. However, since the purpose of this study is to evaluate the efficacy and safety of the administration of the specific cell product in the recipient, the recipient is the one who will be measured in the observation and inspection items unless otherwise specified. Subjects.

9.2.1 screening test

After written consent is obtained, observations and examinations will be conducted for the following items.

① Subject information: Subject background (date of birth, gender, height, weight, preoperative status, primary disease, ascites, encephalopathy, preoperative dialysis, preoperative desensitization), current medical history, previous medical history, treatment history for infectious diseases (specific

treatment), treatment history for hepatocellular carcinoma (hepatocellular carcinoma cases only)

9.2.2 preoperative scan

① Clinical manifestations: symptoms of infection, presence of treatment for infection, other clinical manifestations

② Vital signs: blood pressure, pulse rate, body temperature

③ Blood test

<Hematological tests

Red blood cell count, white blood cell count, neutrophil count, lymphocyte count, hemoglobin count, hematocrit count, platelet count

Biochemical tests

Total bilirubin, Indirect bilirubin, ALB, AST, ALT, γGTP, ALPH, CRP, BUN, Cre, Na, K, Cl, IgG, IgM, PT, PT-INR, AFP, L3 fraction, PIVKA-II, HBsAg, HBsAb, HBV-DNA, HCV, HCV-RNA, CMV-, IgG, CMV-IgM, EBV-IgG, EBV-IgM, -D glucan, MELD score, Child-Pugh classification IgG, CMV-IgM, EBV-IgG, EBV-IgM, β-D glucan, MELD score, Child-Pugh classification

④ Laboratory tests

Urinalysis: protein qualitative, sugar qualitative, occult blood qualitative

Blood type, HLA

⑤ Diagnostic imaging (number of tumors, maximum tumor diameter, localization)

Chest X-ray: preoperative diagnosis of hepatocellular carcinoma (hepatocellular carcinoma cases only)

Contrast-enhanced CT (MRI or US can be substituted in case of contrast media allergy or renal failure)

⑥ Culture test (bacteria species): nasal, urine

Criteria for submission of blood cultures (clinically suspicious findings of bacteremia): fever of 38° C or higher, chills, hypotension, clinical symptoms, two sets of blood cultures must be submitted.(Horan, Andrus, & Dudeck, Am J Infect Control, 36,p.309, 2008)

⑦ Immunological analysis

Peripheral blood flow cytometry (phenotyping): T cell %, NKT cell %, NK cell %, CD69 on NK cell, TRAIL on NK cell, NKp44 on NK cell, NKp46 on NK cell, cytotoxicity test

MLR (lymphocyte mixture test): CD4 T cell SI, CD8 T cell SI, CD4+CD25+T cell %, CD8+CD25+T cell %.

⑧ Cell donor information: date of birth, sex, relationship, height, weight, blood type, HLA, CMV-IgM, CMV-IgG, EBV-IgG, EBV-IgM, HBs-Ag, HBs-Ab, HBc-Ab, HCV-Ab, HIV-Ab, (for HBV, HCV, and HIV, the window (HBV, HCV, and HIV should be retested around 3 months after surgery due to the possibility of false negative results due to the window period).

[Observation period; from the time of donor's consent to the date of liver transplantation.]

9.2.3 Registration

Based on the results of screening and preoperative laboratory tests, subjects who meet all of the selection criteria at the time of enrollment as specified in "Target Diseases and Eligibility Criteria" and do not meet any of the exclusion criteria will be enrolled.

9.2.4 operating day

① Surgical information: graft type, graft weight, GRWR, total ischemia time, operation time, blood loss, blood transfusion volume

② Liver pathological findings: pathological Milan criteria (only for cases with liver cancer complications), background liver HIA score

③ Information on administered cells: collection of liver perfusate, culture of liver-derived lymphocytes, and liver perfusate flow cytometry

9.2.5 Day of NK cell administration (postoperative day 3)

[Before NK cell administration]

① Clinical manifestations: symptoms of infection, presence of treatment for infection, other clinical manifestations

② Vital signs: blood pressure, pulse rate, body temperature

③ Blood test

<Hematological examination>: Same as preoperative examination

Biochemical tests

Total bilirubin, Direct bilirubin, ALB, AST, ALT, γGTP, ALPH, CRP, BUN, Cre, Na, K, Cl, PT, IgG, IgM, CNI blood levels

④ Urinalysis: protein qualitative, sugar qualitative, occult blood qualitative

⑤ Immunological analysis

Peripheral blood flow cytometry (phenotyping): T cell %, NKT cell %, NK cell %, CD69 on NK cell, TRAIL on NK cell, NKp44 on NK cell, NKp46 on NK cell, cytotoxicity test

⑥ Diagnostic imaging: simple chest x-ray

[After NK cell administration.]

① Clinical manifestations: symptoms of infection, presence of treatment for infection, other clinical manifestations

② Vital signs: blood pressure, pulse rate, body temperature

③ Blood test

<Hematological examination>: Same as preoperative examination

Biochemical tests

Total bilirubin, Direct bilirubin, ALB, AST, ALT, γGTP, ALPH, CRP, BUN, Cre, Na, K, Cl, PT

④ Observation of adverse events

Adverse event name, date of occurrence, grade, severity, treatment, outcome/decision date, causal relationship, comments (details of decision to continue, causal relationship, course, etc.)

The name of the adverse event should be recorded by the diagnosis. The name of the adverse event should be recorded with the name of the diagnosis. However, symptoms and signs that cannot be given a diagnosis should be recorded with the name of the symptom or sign.

Check for infections, use of antibiotics, and rejection.

Patient outcomes (presence/absence of graft failure, presence/absence of death, cause of death), secondary transplantation (presence/absence, timing)

⑤ Confirmation of concomitant treatment: treatment for infections (including prophylactic and anticipatory, therapeutic agent, dose, duration)

9.2.6 1 or 2 days after NK cell administration (postoperative day 4 or 5)

① Clinical manifestations: symptoms of infection, presence of treatment for infection, other clinical manifestations

② Vital signs: blood pressure, pulse rate, body temperature

③ Blood tests

Hematological examination: Same as preoperative examination

Biochemical tests: Same as before NK cell administration on the day of NK cell administration (postoperative day 3)

④ Urinalysis: protein qualitative, sugar qualitative, occult blood qualitative
⑤ Diagnostic imaging: simple chest x-ray
⑥ Observation of adverse events
⑦ Confirmation of concomitant treatment

9.2.7 1, 2 weeks after NK cell administration

① Clinical manifestations: symptoms of infection, presence of treatment for infection, other clinical manifestations
② Vital signs: blood pressure, pulse rate, body temperature
③ Blood test

<Hematological examination>: Same as preoperative examination

Biochemical tests

Total bilirubin, Direct bilirubin, ALB, AST, ALT, γGTP, ALPH, CRP, BUN, Cre, Na, K, Cl, PT, IgG, IgM, β-D glucan, C7-HRP, CNI blood levels

④ Urinalysis: protein qualitative, sugar qualitative, occult blood qualitative
⑤ Diagnostic imaging: simple chest x-ray
⑥ Culture tests (bacterial species): blood, sputum, urine, bile, drains
⑦ Immunological analysis

Peripheral blood flow cytometry (phenotyping): T cell %, NKT cell %, NK cell %, CD69 on NK cell, TRAIL on NK cell, NKp44 on NK cell, NKp46 on NK cell, cytotoxicity test

MLR (lymphocyte mixture test): CD4 T cell SI, CD8 T cell SI, CD4+CD25+T cell %, CD8+CD25+T cell %.

⑧ Observation of adverse events
⑨ Confirmation of concomitant treatment

9.2.8 4 weeks after NK cell administration

① Clinical manifestations: symptoms of infection, presence of treatment for infection, other clinical manifestations
② Vital signs: blood pressure, pulse rate, body temperature
③ Blood test

<Hematological examination>: Same as preoperative examination

Biochemical tests: Same as 1 and 2 weeks after NK cell administration

④ Urinalysis: protein qualitative, sugar qualitative, occult blood qualitative

⑤ Diagnostic imaging: simple chest X-ray imaging, contrast-enhanced CT (MRI or US can be substituted in case of contrast agent allergy or renal failure)

⑥ Culture tests (bacterial species): blood, sputum, urine, bile, drains

⑦ Immunological analysis

Peripheral blood flow cytometry (phenotyping): T cell %, NKT cell %, NK cell %, CD69 on NK cell, TRAIL on NK cell, NKp44 on NK cell, NKp46 on NK cell, cytotoxicity test

MLR (lymphocyte mixture test): CD4 T cell SI, CD8 T cell SI, CD4+CD25+T cell %, CD8+CD25+T cell %.

⑧ Observation of adverse events

⑨ Confirmation of concomitant treatment

9.2.9 After 8 weeks and 12 weeks of NK cell administration

① Clinical manifestations: symptoms of infection, presence of treatment for infection, other clinical manifestations

② Vital signs: blood pressure, pulse rate, body temperature

③ Blood test

<Hematological examination>: Same as preoperative examination

Biochemical tests: Same as 1 and 2 weeks after NK cell administration

④ Observation of adverse events

⑤ Confirmation of concomitant treatment

9.2.10 6 months after NK cell administration, then every 6 months for 3 years

① Clinical manifestations: symptoms of infection, presence of treatment for infection, other clinical manifestations

② Vital signs: blood pressure, pulse rate, body temperature

③ Blood test

<Hematological examination>: Same as preoperative examination

Biochemical tests

Total bilirubin, direct bilirubin, ALB, AST, ALT, γGTP, ALPH, CRP, BUN, Cre, Na, K, Cl, PT, CNI blood levels

④ Diagnostic imaging: contrast-enhanced CT (MRI or US can be substituted in case of contrast agent allergy or renal failure)

⑤ Observation of adverse events

⑥ Confirmation of concomitant treatment

9.2.11 when suspended

① Reason for discontinuation

② Comment.

10 Ensuring the safety of subjects

10.1 basic facts

In order to ensure the safety of human subjects, principal investigators and sub-investigators shall comply with the following basic items

1. The principal investigator or sub-investigator shall comply with the selection and exclusion criteria for subjects.
2. When a subject receives treatment from a physician other than the principal investigator and sub-investigator of this clinical research, the subject shall notify the physician of his or her participation in this clinical research and the nature of this research.
3. After the completion of this clinical study, patients will be examined for as long as possible and attention will be paid to the occurrence of adverse events.
4. If subjects feel any abnormality in their health, they will be instructed to immediately contact the principal investigator or sub-investigator.
5. When the principal investigator or sub-investigator recognizes that an adverse event has occurred in a subject and that treatment is necessary, the principal investigator or sub-investigator should inform the patient concerned, provide appropriate medical care, and describe the event without discrepancy in the case report form.
6. The principal investigator shall summarize the occurrence of adverse events in a status report and submit it to the Hiroshima University Committee on Regenerative Medicine once a year, and report it to the administrator.

10.2 Definition of Adverse Events

An "adverse event" is any unwanted or unintended sign (including abnormal changes in general laboratory values), symptom, or illness that occurs while a subject is participating in a clinical study, whether or not causally related to the protocol treatment. Clinically significant worsening of symptoms that were present prior to protocol treatment is also an adverse event. Physiological changes that are not considered clinically objectionable in terms of frequency or degree are not considered adverse events.

Serious adverse events

A "serious adverse event" is defined as an event, regardless of the severity of symptoms, that is judged to be serious or not according to the following criteria

1. death
2. Items that may lead to death
3. Those that require hospitalization or extended hospitalization for treatment.
4. obstacle
5. Items that may lead to disability
6. Serious according to 1~5
7. Congenital diseases or anomalies in later generations

10.3 Assessment of adverse events

Adverse events observed during the conduct of clinical research will be evaluated according to the schedule specified in "Observation and Examination Items and Schedule".

The severity of adverse events will be determined based on the NCI-Common Terminology Criteria for Adverse Events (NCI-CTC-AE Ver4.0 Japanese version).

10.4 Anticipated adverse events and responses

10.4.1 Anticipated adverse events

In a previous study of 24 patients who received activated NK cell therapy to prevent recurrence of hepatocellular carcinoma after living donor liver transplantation, the adverse events that occurred were as follows, all of which were resolved by follow-up or drug administration.

1. Infusion reaction: fever, malaise (3/24 cases, 12.5%), chills, myalgia, diarrhea (2/24 cases, 8.4%), nausea, skin rash, sinus tachycardia (1/24 cases, 4.2%)
2. GVHD (0/24 cases, 0%)
3. Infectious diseases (7/24 cases, 29.2%)
4. Contamination of transplanted cells (including delayed onset of infection in the donor) (0/24 cases, 0%)

10.4.2 Dealing with Adverse Events

1. If necessary, treat with circulatory and respiratory support and steroids.
2. Take necessary measures according to clinical symptoms.
3. Treat with antibiotics, antivirals, and antifungals as needed.

4. If positive results are obtained in the sterility and endotoxin tests on culture test samples, administration should be discontinued immediately, and contaminated cells should be disposed of appropriately according to the waste disposal procedure. If the drug has already been administered, treat with antibiotics, antivirals, or antifungals as necessary.

10.5 Response to outbreaks of disease, etc.

10.5.1 Measures to be taken in case of outbreak of disease, etc.

Reporting Procedure

Based on the "Act on Securing Safety of Regenerative Medicine, etc.

Principal Investigator

Events and information that are judged to be serious by the Hiroshima University Regenerative Medicine Committee must be promptly reported to the Hiroshima University Hospital administration.

In addition, this clinical research may be discontinued or provisional measures may be taken, if necessary, before receiving instructions from the Hiroshima University Hospital administration.

Head of the research institution

1. When a serious situation is reported by a principal investigator, the occurrence and details of the situation shall be promptly reported to the Minister of Health, Labor and Welfare, the opinions of the Hiroshima University Regenerative Medicine Committee shall be promptly obtained on the policy for dealing with the situation, including analysis of the cause, and instructions shall be given to said principal investigator to stop the research or take other necessary measures. The committee shall promptly hear the opinion of the Hiroshima University Committee on Frontier Medical Sciences regarding the policy for dealing with the research, including the analysis of the research, and instruct the principal investigator of the research to stop the research or take other necessary measures. If necessary, the head of the research institution may instruct the person responsible for the research to suspend the research or to take temporary measures before hearing the opinion of the Hiroshima University Regenerative Medicine Committee.
2. The head of the research institution shall instruct the principal investigator to take the necessary measures listed in 1) above, and then report to the Minister of Health, Labor and Welfare the opinion of the Hiroshima University

Regenerative Medicine Committee, the results of the analysis of the cause, and the details of the measures instructed to the principal investigator.

3. 2), the results shall be reported to the Minister of Health, Labour and Welfare after discontinuation or other necessary measures have been taken.

10.5.2 (Reporting of Diseases, etc. to the Accredited Committee for Regenerative Medicine, etc.)

In the event that any of the following events occurs, a report in accordance with Attachment Form 1 shall be submitted to the Accredited Committee for Rehabilitation Medicine, etc. on the relevant event within the period specified in each case.

(1) Outbreak of any of the following diseases, etc., which are suspected to be caused by the provision of said regenerative medicine, etc. or infectious diseases suspected to be caused by the provision of said regenerative medicine, etc.: 7 days

(a) Death

(a) Cases that may lead to death

(2) Outbreak of the following diseases, etc., which are suspected to be caused by the provision of said regenerative medicine, etc. or infectious diseases suspected to be caused by the provision of said regenerative medicine, etc.: 15 days

(a) Cases requiring hospitalization or extension of hospital stay at a medical institution for treatment

(a) Disability

(c) Cases that may lead to disability

(d) Cases that are serious (equivalent to a through c above)

(e) Congenital diseases or abnormalities in later generations

(3) Outbreak of disease, etc. caused by infectious diseases suspected to be caused by the provision of regenerative medicine, etc. or suspected to be caused by the provision of said regenerative medicine, etc. (excluding those listed in (1) and (2)) (3) Occurrence of diseases, etc. caused by infectious diseases suspected or alleged to be caused by the provision of regenerative medicine, etc. (excluding those listed in (1) and (2))

10.5.3 (Reporting of illness, etc. to the Minister of Health, Labor and Welfare)

In the event of the occurrence of any of the following matters, a report in accordance with Attachment Form 2 shall be submitted to the Minister of Health, Labor and Welfare on said matters within the period specified for each.

(1) Outbreak of any of the following diseases, etc., which are suspected to be caused by the provision of said regenerative medicine, etc. or infectious diseases suspected to be caused by the provision of said regenerative medicine, etc.: 7 days

(a) Death

(a) Cases that may lead to death

(2) Outbreak of any of the following diseases, etc., which are suspected to be caused by the provision of said regenerative medicine, etc. or infectious diseases suspected to be caused by the provision of said regenerative medicine, etc. 15 days

(a) Cases requiring admission to a medical institution for treatment or extension of hospital stay

(a) Disability

(c) Cases that may lead to disability

(d) Cases that are serious

(e) Subjects with congenital diseases or abnormalities in later generations.

Reporting Procedure

Based on the "Act on Securing Safety of Regenerative Medicine, etc.

Principal Investigator

Events and information that are judged to be serious by the Hiroshima University Regenerative Medicine Committee must be promptly reported to the Hiroshima University Hospital administration.

In addition, this clinical research may be discontinued or provisional measures may be taken, if necessary, before receiving instructions from the Hiroshima University Hospital administration.

Head of the research institution

4. When a serious situation is reported by a principal investigator, the occurrence and details of the situation shall be promptly reported to the Minister of Health, Labor and Welfare, the opinions of the Hiroshima University Regenerative Medicine Committee shall be promptly obtained on the policy for dealing with the situation, including analysis of the cause, and instructions shall be given to

said principal investigator to stop the research or take other necessary measures. The committee shall promptly hear the opinion of the Hiroshima University Committee on Frontier Medical Sciences regarding the policy for dealing with the research, including the analysis of the research, and instruct the principal investigator of the research to stop the research or take other necessary measures. If necessary, the head of the research institution may instruct the principal investigator to suspend the research or take other provisional measures before hearing the opinion of the Hiroshima University Regenerative Medicine Committee.

5. The head of the research institution shall instruct the principal investigator to take the necessary measures listed in 1) above, and then report to the Minister of Health, Labor and Welfare the opinion of the Hiroshima University Regenerative Medicine Committee, the results of the analysis of the cause, and the details of the measures instructed to the principal investigator.
6. 2), the results shall be reported to the Minister of Health, Labour and Welfare after discontinuation or other necessary measures have been taken.

11 Criteria and procedures for discontinuation of clinical research for each subject

11.1 Criteria for discontinuation of clinical research for each subject

The principal investigator or sub-investigator shall discontinue or suspend clinical research on the subject in the following cases

1. If a positive test is found in the infectious disease test of culture test material.
2. In addition to 1) above, if protocol treatment is no longer possible
3. When a subject requests withdrawal of consent for participation in clinical research.
4. When the occurrence of an adverse event is recognized and the principal investigator judges that it is difficult to continue the clinical research on the subject concerned.
5. If it is discovered after enrollment but before the start of protocol treatment that the subject did not meet the eligibility criteria
6. In other cases, when the principal investigator or sub-investigator deems it appropriate to discontinue the clinical research.

11.2 Procedures for discontinuing clinical research for each subject

The principal investigator shall promptly explain to the subject concerned that the clinical research is to be discontinued, and shall provide appropriate medical care and take other necessary measures. The reason for discontinuation and the date of discontinuation shall be stated in the case report form. If clinical research is discontinued after protocol treatment has been completed, observation and examination of the subject shall be continued as much as possible.

12 Compliance with the clinical research protocol and management of non-compliance

12.1 Compliance with the clinical research protocol

This clinical research will be conducted in compliance with this protocol, except in cases where it is medically unavoidable, such as to avoid urgent danger to subjects.

12.2 Management of non-conformities in the implementation plan

The principal investigator or sub-investigator may deviate from this protocol if there are unavoidable medical circumstances, such as the need to avoid immediate danger to subjects. In such cases, the principal investigator shall report the details of the deviation or change and the reason for the deviation or change to the Hiroshima University Regenerative Medicine Committee via the head of the research institution as soon as possible. In addition, the principal investigator or sub-investigator shall record all deviations from this protocol, regardless of the reason for the deviation.

If it is appropriate to change or revise the protocol, the draft shall be submitted to the Hiroshima University Regenerative Medicine Committee for approval via the head of the research institution. When changes are made, the ethical, scientific, and medical appropriateness of the changes will be thoroughly reviewed. The procedures for deviations and changes will be in accordance with the established procedures.

(2) When the rehabilitation medical care, etc. is found to be in a state that does not conform to the rehabilitation medical care provision plan, the fact shall be promptly reported to the administrator of the medical institution where the rehabilitation medical care, etc. is provided. In addition, when a particularly serious

non-conformity is found, the opinion of the authorized rehabilitation medical care commission shall be promptly heard.

13 Termination or discontinuation of the entire clinical study

13.1 Completion of clinical research

If the enrollment of the target cases is completed within the scheduled period, the date of the end of the observation period for the last enrolled case shall be the date of the end of the clinical research implementation period, and the following procedures shall be followed.

13.1.1 Procedures for Termination of Research in Clinical Research

The principal investigator will report the completion of the clinical research implementation period to the chief statistician, the Hiroshima University Committee on Regenerative Medicine, the Hiroshima University Hospital administration, and relevant departments.

In addition, the principal investigator must prepare a summary report promptly after the completion of the clinical research and submit it to the administrator of Hiroshima University Hospital. In addition, the administrator of Hiroshima University Hospital must promptly submit a copy of the received summary report to the Minister of Health, Labor and Welfare.

13.2 Criteria and procedures for interruption or discontinuation of the entire clinical study

13.2.1 Criteria for interruption or discontinuation of clinical research as a whole

The principal investigator will consider whether or not to continue the clinical research if any of the following applies

1. When significant information on the quality, safety, or efficacy of NK cell preparations is obtained
2. When it is judged difficult to complete the clinical research due to reasons such as delay in case enrollment or frequent deviation from the protocol.
3. If the safety of the protocol treatment is judged to be problematic based on the evaluation of the Hiroshima University Committee for Regenerative Medicine, etc.
4. If, as a result of evaluation of relevant information obtained from sources other than this clinical study, such as papers and conference presentations, it is

determined that there is a problem with the safety of the protocol treatment, or that the continuation of the clinical study is no longer meaningful.

13.2.2 Procedures for suspending or discontinuing the entire clinical study

The principal investigator shall promptly report to the Hiroshima University Regenerative Medicine Committee after discontinuing the clinical research.

In addition, the principal investigator must prepare a summary report promptly after discontinuation of the clinical research and submit it to the administrator of Hiroshima University Hospital. In addition, the administrator of Hiroshima University Hospital must promptly submit a copy of the received summary report to the Minister of Health, Labor and Welfare.

14 case report

14.1 data management

Data management will be outsourced to the Data Science Division (hereinafter referred to as "Data Center") of Hiroshima University Hospital's Center for Comprehensive Medical Care Research Promotion, in accordance with the Standard Operating Procedures for Clinical Research Quality Management (Monitoring and Data Management).

14.2 Case report writing and data collection

1. The principal investigator or sub-investigator shall prepare a case report form for each enrolled subject, submit it to the data center with his/her name, seal, or signature, and retain a copy of the report.
2. When research collaborators assist in the preparation of case report forms, they should only transcribe from the source documents to the extent that it does not involve medical judgment, under the supervision of the principal investigator or research subcontractor.

14.3 Notes on the Case Report Form

1. Use a black ballpoint pen or a pen with black ink.
2. The following is a summary of the results.
3. If no data is available due to lack of observation or inspection, put a shaded line (/) in the entry field.
4. Check for consistency with the source documents.

14.4 Changes or modifications to the case report form

1. When changing or modifying a case report, cross out the changed or modified part with a double line (=), write the correct information near the changed or modified part, add the date of the change or modification, and seal or sign the document. Do not use correction fluid, sand eraser, etc. to obscure the original description.
2. In the case of changes or modifications to important information [consent, endpoint assessment (name of adverse event, date of occurrence, grade, severity, treatment, date of outcome/determination, causal relationship, comments)], the reason for the change or modification should be stated in addition to the date of the change or modification, and the change or modification should be stamped or signed.
3. Changes or corrections to the case report form after submission to the data center should be made via the DCF (Data Clarification Form) designated by the data center.

14.5 Review of case reports

1. When a case report form is prepared by a research subcontractor, the principal investigator should inspect the contents of the case report form, confirm that there are no problems, and then write his/her name, seal, or signature on the form before submitting it to the data center.
2. Principal investigators will ensure that case report forms submitted to the data center are accurate, complete, legible, and submitted at the appropriate time, and that subject identification codes and registration numbers are used to identify subjects.

14.6 Submission of case reports

The principal investigator, subcontractors, and research collaborators shall prepare a case report form, sign it with their names and seals, and submit it to the data center within six weeks after the completion of the final observation of each volume in the case or the discontinuation of clinical research, and keep a copy of the report.

15 Statistical Analysis

15.1 Statistical Analysis Method

As a rule, for items observed as continuous values, the number of examples, mean (median), standard deviation (quartiles), and range (minimum-maximum) are calculated as summary statistics. For items observed as discrete values, the number of examples in each category and their proportions are calculated as summary statistics.

Statistical tests for the main analysis will be performed at the 5% significance level (two-sided).

15.2 Definition of the population for analysis

15.2.1 Efficacy analysis population

Subjects who are enrolled in this clinical study and underwent living partial liver transplantation and NK cells were administered on postoperative day 3 will be the target population for efficacy analysis (the largest analysis set Full Analysis Set: FAS).

15.2.2 Population for safety analysis

Subjects who are enrolled in this clinical study, underwent living partial liver transplantation, and received NK cells on postoperative day 3 will be the population for safety analysis.

15.3 Handling of missing values

Withdrawals due to discontinuation or deviations during the course of the study will be reported to the

- ✓ The data observed immediately before the subject's discontinuation or dropout will be analyzed as the last observation data (Last Observation Carried Forward: LOCF).
- ✓ Analysis based on observed values without supplementing missing data (Observed Case: OC)
- ✓ Discontinuation and dropout cases will be analyzed as treatment failure cases (bacteremia cases/ Failure).

The robustness of the results of the analysis by the method of the primary endpoint will be confirmed. The primary endpoint will be analyzed as a failure (failure).

15.4 Subject background and baseline characteristics

For each of the safety and efficacy analysis populations, the subject background and baseline characteristics are summarized using summary statistics and listed in the table. The data used as the baseline is the preoperative data, and if there is more than one data, the most recent data on the date of surgery will be used.

In addition, the number and percentage of subjects who completed this clinical study and the number of subjects who discontinued should be summarized for each discontinuation period, and the reasons for discontinuation should also be summarized.

15.5 Efficacy analysis

15.5.1 Primary endpoints

Incidence of bacteremia within 1 month after living donor liver transplantation

[Main analysis]

The incidence of bacteremia within 1 month after living donor liver transplantation and its 95% confidence interval will be calculated for FAS. In addition, the following statistical hypothesis test will be performed at a significance level of 5% (two-tailed) by chi-square test using the 30% incidence of bacteremia with non-NK therapy observed in previous studies as a threshold.

The null hypothesis: "The incidence of bacteremia p in NK therapy is equal to a threshold of 30% ($p = 0.3$)."

Counter-hypothesis: "The incidence of bacteremia p in NK therapy is less than the threshold of 30% ($p < 0.3$)"

15.5.2 Secondary endpoints

① Overall survival time

FAS will be observed from the date of surgery to the date of death (regardless of the cause of death) or the date of confirmed survival, and the survival curve for overall survival will be estimated using the Kaplan-Meier method, and the median overall survival and its 95% confidence interval will be calculated. The median overall survival and its 95% confidence interval will be calculated. The survival rates at 6 months, 1 year and 3 years and its 95% confidence interval will be calculated. The Greenwood method will be used to calculate the confidence intervals.

② Effect on immune response (evaluation of donor-specific immune responsiveness by CFSE-MLR, detection of DSA, incidence of rejection, evaluation of NK cell activity in recipient peripheral blood).
For CFSE-MLR and evaluation of recipient peripheral blood NK cell activity for FAS, summary statistics and changes from baseline (mean (median), standard deviation (quartiles)) and their 95% confidence intervals will be calculated for each observation time point. For detection of DSA and incidence of rejection, the number of cases in each category and its percentage will be calculated.

③ Hepatocellular carcinoma recurrence / De novo carcinogenesis
We will observe whether hepatocellular carcinoma (HCC) recurs in FAS and the date of recurrence, and calculate the HCC recurrence rate and its 95% confidence interval, and the mean time to recurrence and its 95% confidence interval. De novo carcinogenesis will be analyzed in the same way.

④ Incidence of infectious diseases
The percentage of subjects who developed bacterial, cytomegalovirus, and fungal infections and their 95% confidence intervals will be calculated. The incidence of infectious diseases by genetic polymorphism will be calculated, and comparisons between genetic polymorphisms will be made using Fisher's direct probability method at a significance level of 5% (two-sided).

15.6 Safety analysis

adverse event

The number of cases, number of incidences, and percentage of incidences of adverse events will be tabulated by event and by severity. The number of cases, number of incidences, and percentage of incidences of adverse events by event type and severity will also be tabulated.

vital signs

Summary statistics of the vital signs are obtained for the change at each time point from the observed values at the scheduled postoperative measurement time points and the observed values at the preoperative examination (baseline) and are listed in the table.

clinical laboratory test results

Summary statistics of the clinical laboratory data (hematological and biochemical tests) will be obtained and listed for the amount of change at each time point from the observed values at the scheduled postoperative measurement time points and the observed values at the preoperative examination (baseline). For qualitative clinical laboratory data, the frequency of pairs of laboratory category values at baseline and each measurement time point is tabulated in the form of a contingency table.

15.7 Procedure for changing the analysis plan

If changes or additions to the analysis plan occur after the start of the study, the appropriateness of the changes or additions and their impact on the evaluation of the study should be considered, and the circumstances leading to the changes or additions to the analysis plan should be described in the summary report. For major analyses and analyses of important endpoints, changes to the protocol should be made as well.

16 Quality control for clinical research

16.1 Quality Control

Principal investigators, subcontractors, and research collaborators will conduct clinical research in compliance with the Act on Securing the Safety of Regenerative Medicine, etc., the Ethical Guidelines for Medical Research Involving Human Subjects, and this protocol. In addition, Principal Investigators, Research Associates, and Research Collaborators will conduct clinical research in accordance with their respective procedures for conducting clinical research.

16.2 monitoring

Since this clinical research falls under the category of research that involves invasion and intervention, the principal investigator will entrust monitoring to the Hiroshima University Hospital Center for Comprehensive Medical Care and Research, and will conduct monitoring in accordance with the standard procedures for quality control of clinical research owned by the center.

The person in charge of monitoring will conduct monitoring at a separately designated time to confirm that the research is being conducted appropriately in compliance with the Act on Securing the Safety of Regenerative Medicine, etc., the

Ethical Guidelines for Medical Research Involving Human Subjects, and this protocol, and that the reliability of the data is sufficiently secured. The results of the monitoring shall be reported to the principal investigator.

16.3 data management

The principal investigator or sub-investigator should describe and prepare the data obtained from the subject in the case report form without delay. The case report form, the donor liver-derived activated lymphocyte production instruction form, the consent form, and a copy of the test results will be filed for each case and stored in a locked archive in the Department of Gastroenterology and Transplantation, Hiroshima University Graduate School of Medicine. The prepared case report form will be submitted to the Data Center without delay, and the Data Center will manage the data in accordance with the Standard Procedure for Quality Control of Clinical Research owned by the Hiroshima University Hospital Center for Comprehensive Medical Care Research.

16.4 Effectiveness and Safety Evaluation Committee

16.4.1 Details of deliberations by the Effectiveness and Safety Evaluation Committee

The Efficacy and Safety Evaluation Committee shall consist of three physicians, excluding the subcontractors, and shall deliberate on the following matters

- Contents of the annual implementation status report
- Confirmation of the efficacy and safety of the NK cells and whether the study should be continued at an interim total to be conducted at the end of the observation period of one month after the 15th surgery in which NK cells were administered.
- Assessment of serious adverse events
- Other matters as deemed necessary by the principal investigator or the Efficacy and Safety Evaluation Committee.

16.4.2 Recommendations by the Effectiveness and Safety Evaluation Committee

When items to be recommended are proposed by the Effectiveness and Safety Evaluation Committee, the contents and reasons will be recommended to the principal investigator.

16.4.3 Audit

No audit will be conducted in this study.

16.5 Education and training of researchers

The principal investigators will provide education and training opportunities for researchers participating in this research before the start of the research and at least once per year after the start of the research to deepen their understanding of the following examples.

1. Ministry of Health, Labour and Welfare, "Act on Securing the Safety of Regenerative Medicine, etc.
2. Knowledge about specified cell processing products, etc. (including ethical concepts)
3. Knowledge and skills related to the safe handling of prepared processed cell culture products, etc.
4. Knowledge and skills related to facilities and equipment
5. Knowledge and skills related to the safety of the preparation process
6. Knowledge and skills regarding measures to be taken in the event of an accident
7. In accordance with the "Ethical Guidelines for Medical Research Involving Human Subjects," research ethics and the knowledge and skills necessary to conduct research

17 Committee for Regenerative Medicine, Hiroshima University

17.1 Deliberation by the Hiroshima University Committee for Regenerative Medicine

The Chairperson of the Hiroshima University Committee for Regenerative Medicine will deliberate on the progress report of clinical research once a year. The Chair of the Hiroshima University Committee for Regenerative Medicine may also deliberate at the request of the principal investigator or when he/she deems it necessary. The format of the deliberations will be decided by the chairperson according to the importance of the deliberations, such as convening a committee meeting, rotating the deliberations, or listening to opinions by telephone or e-mail.

17.2 Deliberations of the Committee for Regenerative Medicine, Hiroshima University

The Hiroshima University Committee for Regenerative Medicine will deliberate on the following matters.

1. Progress check
2. Whether or not to continue the research due to the occurrence of serious adverse events
3. Other matters deemed necessary by the chairperson

17.3 Recommendation by the Hiroshima University Committee for Regenerative Medicine

If a recommendation to be made is proposed, the content and reasons will be recommended to the principal investigator with the agreement of all deliberative committee members. In cases where the consensus of all committee members cannot be reached, the chairperson of the committee will summarize the opinions and make recommendations, including minority opinions.

(3) The administrator of the provider institution shall report to the committee on changes to the rehabilitation medical care provision plan and other measures taken after receiving opinions from the rehabilitation medical care commission.

18 Ethical Conduct of Clinical Research

This clinical research will be conducted with attention to the ethical principles based on the Declaration of Helsinki, and in compliance with the "Act on Securing the Safety of Regenerative Medicine, etc.", the "Ethical Guidelines for Medical Research Involving Human Subjects", and this protocol.

18.1 Committee for Regenerative Medicine, Hiroshima University

The Hiroshima University Committee for Regenerative Medicine, in consultation with the Hiroshima University Hospital administration, will deliberate on the implementation and continuation of clinical research from the perspective of ethical, scientific and medical validity, based on the contents of the clinical research protocol, explanatory documents (for patients and liver donors) and case report forms.

18.2 Progress report on clinical research

(2) With regard to the status of provision of regenerative medicine, etc., the following matters shall be reported to the Hiroshima University Regenerative Medicine Committee and the Minister of Health, Labour and Welfare.

(1) Number of persons who have received said regenerative medicine, etc.

(2) Occurrence and subsequent progress of the disease, etc. pertaining to the relevant regenerative medicine, etc.

(3) Evaluation of the safety and scientific validity of said regenerative medicine, etc.

(4) In cases where the provision of said regenerative medicine, etc. has been terminated, the date of termination.

This report shall be made every year starting from the date of submission of the plan for provision of regenerative medicine, etc. to the Minister of Health, Labour and Welfare, within 90 days after the expiration of said period. Reports to the Hiroshima University Committee for Biomedical Research and Innovation shall be made by submitting a report in accordance with Attachment Form 3, and reports to the Minister of Health, Labour and Welfare shall be made by submitting a report in accordance with Attachment Form 4.

18.3 Matters concerning the protection of human rights and personal information of subjects

18.3.1 Human rights of subjects

This research will be conducted in compliance with the "Declaration of Helsinki" by the World Medical Association, the "Act on Securing the Safety of Regenerative Medicine, etc.", and the "Ethical Guidelines for Medical Research Involving Human Subjects". From the perspective of protecting the human rights of subjects, the principal investigator and sub-investigators will carefully consider the appropriateness of requesting subjects to participate in this clinical research, giving due consideration to their health conditions, symptoms, age, gender, and capacity to consent. Particular care should be taken when subjects are socially vulnerable.

18.3.2 Protection of Personal Information

After obtaining the subject's consent, all handling of the case, including data management and manufacturing management, will be managed using a linkable anonymized subject identification code or registration number, and the comparison table of the anonymized code and the name and the consent form with the name will be kept strictly in a lockable document storage room. In addition, sufficient

consideration will be given to the protection of subjects' personal information, such as ensuring that the names of subjects are not directly disclosed in public announcements.

Personal Information Manager:.

Affiliation Graduate School of Medical Sciences, Hiroshima University Title Professor Name Kazuteru Tanabe

19 Preservation of records, etc.

19.1 Preservation of samples

Cells and other materials collected during clinical research will be used for basic experiments and may be used for other research, so they will be stored at the Department of Digestive and Transplant Surgery, Hiroshima University Graduate School of Medical Sciences until they are used up. The samples will be coded and managed in an anonymized manner, but since there is a possibility of examining the relationship with prognosis, etc., the personally identifiable information manager will manage the samples in a consolidated anonymized manner. If the samples are to be used for other research in the future, they will again undergo ethical review by the Hiroshima University Committee for Regenerative Medicine. A portion of the samples and administered cells will be cryopreserved for 10 years to search for causes of adverse events in the future. Samples should be disposed of as infectious medical waste after autoclaving for at least 10 years after completion of the study.

19.2 Preservation of materials

Documents and records related to clinical research, including case reports, will be stored at the Department of Gastroenterology and Transplantation Surgery, Hiroshima University Graduate School of Medical Sciences.

(2) A record of the receipt of regenerative medicine shall be prepared for each person who has received regenerative medicine, etc. with regard to the following matters

(1) Address, name, gender, and date of birth of the person who received the regenerative medicine, etc.

(2) Disease name and main symptoms

(3) Type of specified cell processing product or regenerative medicine product used, method of administration, and other details and evaluation of regenerative medicine, etc.

(4) Information on cells to be used for regenerative medicine, etc. (such as the place where said cells were collected, the date, the results of confirmation of the eligibility of said cell donor, and the results of tests that confirmed the appropriateness of said cells)

(5) In the case of consignment of the manufacture of specified cell processing products, the consignee and the details of the consignment

(6) Date when the regenerative medicine, etc. was performed

(7) Name of the doctor who performed the regenerative medicine, etc.

Documents and records pertaining to clinical research (the provisional plan for regenerative medicine, documents pertaining to consent, and the summary of specified cell processing products) shall be preserved for at least 30 years from the date of discontinuation or completion of clinical research and submission of the summary report. If there are any changes or amendments to the protocol or case report form, the history of such changes or amendments shall be stored appropriately.

The person responsible for the management of records, etc.

Affiliation Graduate School of Medical Sciences, Hiroshima University Title Professor Name Kazuteru Tanabe

20 Preparation of clinical research summary report

After discontinuation or termination of the clinical research, the principal investigator shall promptly prepare a clinical research summary report.

21 Obtaining information on subjects after clinical research is completed

Regular outpatient visits will be encouraged even after the completion of the study. Regular outpatient consultations will be conducted to evaluate the presence of complications and efficacy. All serious adverse events resulting from the provision of regenerative medicine, etc. will be reported. Patients will be asked to continue outpatient visits for three years after the end of treatment.

Data obtained from regular outpatient clinics after the completion of clinical research will not be included in the analysis. Any outbreak of disease, etc. will be reported to the Hiroshima University Committee for Regenerative Medicine.

22 Compensation for clinical research expenses and health damage

22.1 Sources of funding for clinical research and conflicts of interest

Research expenses (subsidy for operation)

This clinical trial will be funded by an operating grant distributed to the Department of Gastrointestinal and Transplantation Surgery at Hiroshima University. Any conflicts of interest have been reviewed by the Clinical Research Conflict of Interest Management Committee of Hiroshima University, and there are no conflicts of interest to be disclosed.

22.2 Cost sharing for clinical research

The cost of conducting clinical research on cases enrolled at Hiroshima University Hospital in this clinical study will be covered by the research fund of the Department of Digestive and Transplant Surgery, Hiroshima University Graduate School of Medical Sciences.

22.3 Compensation for health damage, etc.

Recipients of regenerative medicine: In the event of an adverse event arising from this clinical research, the principal investigator will endeavor to recover the subject by taking the best medical measures. In addition, this clinical research is covered by the clinical research compensation insurance, and in the event of death or serious adverse events caused by the implementation of this research without negligence, the clinical research insurance for regenerative medicine will compensate for the damage, unless the damage is caused by reasons attributable to the subject or other exempt reasons. In the event of serious adverse events or other health hazards, they will be compensated by the clinical research insurance for regenerative medicine, etc., unless the damage is caused by reasons attributable to the subject or other exempt reasons.

Cell donors: The cells used for the treatment will be collected cleanly after the liver is removed from the body during a normal liver transplant donor surgery. Therefore, cell donors will not suffer any health hazard due to participation in this

treatment. If any health problems related to the liver transplant donor surgery occur, they will be treated by medical insurance.

23 Arrangements regarding the attribution of clinical research results and the registration and publication of clinical research results

The intellectual property rights arising from this clinical research shall belong to the researcher.

Prior to the implementation of this clinical study, it will be pre-registered in the database established by the University Hospital Medical Information Network Research (UMIN) Center. The results of this clinical study will be summarized in a summary report. The results of this clinical study will be summarized in a summary report and, if necessary, published as a paper or conference presentation. When the results are published, sufficient consideration will be given to the protection of the subjects' personal information, such as ensuring that the subjects' names are not directly published.

Clinical Research Implementation System

23.1 Principal Investigator

Name	Institution name, department/affiliation, position, phone number	Role in clinical research
Hideki Ohdan	Graduate School of Medical Sciences, Hiroshima University<br>Professor, Department of Gastrointestinal and Transplantation Surgery<br>[redacted]	Principal Investigator<br>Patient selection, surgery, and evaluation

23.2 member (of a research project)

Name	Institution name, department/affiliation, position, phone number	Role in clinical research
Masahiro Ohira	Future Medical Center, Hiroshima University Hospital<br>Assistant Professor<br>[redacted]	Patient selection, surgery, and evaluation<br>Clinical Research Manager

Yuka Tanaka	Graduate School of Medical Sciences, Hiroshima University<br>Associate Professor, Department of Gastrointestinal and Transplantation Surgery<br>[redacted]	Immunological evaluation
Junko Tanaka	Graduate School of Medical Sciences, Hiroshima University<br>Department of Integrative Health Sciences<br>Professor, Department of Epidemiology and Disease Control<br>[redacted]	Statistical Analysis
Tsuyoshi Kobayashi	Hiroshima University Graduate School of Medical Sciences<br>Lecturer, Department of Gastroenterological Surgery and Transplant Surgery, Hiroshima University Graduate School of Medical Sciences<br>[redacted]	Patient selection, surgery, and evaluation
Kentaro Ide	Department of Gastroenterological Surgery and Transplant Surgery, Hiroshima University Hospital<br>Assistant Professor<br>[redacted]	Patient selection, surgery, and evaluation
Hiroyuki Tahara	Department of Gastroenterology and Transplant Surgery, Hiroshima University Hospital<br>assistant teacher<br>[redacted]	Patient selection, surgery, and evaluation

23.3 contact address

Name	Name, affiliation, representative (title and name), [redacted]
Medical institutions<br>Principal Investigator	Name: Hiroshima University Graduate School of Biomedical Sciences<br>Affiliation: Department of Gastrointestinal and Transplantation Surgery<br>Representative: Professor Hideki Ohdan<br>[redacted]
Research Secretariat	Name: Department of Gastroenterology and Transplantation, Hiroshima University Graduate School of Medical Sciences<br>Contact person: Assistant Professor Masahiro Ohira<br>[redacted]
Data Center<br>monitoring	Name: Division of Data Science, Center for Promotion of Comprehensive Medical Research, Hiroshima University Hospital<br>[redacted]
Consultation Service	Name: Future Medical Center, Hiroshima University Hospital<br>Contact person: Masahiro Ohira<br>[redacted]

24 literature

Colonna, J. O., 2nd, Winston, D. J., Brill, J. E., et al. (1988). Infectious complications in liver transplantation. *Arch Surg, 123*(3), 360.

Fishman, J. A., & Rubin, R. H. (1998). Infection in organ-transplant recipients. *N Engl J Med, 338*(24), 1741.

Horan, T. C., Andrus, M., & Dudeck, M. A. (2008). CDC/NHSN surveillance definition of health care-associated infections and criteria for specific types of infections in the acute care setting. *Infect Control, 36*(5), 309.

Ishiyama, K., Ohdan, H., Ohira, M., et al. (2006). Difference in cytotoxicity against hepatocellular carcinoma between liver and periphery natural killer cells in humans. *Hepatology, 43*(2), 362.

Kawecki, D., Chmura, A., Pacholczyk, M., et al. (2009). Bacterial infections in the early period after liver transplantation: etiological agents and their susceptibility. *Med Sci Monit, 15*(12), CR628.

Kim, S. I., Kim, Y. J., Jun, Y. H., et al. (2009). Epidemiology and risk factors for bacteremia in 144 consecutive living-donor liver transplant recipients. *Yonsei Med J, 50*(1), 112.

Ochi, M., Ohdan, H., Mitsuta, H., et al. (2004). Liver NK cells expressing TRAIL are toxic against self hepatocytes in mice. *Hepatology, 39*(5), 1321.

Ohira, M., Ishiyama, K., Tanaka, Y., et al. (2009). Adoptive immunotherapy with liver allograft-derived lymphocytes induces anti-HCV activity after liver transplantation in humans and humanized *J Clin Invest, 119*(11), 3226.

Ohira, M., Nishida, S., Tryphonopoulos, P., et al. (2012). Clinical-scale isolation of interleukin-2-stimulated liver natural killer cells for treatment of liver transplantation with hepatocellular *Cell Transplant, 21*(7), 1397.

Ohira, M., Ohdan, H., Mitsuta, H., et al. (2006). Adoptive transfer of TRAIL-expressing natural killer cells prevents recurrence of hepatocellular carcinoma after partial hepatectomy. *Transplantation, 82*(12), 1712.

Soave, R. (2001). Prophylaxis strategies for solid-organ transplantation. *Clin Infect Dis, 33 Suppl 1*, S26.

Tashiro, H., Ishiyama, K., Ohira, M., et al. (2011). Impact of adjuvant immunotherapy using liver allograft-derived lymphocytes on bacteremia in living-donor liver transplantation. *Transplantation, Transplantation, 92*(5), 575.

Winston, D. J., Emmanouilides, C., & Busuttil, R. W. (1995). Infections in liver transplant recipients. *Clin Infect Dis, 21*(5), 1077.
