# Supplementary material for: A phase I/II study of adoptive immunotherapy using donor liver graft-derived NK cell-enriched immune cells to prevent severe infection after liver transplantation
Source: PLoS One. 2025 Jan 16;20(1):e0313102. doi: 10.1371/journal.pone.0313102 (PMC11737672; doi:10.1371/journal.pone.0313102)
Supplement: S2 File — (PDF) [file pone.0313102.s002.pdf]

臨床研究実施計画書

感染症予防を目的とした肝臓移植における ドナー肝臓由来活性化ナチュラルキラー（NK)細胞を用いた術後免疫賦活療法の臨床応用

研究責任者　大段　秀樹

所属・役職　　広島大学大学院医系科学研究科　消化器・移植外科学　教授

作成日：令和元年 5 月 20 日　第　8　版

承認年月日	
承 認 者 署 名	

制定・改訂履歴表

版数	制定・改訂年月日	理由	承認	確認	作成
1	2015.8.3	新規制定			
2	2015.9.14	委員会意見による改訂			
3	2015.11.20	委員会意見による改訂			
4	2016.2.16	分担研究者変更による改訂			
5	2017.3.13	分担研究者変更による改訂			
6	2018.10.1	研究期間延長による改訂			
7	2018.10.26	委員会意見による改訂			
8	2019.5.20	再生医療法省令変更、IL-2 製剤変更による改訂			

内容

0 概要 8
シェーマ 12
観察・検査スケジュール 13
語句の定義 15
背景（平易な表現）： 16
内容（平易な表現）： 16
1 研究目的 17
2 経緯 17
2.1 対象疾患 17
2.1.1 概念・定義・病因・病態 17
2.1.2 疫学 17
2.1.3 標準治療と予後 18
2.1.4 対象疾患の設定根拠 18
2.2 特定細胞加工物名及びその概要 18
2.2.1 特定細胞加工物名 18
2.2.2 試験物の概要 18
2.2.3 これまでの前臨床試験、臨床研究及び臨床試験の結果の要約 19
2.2.4 臨床研究実施が可能であると判断した理由 22
2.3 登録患者の予想される利益と不利益 22
2.3.1 予想される利益 22
2.3.2 予想される不利益 23
2.4 本臨床研究の意義 23
3 対象疾患と適格基準 23
3.1 対象疾患 23
3.2 選択基準 23
3.3 除外基準 24
4 同意取得 25
4.1 同意説明文書及び同意書の作成 25
4.2 同意説明文書及び同意書の改訂 25
4.3 同意説明及び同意取得の時期及び方法 25
5 登録 26
5.1 被験者登録 26
6 研究で用いる特定細胞加工物 27

6.1 特定細胞加工物名 27
6.2 成分・構造・特性・製造方法 27
6.2.1 規格 27
6.2.2 製造方法 27
6.3 容器・包装・保存条件 28
6.4 交付・搬送 28
6.5 管理・保管 28
7 試験デザイン 29
7.1 試験デザイン 29
7.2 目標登録被験者数・被験者登録期間 29
7.3 目標登録被験者数の集積可能性 30
7.4 プロトコル治療計画 30
7.4.1 プロトコル治療の定義 30
7.4.2 方法 30
7.4.3 併用治療 31
7.4.4 感染症治療 31
7.4.5 プロトコル治療計画の設定根拠 31
7.4.6 登録被験者の研究参加期間 31
7.5 中間集計 31
8 主要評価項目及び副次評価項目 31
8.1 主要評価項目 31
8.2 副次評価項目 32
9 観察・検査項目とスケジュール 32
9.1 検査スケジュール 32
9.2 観察・検査項目 34
9.2.1 スクリーニング検査 34
9.2.2 術前検査 34
9.2.3 登録 35
9.2.4 手術日 35
9.2.5 NK 細胞投与日（術後 3 日目） 36
9.2.6 NK 細胞投与 1、2 日後（術後 4、5 日目） 37
9.2.7 NK 細胞投与 1、2 週間後 37
9.2.8 NK 細胞投与 4 週間後 37
9.2.9 NK 細胞投与 8 週間後、12 週間後 38
9.2.10 NK 細胞投与 6 カ月後、以降 6 カ月毎に 3 年間後まで 38
9.2.11 中止時 39

10 被験者の安全性の確保 39
10.1 基本的事項 39
10.2 有害事象の定義 39
10.3 有害事象の評価 40
10.4 予想される有害事象とその対応 40
10.4.1 予想される有害事象 40
10.4.2 有害事象への対処 40
10.5 疾病等発生時の対応 41
10.5.1 疾病等の発生の場合の措置 41
10.5.2 認定再生医療等委員会への疾病等の報告 41
10.5.3 厚生労働大臣への疾病等の報告 42
11 被験者毎の臨床研究中止の基準及び手順 43
11.1 被験者毎の臨床研究中止の基準 43
11.2 被験者毎の臨床研究中止の手順 43
12 臨床研究実施計画書の遵守、逸脱又は変更 43
12.1 臨床研究実施計画書の遵守 43
12.2 実施計画書からの逸脱又は変更 44
13 臨床研究全体の終了又は中止及び中断 44
13.1 臨床研究の終了 44
13.1.1 臨床研究における研究の終了の手順 44
13.2 臨床研究全体の中断・中止の基準及び手順 44
13.2.1 臨床研究全体の中断・中止基準 44
13.2.2 臨床研究全体の中断・中止の手順 45
14 症例報告書 45
14.1 データマネージメント 45
14.2 症例報告書の作成とデータの収集 45
14.3 症例報告書の記載上の注意 45
14.4 症例報告書の変更又は修正 46
14.5 症例報告書の確認 46
14.6 症例報告書の提出 46
15 統計解析 46
15.1 統計解析方法 46
15.2 解析対象集団の定義 47
15.2.1 有効性解析対象集団 47
15.2.2 安全性解析対象集団 47
15.3 欠測値の取扱い 47

15.4　被験者背景及びベースラインの特性 47
15.5　有効性の解析 47
15.5.1　主要評価項目 47
15.5.2　副次評価項目 48
15.6　安全性の解析 48
15.7　解析計画の変更手順 49
16　臨床研究の品質管理 49
16.1　品質管理 49
16.2　モニタリング 49
16.3　データ管理 50
16.4　効果安全性評価委員会 50
16.4.1　効果安全性評価委員会による審議内容 50
16.4.2　効果安全性評価委員会による勧告 50
16.4.3　監査 50
16.5　研究者の教育および研修 50
17　広島大学再生医療等委員会 51
17.1　広島大学再生医療等委員会による審議 51
17.2　広島大学再生医療等委員会の審議内容 51
17.3　広島大学再生医療等委員会による勧告 51
18　臨床研究の倫理的実施 51
18.1　広島大学再生医療等委員会 52
18.2　臨床研究の進捗報告 52
18.3　被験者の人権及び個人情報の保護に関する事項 52
18.3.1　被験者の人権 52
18.3.2　個人情報の保護 52
19　記録等の保存 53
19.1　試料の保存 53
19.2　資料の保存 53
20　臨床研究総括報告書の作成 54
21　臨床研究終了後の被験者情報の把握 54
22　臨床研究費用並びに健康被害の補償 54
22.1　臨床研究の資金源及び利益相反 54
22.2　臨床研究に関する費用負担 54
22.3　健康被害の補償等 54
23　臨床研究成果の帰属及び臨床研究結果の登録・公表に関するとり決め 55
臨床研究実施体制 55

23.1　研究責任者 55
23.2　研究分担者 55
23.3　連絡先 57
24　文献.. 57

0 概要

項目	内容
課題名	感染症予防を目的とした肝臓移植におけるドナー肝臓由来活性化ナチュラルキラー（NK）細胞を用いた術後免疫賦活療法の臨床応用
試験の目的	肝臓移植以外に治療法がない非代償性肝硬変患者に対して生体部分肝移植術後3日目に肝移植ドナー肝臓由来活性化ナチュラルキラー(NK)細胞を用いた免疫賦活療法を行うことで、肝臓移植後1カ月間の菌血症発現割合がヒストリカルデータに比べて低下することを非盲検試験にて検討する。
試験デザイン	広島大学病院単施設、非盲検、ヒストリカルデータ対照試験
特定細胞加工物	肝移植ドナー肝臓由来活性化ナチュラルキラー（NK）細胞
選択基準	レシピエント（再生医療を受ける者）の選択基準<br>1) 内科的治療抵抗性の非代償性肝硬変治療として生体部分肝移植術を施行する患者<br>2) 同意取得時年齢が20歳以上の患者<br>3) 本人もしくは代諾者の文書による同意が得られている患者<br><br>細胞提供者（ドナー）の選択基準<br>1) 肝移植研究会の「生体肝提供（ドナー）手術に関する指針」の基準を満たし、肝臓提供者として生体肝移植ドナー手術を受ける者<br>2) 同意取得時年齢が 20 歳以上の者<br>3) ドナー肝臓グラフトの灌流液から肝臓由来ナチュラルキラー細胞（NK）細胞を調製しレシピエントに投与することについて、本人もしくは代諾者の文書により、同意が得られている者
除外基準	レシピエントの除外基準<br>1) 再肝移植を施行する患者<br>2) 脳死肝移植を施行する患者<br>3) その他、本臨床研究への参加を研究責任者又は研究分担者が不適当と判断した患者<br><br>細胞提供者（ドナー）の除外基準<br>1) 再肝移植に対する生体肝移植ドナー手術を受ける者<br>2) 本臨床研究への参加を研究責任者又は研究分担者が不適当と判断した者

被験者の同意	肝移植術前に同意説明を行い、被験者本人（ドナー、レシピエント）もしくは代諾者による同意を得る。<br>研究責任者又は研究分担者は、本臨床研究への参加候補となる被験者本人もしくは代諾者に対して、同意説明文書を提供・使用し、口頭で十分な説明を行った後、本臨床研究への参加の同意を文書で取得する。
評価項目	主要評価項目<br>術後1カ月間の菌血症発症率<br>副次評価項目<br>① 全生存期間（術後 6 カ月、1、3 年）<br>② 免疫応答への影響<br>CFSE-MLR によるドナー特異的免疫応答性の評価（術後 1、2、3、4 週）<br>DSA（donor specific antibody）の検出（年 1 回のスクリーニング）<br>拒絶反応発症率<br>レシピエント末梢血 NK 細胞活性評価<br>③ 肝細胞癌再発の有無（肝細胞癌症例のみ）<br>De novo 発がんの有無、時期<br>④ 感染症発症率（細菌感染症、サイトメガロウイルス感染症、真菌感染症、遺伝子多型別解析）<br>⑤ 安全性評価（有害事象の種類と重症度、発現頻度、発現期間、因果関係）
研究方法<br>（治療<br>スケジュール）	１．細胞の採取<br>広島大学病院　手術室で、ドナー肝臓グラフトの灌流液を清潔操作で採取する。採取された肝灌流液は500mL polypropylene（PP）centrifuge tube（以下500mlチューブ）に回収する。500mlチューブは清潔バックを3重にして密閉し、氷詰めしたクーラーボックスに入れる。輸送の間は氷上で保管する。<br>２．細胞の受け入れ<br>手術室で採取された肝灌流液入り500mlチューブを、広島大学病院未来医療センター　細胞療法室のパスボックスを経由して品質管理者もしくは細胞調整担当者が受け入れる。受け取った肝灌流液は直ちに加工を開始する。<br>３．細胞の加工<br>細胞加工作業は全て広島大学病院　未来医療センター　細胞療法室内で標準手順書に従って行う。500mlチューブを遠心機で遠心し

	て、バイオハザード対策用安全キャビネット内で上清を廃棄する。血球成分を生理食塩水で希釈した後、単核細胞分離用滅菌試薬（Ficoll-Paque PREMIUM）を用いた比重遠沈法で遠心してリンパ球分画を抽出する。肝臓由来リンパ球の細胞数、生存率を確認した後に、ヒトIL-2（Interleukin-2）製剤（イムネース注35）及びドナー血漿（最終濃度2%）を混じた細胞培地（X-VIVO medium）に肝由来リンパ球を浮遊させる。CO_2インキュベータで3日間培養を行う。培養開始前に一部を採取し、エンドトキシンテストと細菌培養検査を行う。回収1日前に、CD3陽性T細胞分画を除去する目的で細胞培地にGMP（Good manufacturing practice）に準じた抗CD3抗体を混注する。このときにも培養上清の一部を採取し、エンドトキシンテストと細菌培養検査を行う。培養3日目、細胞浮遊液を遠心して生理食塩水で洗浄を行う。培養後の細胞数、生存率を確認する。この際、細胞状態とグラム染色による無菌試験法、エンドトキシン試験法、マイコプラズマ否定試験および細菌培養による検査を行う。投与予定の細胞浮遊液をアルブミン加生理食塩水に浮遊させる。<br>４．細胞の出荷<br>臨床研究管理者及び品質管理者が、試験物の出荷判定を行う。感染検査は、培養開始時のエンドトキシン検査、細菌培養検査の結果に加え、細胞回収時の細胞状態及びグラム染色による無菌試験法の結果をもって出荷判定に供する。<br>５．細胞の投与<br>肝移植術後3日目、レシピエント主担当医の許可を得て、肝移植レシピエントに経静脈的に細胞浮遊液を30分から1時間かけて投与する。なお、細胞の投与は術後3日目の1回のみである。
併用禁止薬剤及び併用禁止療法	特になし
観察・検査スケジュールの概略	観察・検査スケジュール表を参照
目標登録被験者数	37 症例<br>＜設定根拠＞<br>2004 年 1 月から 2009 年 12 月までに行った生体肝移植 114 例に対する NK 療法群 21 例とマッチングした非 NK 療法群 21 例において、生体肝移植術後 1 カ月の菌血症発症率を比較した先行研究の結果では、非 NK 療法群の菌血症発症率が 30%であったのに対して

	NK 療法群の菌血症発症率が 10%であった（Tashiro et al., Transplantation, 2011）。<br>この結果に基づき、NK 療法における生体肝移植後 1 カ月の菌血症発症率の期待値を 10%とし、閾値として非 NK 療法での発症率 30%を有意水準（両側）5%、検出力 80%で下回ることを調べるために必要症例数を算出すると 34 例となった。登録時における脱落例を考慮し、37 例を目標症例数とした。
研究実施期間	研究実施期間：研究届出日～2024 年 12 月 31 日（9 年間）<br>症例登録期間：研究届出日～2021 年 12 月 31 日（6 年間）

シェーマ

試験デザイン

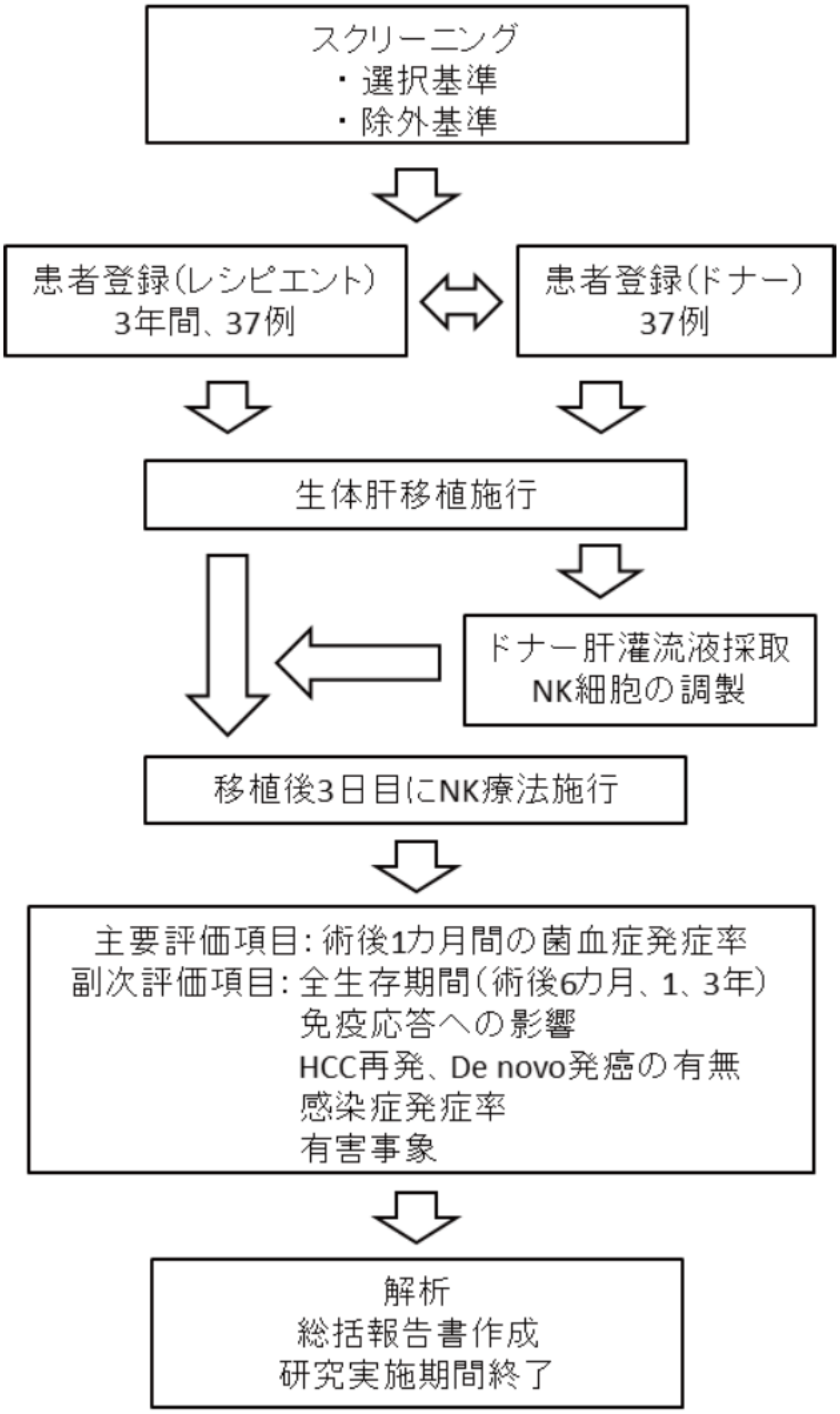

スクリーニング
・選択基準
・除外基準
患者登録(レシピエント)
3年間、37例
患者登録(ドナー)
37例
生体肝移植施行
ドナー肝灌流液採取
NK細胞の調製
移植後3日目にNK療法施行
主要評価項目: 術後1カ月間の菌血症発症率
副次評価項目: 全生存期間(術後6カ月、1、3年)
免疫応答への影響
HCC再発、De novo発癌の有無
感染症発症率
有害事象
解析
総括報告書作成
研究実施期間終了

観察・検査スケジュール

【被験者（レシピエント）】

観察日・評価日	同意取得	スクリーニング	術前検査	登録	手術日	細胞投与日	細胞投与日	4日後	5日後	1週間後	2週間後	4週間後	8週間後	12週間後	6か月後	以後6カ月毎3年まで
許容範囲	手術前4週間以内	手術前4週間以内	手術前4週間以内	手術前4週間以内		投与前	投与後	投与翌日	投与2日後	±2日	±2日	±1週	±1週	±1週	±8週	±8週
同意取得	○															
登録				○												
被験者情報		○			○											
臨床症状			○			○	○	○	○	○	○	○	○	○	○	○
バイタルサイン			○			○	○	○	○	○	○	○	○	○	○	○
血液検査			○			○	○	○	○	○	○	○	○	○	○	○
尿検査			○			○		○	○	○	○	○				
胸部Xp			○			○		○	○	○	○	○				
培養			○							○	○	○				
CT			○									○		○	○	○
フローサイト			○			○				○	○	○				
MLR			○							○	○	○				
有害事象							○	○	○	○	○	○	○	○	○	○
併用治療							○	○	○	○	○	○	○	○	○	○

【細胞提供者（ドナー）】

観察日･評価日	同意取得	術前検査	登録	手術
許容範囲	手術 4 週間以内			
同意取得	○			
被験者情報*		○		
臨床症状*		○		
バイタルサイン*		○		
血液検査*		○		
ドナーグラフト肝灌流の採取				○

*：生体肝移植の適合性を確認するために術前 3 カ月以内に観察されている場合には、その観察・検査データを利用することとする。

語句の定義

▪ 肝臓由来ナチュラルキラー（NK）細胞

肝臓は組織中に内在する各種リンパ球の含有率、特に NK 細胞の含有率が他の臓器と比較し非常に高いことが知られている。我々は正常肝から採取したこの肝臓由来 NK 細胞が肝癌細胞株に対し高い抗腫瘍活性を有することを報告している。

▪ 生体部分肝移植術

レシピエントの全肝摘出後、ドナーより採取した部分肝臓を移植する方法で、非代償性肝不全に対する根本的治療として行われる治療法である。諸外国では脳死ドナーからの臓器提供による脳死肝移植が一般的である国も多いが、本邦では脳死ドナーからの臓器提供が少ないため、生体ドナーからの部分肝移植術が最も一般的に行われている。

▪ 試験物調製施設

試験物であるドナー肝臓由来リンパ球を、標準手順書に基づいて無菌的に調製する構造・設備・管理体制を備えた施設。本臨床研究においては、広島大学病院　未来医療センター内でプロトコル治療に用いられる試験物を調製する。

背景（平易な表現）：

末期の肝不全患者にとって、肝臓移植は唯一の治療法です。肝臓移植手術を受けると、拒絶反応を防ぐために免疫抑制剤というお薬を飲む必要があります。免疫抑制剤は、身体の抵抗力を下げてしまうため、ばい菌やウイルスなどによる感染症にかかりやすくなります。特に、手術直後にはたくさんの免疫抑制剤が必要となるため、命にかかわる感染症にかかる確率が高くなります。

内容（平易な表現）：

肝臓移植で使用するドナーの肝臓内には癌やウイルスを攻撃する力の強い細胞（ナチュラルキラー細胞）がたくさん含まれています。肝臓移植の手術中にこの細胞を取り出し、3 日間培養するとその攻撃力が増強します。肝臓移植後 3 日目に、活性化したナチュラルキラー細胞をレシピエントに注射します。ナチュラルキラー細胞は、免疫抑制剤に比較的抵抗性を持っていて、手術直後の感染症を予防する効果が期待されています。

1 研究目的

本臨床研究の目的は、肝臓移植以外に治療法がない非代償性肝硬変患者に対して生体部分肝移植術後 3 日目に肝移植ドナー肝臓由来活性化ナチュラルキラー（NK）細胞を用いた免疫賦活療法を行うことで、肝臓移植後の重症感染症の発現を低減することが可能かをヒストリカルデータと比較する非盲検試験である。本研究では、肝臓移植後の血流感染（菌血症）発症頻度および NK 細胞投与の安全性を検討する。

2 経緯

2.1 対象疾患

肝臓移植を必要とする非代償性肝硬変患者

2.1.1 概念・定義・病因・病態

肝移植手術は非代償性肝硬変に対する唯一の根治的治療法であり、近年移植後成績は術式の標準化、免疫抑制剤の進歩、周術期管理の向上により著しく向上した。しかしながら拒絶反応を予防するため免疫抑制剤の投与が必須であることなどから、感染症の診断の遅れや重症化をしばしば経験する。感染症が局所から全身性に波及する病態として血流感染が挙げられ、臨床症状を伴った菌血症と定義される（CDC ガイドライン）。特に術後早期の強力な免疫抑制下では、ひとたび細菌や真菌による血流感染が発症すると予後不良であり、さらなる移植成績の向上のため克服すべき課題のひとつである。

2.1.2 疫学

肝臓移植患者の 60～80 %は、術後 1 年以内に何らかの感染症を経験する。その多くの感染症は治癒するが、時に致命的となる(Colonna et al., Arch Surg, 123,p.360, 1988; Kawecki et al., Med Sci Monit, 15,p.CR628, 2009)。感染症の内訳は、約半数が細菌感染、4 割がウイルス感染、1 割が真菌感染症である(Fishman & Rubin, N Engl J Med, 338,p.1741, 1998; Winston, Emmanouilides, & Busuttil, Clin Infect Dis, 21,p.1077, 1995)。特に、移植後 1 カ月以内は感染症の頻度が最も高い。また、血流感染は移植後死因の 30 %に関与していると報告されている。菌血症の原因として、中心静脈カテーテル感染（30 %）、胆管炎（30 %）、腹腔内感染（23 %）が挙げられ、検出菌種はグラム陽性球菌とグラム陰性桿菌が多い(Kim et al., Yonsei Med J, 50,p.112, 2009)。

2.1.3　標準治療と予後

肝臓移植後の細菌感染および真菌感染に対する予防的抗菌薬、抗真菌薬投与は無作為化臨床比較試験に基づいたエビデンスは少なく、薬剤選択・方法に関して一定の見解は得られていない。施設により選択する薬剤は異なるものの 2-3 世代セファロスポリン単剤を予防投与 2-3 日間用いる施設が多い(Soave, Clin Infect Dis, 33 Suppl 1,p.S26, 2001)。

2.1.4　対象疾患の設定根拠

肝臓移植後の死因の 1 位は感染症であり、感染症による死亡を減少させることが本研究の目的である。一般的に肝臓移植後の感染症で予後に関わる重篤なものとして菌血症が挙げられるため、本臨床研究では術後早期の菌血症を対象とする。

2.2　特定細胞加工物名及びその概要

2.2.1　特定細胞加工物名

肝移植ドナー肝臓由来活性化ナチュラルキラー（NK）細胞

2.2.2　試験物の概要

生体部分肝移植を施行するにあたり、ドナーから摘出したグラフト肝臓は GVHD（移植片対宿主病）の予防および臓器保存の目的で経門脈的な肝灌流が必須の手技となる。通常破棄されるこの肝灌流液には末梢血と比較し、非常に豊富な肝由来リンパ球（T 細胞、B 細胞、NK 細胞、NKT 細胞）が含まれている。この肝由来リンパ球を IL-2 存在下（100IU/ml）で 3 日間培養することにより、NK 細胞分画の増殖および活性化を誘導することが可能である。活性化した肝内リンパ球に含まれる活性化 NK 細胞は in vitro で肝癌細胞株に対し高い細胞傷害活性を有することが確認されている。また、マウスに 70%肝切除を行うことにより肝内 NK 細胞の細胞傷害性分子の発現低下が誘導され、経門脈的に肝癌腫瘍株を投与すると肝臓内に癌組織の生着が認められる（肝内転移モデル）が、正常マウスの肝灌流液中に存在する NK 細胞を活性化し投与することで癌の生着を抑制することできることを確認している。活性化した肝由来リンパ球には NK 細胞分画が最も多い分画として認められるが、GVHD を惹起しうる T 細胞分画も存在していることから、投与 1 日前に抗 CD3 抗体（1μg/ml）を培地に加え、CD3 陽性 T 細胞の除去を行う。

この細胞の利点は、手術手技として必須であり通常破棄されるドナー肝臓の灌流液を使用すること、in vitro での単純培養により活性化とともに細胞傷害性の強い NK 細胞を優先的に増殖させることが可能であることである。そのため細胞採取に侵襲性を伴わず、臨床応用が比較的容易である。

2.2.3　これまでの前臨床試験、臨床研究及び臨床試験の結果の要約

前臨床試験

- マウスにおける肝内NK細胞のTRAIL分子を介した自己肝細胞に対する細胞傷害性 (Ochi et al., Hepatology, 39,p.1321, 2004)

　マウス肝由来NK細胞は脾臓由来NK細胞とは異なり、定常状態で30-40%にtumor necrosis factor-related apoptosis-inducing ligand（TRAIL）を発現しており、Poly I:C刺激によってその発現が増強された。In vitroの細胞傷害性試験においてマウス肝由来NK細胞はnaïveな状態でも自己肝細胞に対し細胞傷害性を示し、Poly I:C刺激後その細胞傷害活性はさらに増強を認めた。抗TRAIL抗体の投与により、その細胞傷害性は減弱することが確認された。マウス肝由来TRAIL陽性NK細胞はMHC class Iを認識する抑制性受容体であるLy49の発現が欠如していた。また、Poly I:C刺激によりTRAIL陰性NK細胞のLy49の発現は増強されたが、TRAIL陽性細胞では発現の増強は認めなかった。マウス肝由来TRAIL陽性NK細胞は自己認識能の低下から自己肝細胞に対する細胞傷害性を生ずると考えられ、NK細胞の細胞傷害機構におけるTRAIL分子の重要性が確認された。

- ヒト肝内NK細胞の肝細胞癌に対する細胞傷害性(Ishiyama et al., Hepatology, 43,p.362, 2006)

　ヒト肝臓灌流液から採取された肝由来NK細胞はnaïveな状態では末梢血NK細胞と同様にTRAILの発現を認めないが、IL-2で刺激することによって末梢血NK細胞と比較し高度にTRAIL発現が誘導された。ヒト正常肝由来NK細胞はIL-2で刺激することにより末梢血や硬変肝由来NK細胞と比較し、肝癌細胞株に対し高度な細胞傷害活性を示した。また、ヒトにおいてはIL-2刺激によってTRAILの発現とともにHLA class Iを認識する抑制性受容体の発現が誘導された。また、正常肝組織にはTRAILの機能的受容体であるdeath receptor (DR)4/DR5と共に競合的に作用するdecoy receptor（DcR）1/DcR2の発現が認められるのに対し、肝細胞癌組織では分化度の低下とともにDR4/DR5の発現が増強し、DcR1/DcR2の発現が低下していた。

　細胞傷害性試験において、IL-2刺激後の正常肝由来NK細胞は自己およびHLAハプロタイプの一致した肝細胞に対し細胞傷害性をほとんど示さなかった。

　以上のことから、正常肝由来NK細胞がIL-2刺激により肝細胞癌を選択的に傷害し、肝移植後の肝細胞癌再発を抑制する可能性が示された。

- マウス肝転移モデルにおける TRAIL 肝内 NK 細胞による肝転移抑制効果(Ohira et al., Transplantation, 82,p.1712, 2006)

  マウス大量肝切モデルにおいて肝内の TRAIL 陽性 NK 細胞の減少を認め、肝由来 NK 細胞の抗腫瘍活性は低下した。無処置マウスでは経門脈的に肝癌細胞株を投与しても宿主免疫により腫瘍生着は得られないが、上記大量肝切除マウスに経門脈的に肝癌細胞株を投与することにより肝内転移を生ずる肝内転移マウスモデルを作製した。この肝内転移マウスモデルに経門脈的腫瘍投与とともに,Poly I:C によって刺激したマウスから採取した活性化肝由来 NK 細胞を同時投与することにより腫瘍生着が抑制されることが明らかになった。

- ヒト肝キメラマウスにおけるドナー肝臓由来活性化リンパ球による HCV（C 型肝炎ウイルス）感染抑制効果(Ohira et al., J Clin Invest, 119,p.3226, 2009)

  ヒト肝灌流液から得られたリンパ球は、NK 細胞と NKT 細胞が豊富に含まれている。本臨床試験のプロトコルを用いて、肝臓由来リンパ球に IL-2（100IU/ml）及び抗 CD3 抗体(1ug/ml)を加えると、HCV レプリコン細胞を用いたアッセイで強力な抗 HCV 効果が確認された。また、ヒト肝臓由来活性化リンパ球を HCV 感染ヒト肝キメラマウスに投与すると、HCV 感染を抑制した。さらに抗 IFN-γ（インターフェロンガンマ)抗体を同時に投与することで抗 HCV 効果が相殺されるため、IFN- γ が責任分子であることが明らかとなった。

- 脳死肝移植ドナー肝灌流液から得られた NK 細胞は強力な抗腫瘍活性を持つ(Ohira et al., Cell Transplant, 21,p.1397, 2012)

  脳死肝移植患者に本臨床研究を応用するために、米国マイアミ大学において脳死肝移植ドナーの肝灌流液を採取し cGMP 施設で細胞加工を行った。生体肝移植ドナーの場合と同様に、NK 細胞を豊富に含有し、IL-2 刺激により強力な抗腫瘍活性を獲得することを確認した。さらに、肝癌細胞を効率よく攻撃する TRAIL 分子の良好な表出が得られた。GVHD の原因となる T 細胞の含有は 1.8 × 10^4 cells/kg と少なく、アロへの投与が許容される量であった。以上の結果より、米国 FDA（食品医薬品局）より第 1 相試験の開始が許可された。

臨床研究

基礎実験により得られた結果に基づき、2006 年 7 月 3 日「ヒト幹細胞を用いる臨床研究に関する指針」通知前に、肝細胞癌合併肝移植症例を対象として、肝移植術後の肝細胞癌再発制御を目的とするドナー肝由来活性化 NK 細胞療法の安全性試験を開始した。その臨床成績を以下に示した。

■ 広島大学における生体肝移植術後肝細胞癌再発予防を目的とした活性化 NK 細胞療法（論文作成中）

ドナー肝臓由来活性化リンパ球移入療法の安全性の検証とともに、同時期に施行した免疫療法非施行症例との臨床成績の検討を行った。24 例の術前ミラノ基準内肝細胞癌合併肝不全患者に対し、肝移植術後免疫補助療法としてドナー肝由来活性化リンパ球移入療法を施行した。患者年齢中央値は 58 歳、男性 17 例、女性 7 例であった。投与された活性化リンパ球中央値は 270（38-820）× 10^6 cell/body であった。5 年全生存率、5 年無再発生存率はそれぞれ 82.5%、74.5%であった。免疫療法施行例に GVHD は認めず、その他重大な投与関連有害事象は認めなかった。高用量投与群（> 270 × 10^6 cell/body）と低用量投与群（≦ 270 × 10^6 cell/body）との比較においても Grade3/4（CTCAE ver.4）以上の有害事象の発生頻度に有意差は認めなかった。同時期に施行した免疫療法非施行症例との比較において、病理学的ミラノ基準逸脱症例の全生存、無再発生存は免疫療法施行群で有意に良好であった。CFSE-MLR（CFSE 色素を用いたリンパ球混合試験）による定量的抗ドナー反応は免疫療法施行群及び非施行群に差を認めなかった。

■ ドナー肝臓由来活性化リンパ球を用いた術後補助療法の生体肝移植後血流感染症に対する影響(Tashiro et al., Transplantation, 92,p.575, 2011)

2004 年から 2009 年までに広島大学病院にて施行した 114 例の生体肝移植症例の術後早期の血流感染の発症頻度を解析した。ドナー肝由来活性化リンパ球を用いた免疫補助療法を施行群 21 例と傾向スコアマッチング法を用いて背景因子を調整した免疫補助療法非施行群 21 例を比較すると免疫補助療法群の血流感染発生頻度は有意に低かった。

■ ドナー肝臓由来活性化リンパ球を用いた術後補助療法の抗 HCV 効果 (Ohira et al., J Clin Invest, 119,p.3226, 2009)

ドナー肝由来活性化 NK 細胞は、HCC に対する細胞傷害活性のみならず、HCV に対する増殖抑制効果も持っている。HCV 感染合併肝移植症例のうち、活性化 NK 細胞療法を施行した 7 例と同時期に肝移植を受けた HCV 感染患者 5 例の術後の HCV ウイルス量の推移を比較検討した。NK 細胞療法を行った群では、コントロール群に比べ、術後一か月間、有意に HCV ウイルス量を低下させた。しかし免疫療法直後は強力な抗 HCV 効果を持つものの、時間の経過とともに徐々に HCV-RNA は上昇し、効果は一時的である可能性が示唆された。

■ 米国マイアミ大学における脳死肝移植後肝細胞癌再発予防を目的とした活性化 NK 細胞療法（論文作成中）

広島大学とマイアミ大学（Miami, FL, USA）との共同研究で、2010 年 7 月から脳死ドナー由来の活性化肝 NK 細胞を用いた細胞療法の臨床試験（第 I 相）を行っている。切除不能肝細胞癌合併脳死肝移植レシピエント 18 例を対象とした。患者年齢中央値は 60 歳、男性 16 例、女性 2 例であった。GVHD をはじめ、本療法が原因と考えられる重大な有害事象は認められなかった。18 例中 9 例が術後病理診断でミラノ基準を超える症例であったが、平均観察期間 31 ヶ月の時点で HCC 再発を認めた症例はなかった。また、NK 療法施行症例は、マイアミ大学における historical control と比べ、生存率は良好であった。

2.2.4　臨床研究実施が可能であると判断した理由

ドナー肝臓由来活性化リンパ球を用いた術後補助免疫療法の安全性については、厚生労働省の「ヒト幹細胞を用いる臨床研究に関する指針」の施行以前に開始した第 1 相臨床試験において、臨床的に細胞移入に伴う有害事象が認められなかった。また、臨床効果については前臨床試験において術前ミラノ基準内かつ病理学的ミラノ基準逸脱症例において再発予後を改善する可能性とともに肝移植術後早期の血流感染発症頻度を抑制する可能性が示されており、生体肝移植術後の生存予後に寄与する可能性が示唆されている。肝移植後急性期合併症として血流感染を含む重症感染症は依然生命予後を脅かす合併症の際たるものである。様々な感染予防対策がなされてきた今日においても、術前の患者状態が不良であることや免疫抑制剤の使用を余儀なくされることから重症感染症の発症を免れない症例も少なくなく有効な予防法期待されている。以上のようなことから、臨床研究で実施が可能であると判断した。

2.3　登録患者の予想される利益と不利益

2.3.1　予想される利益

本臨床研究は、生体肝移植後ドナー肝臓由来活性化リンパ球に含まれる活性化 NK 細胞の臨床的有効性を評価するものである。生体肝移植は非代償性肝硬変に対する唯一の根本的治療として普及してきているが、高度の手術侵襲や術後の免疫抑制下で重症感染症を合併すれば、その生命予後は極めて不良である。本臨床研究における生体肝移植後ドナー肝臓由来活性化 NK 細胞の臨床的有効性が確認されれば、重症感染症の発現が低減し、生体肝移植術の術後予後に大きく貢献することとなる。

なお、本臨床研究に参加することにより被験者が報酬などの利益を受けることは一切無い。また、本臨床研究により生じる知的財産権は研究者に帰属するものとし、それにより被験者が治療効果以外に利益を受けることはない。

2.3.2　予想される不利益

本臨床研究における被験者は、「予想される有害事象」に挙げる有害事象の他に予期せぬ有害事象が生じる可能性があるが、その際には研究期間終了後であっても速やかに適切な処置と治療をもって対処する。ドナー感染症検査が、ウインドウピリオドにより偽陰性となる危険性があり、感染が判明した場合は適切な処置を行う。

なお、肝臓移植にかかる費用は通常診療として患者負担であるが、本臨床研究における治療にかかる費用は研究費によって行われ、被験者による負担は生じない。試験物の提供者である生体肝移植ドナーに対しては、本来破棄される灌流液を回収、使用するため、通常行われるドナー肝摘出術に関わる危険性以外は生じない。

2.4　本臨床研究の意義

本臨床研究の意義は、肝臓移植以外に治療法がない非代償性肝硬変患者に対して生体部分肝移植術後3日目に生体肝移植後ドナー肝臓由来活性化NK細胞を用いた臨床的有効性を明らかにし、生体肝移植術の成績向上を図るものである。本臨床研究で生体肝移植後ドナー肝臓由来活性化NK細胞の臨床的有効性および安全性の確認をした後には先進医療Bとして本治療法の更なる有効性の検証を目標とする。

このことは最終的には肝臓移植患者の予後向上に大きく寄与することが期待される。

3 対象疾患と適格基準

3.1　対象疾患

生体肝移植が必要な非代償性肝硬変患者

3.2　選択基準

○　レシピエント（再生医療を受ける者）の選択基準

以下に挙げた全ての項目を満たす患者を選択する。

1. 内科的治療抵抗性の非代償性肝硬変治療として生体部分肝移植術を施行する患者
2. 同意取得時年齢が 20 歳以上の患者
3. 本人もしくは代諾者の文書による本研究への参加の同意が得られている患者

【レシピエント（再生医療を受ける者）の選択基準の設定根拠】

1. 本臨床研究は、非代償性肝硬変患者に対する生体肝移植後の重症感染症発症予防に対する治療効果を評価するため選択基準として設定した。

2. 生体肝移植術を受けられることが前提となる被験者に対し、年齢上限を設定する必要はないと考え年齢 20 歳以上とした。
3. 倫理性を考慮した上で、臨床研究を理解し期間を通じて臨床研究に協力できる患者を組み入れるために設定した。

○ 細胞提供者（ドナー）の選択基準

以下に挙げた全ての項目を満たす患者を選択する。

1. 肝移植研究会の「生体肝提供（ドナー）手術に関する指針」の基準を満たし、肝臓提供者として生体肝移植ドナー手術を受ける者
2. 同意取得時年齢が 20 歳以上の者
3. ドナー肝臓グラフトの灌流液から肝臓由来ナチュラルキラー細胞（NK）細胞を調製しレシピエントに投与することについて、本人もしくは代諾者の文書により、同意が得られている者

【細胞提供者（ドナー）の選択基準の設定根拠】

1. 本臨床研究で使用する細胞は肝臓移植手術時に、グラフト肝臓内より採取するものであり、肝臓提供者として生体肝移植ドナー手術を受ける者と設定した。
2. 生体肝移植ドナー術を受けられることが前提となる細胞提供者に対し、安全性を保証するために 20 歳以上とした。
3. 倫理性を考慮した上で、肝移植ドナー肝臓由来活性化ナチュラルキラー細胞をレシピエントに投与する本臨床研究を十分に理解し、研究に協力できるドナーを組み入れるため

3.3 除外基準

○ レシピエントの除外基準

以下のいずれかの項目に該当する患者は、対象から除外する。

1. 再肝移植を施行する患者
2. 脳死肝移植を施行する患者
3. その他、本臨床研究への参加を研究責任者又は研究分担者が不適当と判断した患者

【レシピエントの除外基準の設定根拠】

1. 初回肝移植術による治療が本臨床研究の評価に影響を及ぼすため
2. 本試験プロトコルに準じた試験物採取、調整ができないため
3. 研究の推進と患者の尊厳に留意して適切で柔軟な対応をするため

○　細胞提供者（ドナー）の除外基準

以下のいずれかの項目に該当する者は、対象から除外する。

1. 再肝移植に対する生体肝移植ドナー手術を受ける者
2. 本臨床研究への参加を研究責任者又は研究分担者が不適当と判断した者

【細胞提供者（ドナー）の除外基準の設定根拠】

1. 初回肝移植術による治療が本臨床研究の評価に影響を及ぼすため
2. 研究の推進と患者の尊厳に留意して適切で柔軟な対応をするため

4　同意取得

4.1　同意説明文書及び同意書の作成

研究責任者は、被験者（ドナーおよびレシピエント）および被験者の家族等の代諾者から研究参加の同意を得るために用いる同意説明文書及び同意書、同意撤回書を可能な限り平易な表現と用語を用いて作成する。

研究責任者は、広島大学再生医療等委員会で承認の得られた同意説明文書を被験者（ドナーおよびレシピエント）および代諾者に渡し、文書および口頭による十分な説明を行い、研究対象者の自由意思による同意を文書で得る。

4.2　同意説明文書及び同意書の改訂

研究分担者は、被験者（ドナーおよびレシピエント）の同意に影響を及ぼすと考えられる有効性や安全性等の情報が得られたときや、被験者（ドナーおよびレシピエント）の同意に影響を及ぼすような実施計画等の変更が行われるときは、速やかに被験者（ドナーおよびレシピエント）および代諾者に情報提供し、本研究に参加するか否かについて被験者（ドナーおよびレシピエント）および代諾者の意思を予め確認するとともに、事前に広島大学再生医療等委員会の承認を得て同意説明文書等の改訂を行い、再同意を得ることとする。なお、すでに臨床研究が終了している被験者（ドナーおよびレシピエント）にはその限りではない。

4.3　同意説明及び同意取得の時期及び方法

研究責任者又は研究分担者は、広島大学再生医療等委員会等で承認の得られた同意説明文書を用いて、本臨床研究への参加候補となる被験者（ドナーおよびレシピエント）及び代諾者に対して、同意説明文書を提供・使用し、口頭で十分な説明を行った後、本

臨床研究への参加の同意を文書で取得する。被験者（ドナーおよびレシピエント）及び代諾者が本臨床研究の内容をよく理解したことを確認した上で、スクリーニング検査を実施するまでに文書で自由意志による同意を取得する。

5 登録

5.1 被験者登録

研究責任者又は研究分担者は文書による同意を取得した後に、以下の手順に従い被験者の適格性の確認、登録を行うものとする。

1. 被験者名簿の作成
   研究責任者、研究分担者又は研究協力者は、臨床研究参加に文書で同意を得た患者を研究事務局に報告し、個人情報管理者が連結可能匿名化した被験者識別コードが付与され、匿名化した状態でデータのみを収集する。個人情報管理者は、個人識別情報とその対応表を外部とは独立したパーソナルコンピュータで管理し、個人情報管理者しか知らないパスワードを設定し、コンピュータをセキュリティーの厳重な部屋に保管することにより、情報の漏洩に対する安全対策を講じる。また、対応表は外部に提供することはない。

2. 適格性の判定
   研究責任者又は研究分担者は、同意取得後にスクリーニング及び術前検査を実施する。患者背景及び術前検査結果に基づいて、「対象疾患と適格基準」で規定する登録時の選択基準のすべての項目を満たし、除外基準のいずれの項目にも該当しないことを確認する。

3. 被験者の登録
   症例登録は、症例報告書に従って必要事項を入力し、研究事務局にて被験者の確認を行い、登録する。

4. プロトコル治療の開始
   研究責任者又は研究分担者は、研究事務局からの登録完了報告を受けた後に生体肝移植術施行とともに試験物の回収、調整を開始する。

6 研究で用いる特定細胞加工物

6.1 特定細胞加工物名

肝移植ドナー肝臓由来活性化ナチュラルキラー（NK）細胞

6.2 成分・構造・特性・製造方法

6.2.1 規格

1） 細胞数：10×10^6 個以上
2） トリパンブルー色素排除試験　細胞生存率：80%以上
3） グラム染色*：陰性
4） エンドトキシン試験：5.0EU/mL 以下

*：3)は出荷当日の培養上清の検査結果による

6.2.2 製造方法

細胞移植群で用いられる試験物の調製はすべてあらかじめ別途定められた標準作業手順書に従う。

ドナー肝臓灌流液の採取

プロトコル治療実施研究機関で定められた手順書に従ってドナーグラフトに経門脈的肝内灌流を施行し、清潔操作で灌流排液の採取を行い、直ちに検体搬送容器に保存して試験物調製施設に搬送する。細胞採取に際して、細胞採取を優先して医学的処置、手術及びその他の治療方針に変更は生じない。

細胞の調製

試験物調製施設の使用に関する教育訓練を受けた細胞調整担当者が、あらかじめ定められた手順書に従って細胞の調製を行う。肝灌流液入りの 500ml チューブを遠心機で遠心して、バイオハザード対策用安全キャビネット内で上清を廃棄する。血球成分を生理食塩水で稀釈した後、単核細胞分離用滅菌試薬（Ficoll-Paque PREMIUM）を用いた比重遠沈法で遠心してリンパ球分画を抽出する。肝臓由来リンパ球の細胞数、生存率を確認した後に、ヒト IL-2 製剤（イムネース注 35）及びドナー血漿（最終濃度 2%）を混じた細胞培地(X-VIVO medium)に肝由来リンパ球を浮遊させる。CO_2インキュベータで 3 日間培養を行う。培養開始前に一部を採取し、エンドトキシンテストと細菌培養検査を行う。回収 1 日前に、CD3 陽性 T 細胞分画を除去する目的で細胞培地に GMP に準じた抗 CD3 抗

体（ミルテニー）を混注する。このときにも培養上清の一部を採取し、エンドトキシンテストと細菌培養検査を行う。培養 3 日目、細胞浮遊液を遠心して生理食塩水で洗浄を行う。培養後の細胞数、生存率を確認する。この際、細胞状態とグラム染色による無菌試験法、エンドトキシン試験法、マイコプラズマ否定試験および細菌培養による検査を行う。投与予定の細胞浮遊液をアルブミン加生理食塩水に浮遊させる。

感染症検査

肝臓由来リンパ球分離時培地を好気性培養検査、嫌気性培養検査、エンドトキシン検査に提出する。培養 2 日目投与 12 時間前に細胞培地 2ml を再度培養検査に提出する。投与当日に細胞培養上清より 2ml を培養検査に分注し、培養検査室にてグラム染色にて感染の有無を確認とともに培養経過中に提出した感染症検査結果と総合し判断する。出荷当日の培養上清よりマイコプラズマ否定試験、エンドトキシン検査及び培養検査を提出するが、出荷判断には含めない。

6.3　容器・包装・保存条件

特定細胞加工物の入った容器に、被験者が識別可能なラベル表示を貼付し、試験検査の結果による出荷判定が終わるまで室温保管する。

出荷判定後、特定細胞加工物の入った容器を包装し、出荷する。

6.4　交付・搬送

ラベルを添付した特定細胞加工物が入った容器を包装し、細胞培養加工施設から搬出し、搬送又は搬送準備する。搬送担当者は、献体搬送容器に入れて特定細胞加工物を移植手術実施場所へ運搬する。移植手術実施場所に到着後、搬送担当者は特定細胞加工物を担当者に受け渡す。

6.5　管理・保管

移植手術実施場所到着後、担当の医師は被験者を識別するための番号と特定細胞加工物のラベルに記載された番号、品質保証書等を確認し、受け取る。使用まで特定細胞加工物は、移植手術実施場所の所定の場所で保管する。使用期限は出荷当日中とする。

7 試験デザイン

7.1　　試験デザイン

単施設、ヒストリカルデータ対照、非盲検試験

【デザインの設定根拠】

本臨床研究は、肝移植ドナー肝臓由来活性化ナチュラルキラー（NK）細胞を用いた免疫賦活療法による肝臓移植後の重症感染予防の Phase I/II 試験である。移植グラフトより採取した肝由来活性化リンパ球投与については、先行して実施した Phase I 試験および米国の共同研究グループが脳死肝移植症例を対象に実施した Phase I 試験によって一定の安全性及び効果が確認されている。本試験では、先行研究の結果から非 NK 療法群の菌血症発症率（割合）30%を閾値とし、NK 療法施行時の菌血症発症率（割合）が閾値を下回ること、NK 療法施行時の安全性情報をより多く収集するために非盲検試験として行う。

なお、移植する細胞数としては、前臨床研究において投与細胞数に依存した効果が見込まれるが、細胞投与量によって有害事象の発現に差異を認めていないこと、さらに入手可能な細胞数はドナー肝臓グラフトに依存しておりコントロールが困難であることから上限の設定はしないこととした。

7.2　　目標登録被験者数・被験者登録期間

目標登録被験者数：37 例

2004 年 1 月から 2009 年 12 月までに行った生体肝移植 114 例中に対する NK 療法群 21 例とマッチングした非 NK 療法群 21 例において、生体肝移植術後 1 カ月の菌血症発

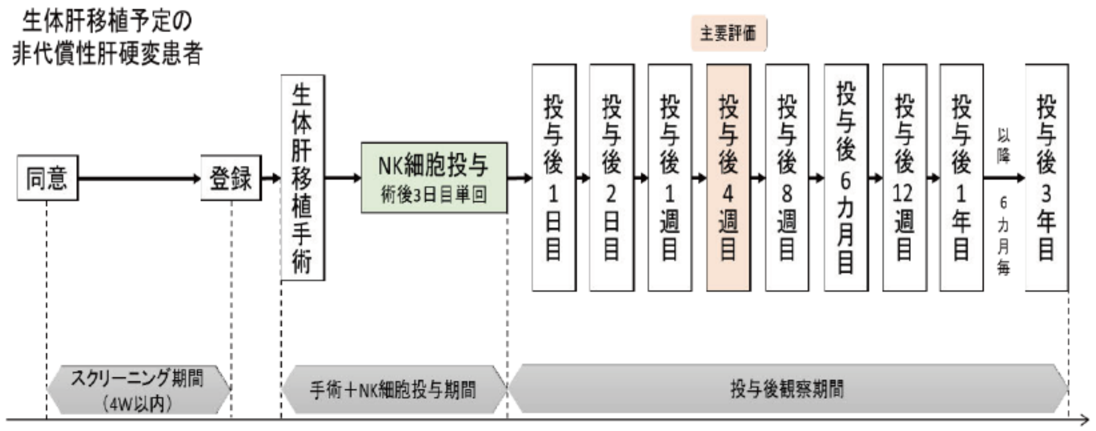

症率を比較した先行研究の結果では、非 NK 療法群の菌血症発症率が 30%であったのに対して NK 療法群の菌血症発症率が 10%であった（Tashiro et al., Transplantation, 92,p.575, 2011)。この結果に基づき、本試験の主要評価項目を生体肝移植後 1 カ月の菌血症発症率とし、NK 療法群の期待発症率を 10%とし、閾値として非 NK 療法時の発症率 30%を有意水準（両側）5%、検出力 80%で下回るために必要症例数を算出すると 34 例となる。登録時における脱落例を考慮し、37 例を目標症例数とした。

被験者登録期間：研究届出日～2021 年 12 月 31 日（3 年間）

7.3　目標登録被験者数の集積可能性

2008 年～2012 年に広島大学病院において行った肝臓移植症例は 97 例であった。広島大学病院では毎年 15 例前後の肝臓移植手術を行っていることから、本試験対象症例が上記実施例の 90%程度と見積もれば、登録期間 3 年間で 40 例程度の被験者を得ることが可能と予測され、試験参加の同意取得を考慮しても 3 年間で 37 例の登録が可能である。

7.4　プロトコル治療計画

7.4.1　プロトコル治療の定義

生体部分肝移植手術時に採取したドナーグラフト肝灌流排液から比重遠沈法によりリンパ球分画を抽出する。肝臓由来リンパ球は、IL-2（100IU/ml）及びドナー血漿（最終濃度 2%）を加えた完全培地で 3 日間培養する。投与 1 日前に、CD3 陽性 T 細胞を除去するために抗 CD3 抗体（1μg/ml）を培地に加える。細胞浮遊液を回収し、遠心分離して、生理食塩水で洗浄を行った後に、アルブミン加生理食塩水に細胞を浮遊させ、術後 3 日目に経静脈的に肝移植レシピエントに単回投与する。プロトコル治療は、ドナーグラフト肝灌流から、ドナー肝臓由来活性化 NK 細胞投与完了までと定義する。

7.4.2　方法

1）肝移植ドナー肝臓由来活性化ナチュラルキラー（NK）細胞の調製
「製造方法」の項に示した。
2）肝移植ドナー肝臓由来活性化ナチュラルキラー（NK）細胞の投与
培養後、感染症検査にて安全性の確認がなされた後に、生体部分肝移植術後 3 日目の肝移植レシピエントに経静脈的に細胞浮遊液を 30 分～1 時間かけて単回投与する。

7.4.3　併用治療

生体部分肝移植手術術前、術後に必要な治療と考えられる治療は、広島大学病院で行っている通常の生体部分肝移植術術後の管理に準じ、いずれの時期にも行うことが可能であるものとする。

7.4.4　感染症治療

感染症治療は各症例での部位、程度、治療忍容性などを総合的に判断し、担当医の判断に委ねる。

7.4.5　プロトコル治療計画の設定根拠

本臨床研究において、生体部分肝移植手術管理に対する影響はないものと考えるため、本術後補助免疫療法施行以外は従来行っている周術期管理に基づき施行するものとする。

7.4.6　登録被験者の研究参加期間

治療期間はドナーグラフト肝灌流から、ドナー肝臓由来活性化 NK 細胞投与完了まで、観察期間は生体部分肝移植手術後 3 年までとする。

7.5　中間集計

NK 細胞が投与された 15 例目の手術後 1 カ月の観察期間が終了した時点で中間集計を行い、効果安全性評価委員会による研究継続の可否について検討する。効果安全性評価委員会では、主要評価項目の達成及び安全性的観点から術後 1 カ月以内の菌血症の発症が 15 例中 6 例以上（40%以上）、NK 細胞投与との因果関係が否定できない Stage 3 以上の有害事象の発現が 2 例以上を目安として研究継続の可否を判断する。なお、効果安全性評価委員会は、本研究分担者を除く 3 名の医師とする。

8 主要評価項目及び副次評価項目

8.1　主要評価項目

術後 1 カ月間の菌血症発症率

【主要評価項目の設定根拠】

肝臓移植後の重症感染症である菌血症の発症は、先行研究の結果から手術後 1 カ月以内に起こることが多い（63%）。また、投与した NK 細胞は最長 1 カ月間レシピエント

体内で確認されたことより、NK 細胞療法による菌血症発症抑制率を評価するための期間を 1 カ月間とした。

8.2 副次評価項目

① 全生存期間（術後 6 カ月、1、3 年）
② 有害事象
本臨床研究において生じた有害事象の種類と重症度、発現頻度、発現期間、因果関係
③ 免疫応答への影響
CFSE-MLR によるドナー特異的免疫応答性の評価（術後 1、2、3、4 週）、DSA（donor specific antibody）の検出（年 1 回のスクリーニング）、拒絶反応発症率、レシピエント末梢血 NK 細胞活性評価
④ 肝細胞癌再発（肝細胞癌症例のみ）及び De novo 発がんの有無と発生時期
⑤ 感染症発症率（細菌感染症、サイトメガロウイルス感染症、真菌感染症、遺伝子多型別解析）

【副次評価項目の設定根拠】
治療効果および本治療の安全性を再評価することを目的として、副次評価項目に下記のものを設定した
① 肝移植後長期生存に対する有効性を評価するため
② 本治療の安全性を再評価するため
③ 本治療がレシピエントの免疫系に与える影響を評価するため
④ 本治療が発癌に与える影響を評価するため
⑤ 本治療が菌血症以外の感染症に与える影響を評価するため、また遺伝子多型による効果の違いを評価するため

9 観察・検査項目とスケジュール

9.1 検査スケジュール

観察・検査・評価の実施スケジュールを以下の表 1 及び表 2 に示す。研究責任者および研究分担者は、スケジュールに従って観察・検査等を実施する。なお、被験者情報（背景調査）や臨床検査など、研究協力者が実施可能な項目については、研究責任者の管理下で研究協力者が実施しても良い。

表 1：観察・検査スケジュール（被験者：レシピエント）

観察日・評価日	同意取得	スクリーニング	術前検査	登録	手術日	細胞投与日	細胞投与日	4日後	5日後	1週間後	2週間後	4週間後	8週間後	12週間後	6か月後	以後6カ月毎3年まで
許容範囲	手術前4週間以内	手術前4週間以内	手術前4週間以内	手術前4週間以内		投与前	投与後	投与翌日	投与2日後	±2日	±2日	±1週	±1週	±1週	±8週	±8週
同意取得	○															
登録				○												
被験者情報		○			○											
臨床症状			○			○	○	○	○	○	○	○	○	○	○	○
バイタルサイン			○			○	○	○	○	○	○	○	○	○	○	○
血液検査			○			○	○	○	○	○	○	○	○	○	○	○
尿検査			○			○		○	○	○	○	○				
胸部Xp			○			○		○	○	○	○	○				
培養			○							○	○	○				
CT			○									○		○	○	○
フローサイト			○			○				○	○	○				
MLR			○							○	○	○				
有害事象							○	○	○	○	○	○	○	○	○	○
併用治療							○	○	○	○	○	○	○	○	○	○

表 2：観察・検査スケジュール（被験者：ドナー）

観察日･評価日	同意取得	術前検査	登録	手術
許容範囲	手術 4 週間以内			
同意取得	○			
被験者情報*		○		
臨床症状*		○		
バイタルサイン*		○		
血液検査*		○		
ドナーグラフト肝灌流の採取				○

*：生体肝移植の適合性を確認するために術前 3 カ月以内に観察されている場合には、その観察・検査データを利用することとする。

9.2 観察・検査項目

本研究では細胞提供者であるドナーと特定細胞加工物が投与されるレシピエントが被験者であるが、実際にはレシピエントにおける特定細胞加工物投与に関する有効性及び安全性を評価する目的の研究であるため、特に断りが無い場合には観察・検査項目で測定されるのはレシピエント被験者とする。

9.2.1 スクリーニング検査

文書による同意を取得した後、以下の項目について、観察・検査を実施する。

① 被験者情報：被験者背景（生年月日、性別、身長、体重、術前状態、原疾患、腹水、脳症、術前透析、術前減感作）、現病歴、既往歴、感染症治療歴（具体的な治療内容）、肝細胞癌治療歴（肝細胞癌症例のみ）

9.2.2 術前検査

① 臨床症状：感染症状、感染治療の有無、その他の臨床症状
② バイタルサイン：血圧、脈拍数、体温
③ 血液検査：
・＜血液学的検査＞：
赤血球数、白血球数、好中球、リンパ球、ヘモグロビン、ヘマトクリット、血小板数
・＜生化学的検査＞：
総ビリルビン、間接ビリルビン、ALB、AST、ALT、γGTP、ALPH、CRP、BUN、Cre、Na、K、Cl、IgG、IgM、PT、PT-INR、AFP、L3 分画、PIVKA-II、

HBsAg、HBsAb、HBV-DNA、HCV、HCV-RNA、CMV-IgG、CMV-IgM、EBV-IgG、EBV-IgM、β-D グルカン、MELD score、Child-Pugh 分類

④ 臨床検査：
尿検査：蛋白定性、糖定性、潜血定性
血液型、HLA

⑤ 画像診断（腫瘍数、最大腫瘍径、局在の確認）：
・胸部単純 X 線：術前肝細胞癌診断（肝細胞癌症例のみ）
・造影 CT（造影剤アレルギー、腎障害時は MRI、US で代用可）

⑥ 培養検査（菌種）：鼻腔、尿
※血液培養提出基準（臨床的に菌血症が疑われる所見）：38 度以上の発熱、悪寒、低血圧、臨床症状、2 セット提出すること(Horan, Andrus, & Dudeck, Am J Infect Control, 36,p.309, 2008)

⑦ 免疫学的解析：
・末梢血フローサイトメトリー（フェノタイプ解析）：T cell %、NKT cell %、NK cell %、CD69 on NK cell、TRAIL on NK cell、NKp44 on NK cell、NKp46 on NK cell、細胞傷害性試験
・MLR（リンパ球混合試験）：CD4 T cell SI、CD8 T cell SI、CD4+CD25+T cell %、CD8+CD25+ T cell %

⑧ 細胞提供者（ドナー）情報：生年月日、性別、続柄、身長、体重、血液型、HLA、CMV-IgM、CMV-IgG、EBV-IgG、EBV-IgM、HBs-Ag、HBs-Ab、HBc-Ab、HCV-Ab、HIV-Ab、（HBV, HCV, HIV に関してはウインドウピリオドにより偽陰性の可能性があるため、術後 3 カ月をめどに再検査を行う）。
［観察時期；ドナーの同意取得後から肝移植施行日までの間］

9.2.3 登録

スクリーニング及び術前検査項目の結果から、「対象疾患と適格基準」で規定する登録時の選択基準のすべての項目を満たし、除外基準のいずれの項目にも該当しない被験者を登録する。

9.2.4 手術日

① 手術情報：グラフト種類、グラフト重量、GRWR、全虚血時間、手術時間、出血量、輸血量
② 摘出肝病理所見：病理学的ミラノ基準（肝癌合併症例のみ）、背景肝 HIA score
③ 投与細胞情報：肝灌流液の採取、肝由来リンパ球の培養、肝灌流液フローサイトメトリー

9.2.5　NK 細胞投与日（術後 3 日目）

［NK 細胞投与前］

①　臨床症状：感染症状、感染治療の有無、その他の臨床症状

②　バイタルサイン：血圧、脈拍数、体温

③　血液検査：

・＜血液学的検査＞：術前検査と同様

・＜生化学的検査＞：

総ビリルビン、直接ビリルビン、ALB、AST、ALT、γGTP、ALPH、CRP、BUN、Cre、Na、K、Cl、PT、IgG、IgM、CNI 血中濃度

④　尿検査：蛋白定性、糖定性、潜血定性

⑤　免疫学的解析：

末梢血フローサイトメトリー（フェノタイプ解析）：T cell %、NKT cell %、NK cell %、CD69 on NK cell、TRAIL on NK cell、NKp44 on NK cell、NKp46 on NK cell、細胞傷害性試験

⑥　画像診断：胸部単純 X 線

［NK 細胞投与後］

①　臨床症状：感染症状、感染治療の有無、その他の臨床症状

②　バイタルサイン：血圧、脈拍数、体温

③　血液検査：

・＜血液学的検査＞：術前検査と同様

・＜生化学的検査＞：

総ビリルビン、直接ビリルビン、ALB、AST、ALT、γGTP、ALPH、CRP、BUN、Cre、Na、K、Cl、PT

④　有害事象の観察

・有害事象名、発現日、Grade、重篤度、処置、転帰/判定日、因果関係、コメント（継続判断、因果関係、経過等の詳細）

※有害事象名は診断名で記録する。但し、診断名を付けられない症状、徴候については症状名、徴候名で記録する。

・感染症の有無、抗生剤の使用の有無、拒絶反応の有無も併せて確認する。

・患者転帰（グラフト不全の有/無、死亡の有/無、死因）、2 次移植（有/無、時期）

⑤　併用治療の確認：感染症に対する治療（予防的、先行的を含む、治療薬、投与量、投与期間）

9.2.6　NK 細胞投与 1、2 日後（術後 4、5 日目）

① 臨床症状：感染症状、感染治療の有無、その他の臨床症状
② バイタルサイン：血圧、脈拍数、体温
③ 血液検査：
・＜血液学的検査＞：術前検査と同様
・＜生化学的検査＞：NK 細胞投与日（術後 3 日目）NK 細胞投与前と同様
④ 尿検査：蛋白定性、糖定性、潜血定性
⑤ 画像診断：胸部単純 X 線
⑥ 有害事象の観察
⑦ 併用治療の確認

9.2.7　NK 細胞投与 1、2 週間後

① 臨床症状：感染症状、感染治療の有無、その他の臨床症状
② バイタルサイン：血圧、脈拍数、体温
③ 血液検査：
・＜血液学的検査＞：術前検査と同様
・＜生化学的検査＞：
総ビリルビン、直接ビリルビン、ALB、AST、ALT、γGTP、ALPH、CRP、BUN、Cre、Na、K、Cl、PT、IgG、IgM、β-D　グルカン、C7-HRP、CNI 血中濃度
④ 尿検査：蛋白定性、糖定性、潜血定性
⑤ 画像診断：胸部単純 X 線
⑥ 培養検査（菌種）：血液、喀痰、尿、胆汁、ドレーン
⑦ 免疫学的解析：
・末梢血フローサイトメトリー（フェノタイプ解析）：T cell %、NKT cell %、NK cell %、CD69 on NK cell、TRAIL on NK cell、NKp44 on NK cell、NKp46 on NK cell、細胞傷害性試験
・MLR（リンパ球混合試験）：CD4 T cell SI、CD8 T cell SI、CD4+CD25+T cell %、CD8+CD25+ T cell %
⑧ 有害事象の観察
⑨ 併用治療の確認

9.2.8　NK 細胞投与 4 週間後

① 臨床症状：感染症状、感染治療の有無、その他の臨床症状
② バイタルサイン：血圧、脈拍数、体温
③ 血液検査：

・＜血液学的検査＞：術前検査と同様
・＜生化学的検査＞：NK 細胞投与 1、2 週間後と同様

④ 尿検査：蛋白定性、糖定性、潜血定性
⑤ 画像診断：胸部単純 X 線画像診断、造影 CT（造影剤アレルギー、腎障害時は MRI、US で代用可）
⑥ 培養検査（菌種）：血液、喀痰、尿、胆汁、ドレーン
⑦ 免疫学的解析：
・末梢血フローサイトメトリー（フェノタイプ解析）：T cell %、NKT cell %、NK cell %、CD69 on NK cell、TRAIL on NK cell、NKp44 on NK cell、NKp46 on NK cell、細胞傷害性試験
・MLR（リンパ球混合試験）：CD4 T cell SI、CD8 T cell SI、CD4+CD25+T cell %、CD8+CD25+ T cell %

⑧ 有害事象の観察
⑨ 併用治療の確認

9.2.9 NK 細胞投与 8 週間後、12 週間後

① 臨床症状：感染症状、感染治療の有無、その他の臨床症状
② バイタルサイン：血圧、脈拍数、体温
③ 血液検査：
・＜血液学的検査＞：術前検査と同様
・＜生化学的検査＞：NK 細胞投与 1、2 週間後と同様

④ 有害事象の観察
⑤ 併用治療の確認

9.2.10 NK 細胞投与 6 カ月後、以降 6 カ月毎に 3 年間後まで

① 臨床症状：感染症状、感染治療の有無、その他の臨床症状
② バイタルサイン：血圧、脈拍数、体温
③ 血液検査：
・＜血液学的検査＞：術前検査と同様
・＜生化学的検査＞：
総ビリルビン、直接ビリルビン、ALB、AST、ALT、γGTP、ALPH、CRP、BUN、Cre、Na、K、Cl、PT、CNI 血中濃度

④ 画像診断：造影 CT（造影剤アレルギー、腎障害時は MRI、US で代用可）
⑤ 有害事象の観察
⑥ 併用治療の確認

9.2.11 中止時

① 中止理由

② コメント

10 被験者の安全性の確保

10.1 基本的事項

被験者の安全性を確保するために、研究責任者及び研究分担者は、以下の基本的事項を遵守する。

1. 研究責任者又は研究分担者は、被験者の選択基準及び除外基準を遵守する。
2. 被験者が、本臨床研究の研究責任者と研究分担者以外の医師の治療を受ける場合には、本臨床研究に参加していること及び本研究の内容を当該医師に通知する。
3. 本臨床研究終了後も出来る限り長期にわたって診察を行い、有害事象の発現の有無について注意を払う。
4. 被験者が健康状態の異常を感じた場合には、直ちに研究責任者又は研究分担者に連絡するよう指導する。
5. 研究責任者又は研究分担者は、被験者に有害事象が生じ、治療が必要であると認めるときは、その旨を当該患者に伝え、適切な医療を提供するとともに症例報告書に齟齬なく記載する。
6. 研究責任者は有害事象の発生状況を実施状況報告書についてまとめ、毎年１回、広島大学再生医療等委員会に提出し、管理者に報告する。

10.2 有害事象の定義

「有害事象」とは、被験者が臨床研究へ参加している間に起きる、あらゆる好ましくない又は意図しない徴候（一般臨床検査値の異常変動を含む）、症状、または病気のことであり、プロトコル治療との因果関係の有無は問わない。プロトコル治療前に存在していた症状の臨床的に有意な悪化もまた有害事象である。頻度や程度に於いて臨床的異議がないと考えられる生理的変動は有害事象として考えない。

【重篤な有害事象】

「重篤な有害事象」とは症状の程度に関わらず、以下の基準に従って、重篤か否かを判定する。

1. 死亡
2. 死亡につながる恐れのあるもの

3. 治療のために入院または入院期間の延長が必要とされるもの
4. 障害
5. 障害につながるおそれのあるもの
6. 1~5 に準じて重篤なもの
7. 後世代における先天性の疾病または異常

10.3 有害事象の評価

臨床研究の実施中に観察された有害事象は、「観察・検査項目とスケジュール」に定めたスケジュールに基づき評価する。

有害事象の重症度は NCI-Common Terminology Criteria for Adverse Events（NCI-CTC-AE Ver4.0 日本語版）に基づいて決定する。

10.4 予想される有害事象とその対応

10.4.1 予想される有害事象

生体肝移植術後肝細胞癌再発予防を目的とした活性化 NK 細胞療法を行った 24 例の先行研究において発現した有害事象は以下のとおりであり、いずれも経過観察もしくは薬物投与により軽快した。

1. Infusion reaction：発熱・倦怠感（3/24 例、12.5%）、悪寒・筋肉痛・下痢（2/24 例、8.4%）、悪心・皮疹・洞性頻脈（1/24 例、4.2%）
2. GVHD（0/24 例、0%）
3. 感染症（7/24 例、29.2%）
4. 移植細胞の汚染（ドナーの遅発性感染症発症を含む）（0/24 例、0%）

10.4.2 有害事象への対処

1. 必要に応じて、循環呼吸支持療法およびステロイド投与にて治療を行う。
2. 臨床症状に応じ、必要処置を行う。
3. 必要に応じて、抗生剤、抗ウイルス剤、抗真菌剤の投与にて治療を行う。
4. 培養試験物における無菌試験、エンドトキシン試験で陽性結果が出た場合は、直ちに投与を中止し、汚染細胞は廃棄物処理手順書に従い適切に廃棄する。すでに投与している場合は、必要に応じて抗生剤、抗ウイルス剤、抗真菌剤の投与で治療を行う。

10.5　疾病等発生時の対応

10.5.1　疾病等の発生の場合の措置

報告手順

「再生医療等の安全性の確保等に関する法律」に基づく。

研究責任者

広島大学再生医療等委員会にて重大な事態と判断された事象及び情報につき、広島大学病院管理者に対して速やかに報告しなければならない。

また、広島大学病院管理者の指示を受ける前に、必要に応じ、本臨床研究の中止または暫定的な措置を講ずることができる。

研究機関の長

1. 研究責任者から重大な事態が報告された場合には、その発生及び内容を速やかに厚生労働大臣に報告するとともに、原因の分析を含む対処方針について、速やかに広島大学再生医療等委員会の意見を聞き、当該研究責任者に対し、中止その他の必要な措置を講ずるよう指示しなければならない。なお、必要に応じ、広島大学再生医療等委員会の意見を聞く前に、研究機関の長は、当該研究責任者に対し、中止又は暫定的な措置を講ずるよう、指示することができる。
2. 研究機関の長は 1)に掲げる必要な措置を講ずるよう指示した上で、広島大学再生医療等委員会の意見、原因の分析結果及び研究責任者に指示した措置の内容を、厚生労働大臣に報告する。
3. 2)に掲げる、中止その他の必要な措置が講じられた後、その結果を厚生労働大臣に報告する。

10.5.2　認定再生医療等委員会への疾病等の報告

次に掲げる事項が発生した場合、それぞれに定める期間内に当該事項を認定再生医療等委員会に、別紙様式第 1 による報告書を提出する。

（1）　次に掲げる疾病等の発生のうち、当該再生医療等の提供によるものと疑われるもの又は当該再生医療等の提供によるものと疑われる感染症によるもの：7 日

（ア）死亡

（イ）死亡につながるおそれのある症例

（2）　次に掲げる疾病等の発生のうち、当該再生医療等の提供によるものと疑われるもの又は当該再生医療等の提供によるものと疑われる感染症によるもの：15 日

（ア）治療のために医療機関への入院又は入院期間の延長が必要とされる症例

（イ）障害

（ウ）障害につながるおそれのある症例

(エ) 重篤である症例（上のア～ウに準ずるもの）

(オ) 後世代における先天性の疾病又は異常

(3) 再生医療等の提供によるものと疑われる又は当該再生医療等の提供によるものと疑われる感染症による疾病等の発生（（1）及び（2）に掲げるものを除く。）：再生医療等提供計画を厚生労働大臣に提出した日から起算して 60 日ごとに当該期間満了後 10 日以内重大な事態を以下に定義する。

10.5.3 厚生労働大臣への疾病等の報告

次に掲げる事項が発生した場合、それぞれに定める期間内に当該事項を厚生労働大臣に、別紙様式第 2 による報告書を提出する。

(1) 次に掲げる疾病等の発生のうち、当該再生医療等の提供によるものと疑われるもの又は当該再生医療等の提供によるものと疑われる感染症によるもの 7 日

(ア) 死亡

(イ) 死亡につながるおそれのある症例

(2) 次に掲げる疾病等の発生のうち、当該再生医療等の提供によるものと疑われるもの又は当該再生医療等の提供によるものと疑われる感染症によるもの 15 日

(ア) 治療のために医療機関への入院又は入院期間の延長が必要とされる症例

(イ) 障害

(ウ) 障害につながるおそれのある症例

(エ) 重篤である症例

(オ) 後世代における先天性の疾病又は異常対象となる被験者

報告手順

「再生医療等の安全性の確保等に関する法律」に基づく。

研究責任者

広島大学再生医療等委員会にて重大な事態と判断された事象及び情報につき、広島大学病院管理者に対して速やかに報告しなければならない。

また、広島大学病院管理者の指示を受ける前に、必要に応じ、本臨床研究の中止または暫定的な措置を講ずることができる。

研究機関の長

4. 研究責任者から重大な事態が報告された場合には、その発生及び内容を速やかに厚生労働大臣に報告するとともに、原因の分析を含む対処方針について、速やかに広島大学再生医療等委員会の意見を聞き、当該研究責任者に対し、中止その他の必要な措置を講ずるよう指示しなければならない。なお、必要に応じ、広島大学再生医

療等委員会の意見を聞く前に、研究機関の長は、当該研究責任者に対し、中止又は暫定的な措置を講ずるよう、指示することができる。

5. 研究機関の長は 1)に掲げる必要な措置を講ずるよう指示した上で、広島大学再生医療等委員会の意見、原因の分析結果及び研究責任者に指示した措置の内容を、厚生労働大臣に報告する。
6. 2)に掲げる、中止その他の必要な措置が講じられた後、その結果を厚生労働大臣に報告する。

11 被験者毎の臨床研究中止の基準及び手順

11.1　被験者毎の臨床研究中止の基準

研究責任者又は研究分担者は、以下の場合には、当該被験者の臨床研究を中止・中断する。

1. 培養試験物の感染症検査にて陽性検査が出た場合。
2. 上記 1)の他、プロトコル治療が不可能となった場合
3. 被験者より臨床研究への参加に対する同意撤回の申し出があった場合
4. 有害事象の発現を認め、研究責任者が当該被験者についての臨床研究の継続が困難と判断した場合
5. 登録後、プロトコル治療開始前に被験者が適格基準を満たしていなかったことが判明した場合
6. その他、研究責任者又は研究分担者が、臨床研究の中止を適切と判断した場合

11.2　被験者毎の臨床研究中止の手順

研究責任者は、臨床研究を中止する旨を当該被験者に速やかに説明し、適切な医療の提供その他必要な措置を講じる。症例報告書に中止の理由及び中止年月日を記載する。プロトコル治療が完了した後に臨床研究を中止した場合には、被験者の観察・検査を可能な限り継続する。

12 臨床研究実施計画書の遵守、不適合の管理

12.1　臨床研究実施計画書の遵守

本臨床研究は、被験者の緊急の危険を回避するためのものである等医療上やむを得ない場合を除き、本実施計画書を遵守して実施する。

12.2　実施計画書の不適合の管理

研究責任者又は研究分担者は、被験者の緊急の危険を回避するためのものである等医療上やむを得ない事情があれば、本実施計画書からの逸脱を行うことができる。その際には、研究責任者は、逸脱又は変更の内容及び理由を、可能な限り早急に研究機関の長を経て広島大学再生医療等委員会に報告する。また、研究責任者又は研究分担者は、本実施計画書から逸脱した場合は理由のいかんによらずすべてこれを記録する。

また、本実施計画書の変更・改訂が適切な場合には、その案を、研究機関の長を経て広島大学再生医療等委員会に提出してその承認を得る。変更に際しては変更することの倫理的、科学的、及び医学的妥当性について十分検討する。逸脱・変更に際しての手続きは定められた手順に従う。

再生医療等が再生医療等提供計画に適合していない状態であると知ったときは、速やかにその旨を再生医療等の提供を行う医療機関の管理者に報告する。また、不適合であって、特に重大なものが判明した場合は、速やかに認定再生医療等委員会の意見を聴くこと。

13 臨床研究全体の終了又は中止及び中断

13.1　臨床研究の終了

目標症例の登録が予定期間内に終了した場合、最終登録症例の観察期間終了日を臨床研究実施期間終了日とし、以下の手順をふむ。

13.1.1　臨床研究における研究の終了の手順

研究責任者は、臨床研究実施期間が終了したことを統計解析責任者、広島大学再生医療等委員会、広島大学病院管理者及び関連部門に報告する。

また、研究責任者は、臨床研究終了後速やかに総括報告書を作成し、広島大学病院管理者に提出しなければならない。さらに，広島大学病院管理者は受理した総括報告書の写しを速やかに厚生労働大臣に提出しなければならない。

13.2　臨床研究全体の中断・中止の基準及び手順

13.2.1　臨床研究全体の中断・中止基準

研究責任者は、以下の事項に該当する場合は、臨床研究継続の可否を検討する。

1. NK 細胞調製の品質、安全性、有効性に関する重大な情報が得られた場合

2. 症例登録の遅れ、プロトコル逸脱の頻発などの理由により、臨床研究の完遂が困難と判断された場合
3. 広島大学再生医療等委員会の評価等に基づき、プロトコル治療の安全性に問題があると判定された場合
4. 論文や学会発表など、本臨床研究以外から得られた関連情報を評価した結果、プロトコル治療の安全性に問題があると判断された場合、又は臨床研究継続の意義がなくなったと判断された場合

13.2.2 臨床研究全体の中断・中止の手順

研究責任者は、臨床研究中止後速やかに広島大学再生医療等委員会に報告する。

また、研究責任者は臨床研究中止後速やかに総括報告書を作成し、広島大学病院管理者に提出しなければならない。さらに、広島大学病院管理者は受理した総括報告書の写しを速やかに厚生労働大臣に提出しなければならない。

14 症例報告書

14.1 データマネージメント

データマネージメントについては、広島大学病院　総合医療研究推進センターの臨床研究品質管理（モニタリング、データマネージメント）に関する標準業務手順書に準じて、同センターのデータサイエンス部門（以下、データセンター）に委託する。

14.2 症例報告書の作成とデータの収集

1. 研究責任者又は分担者は、登録した被験者について症例報告書を作成し、記名捺印又は署名の上、データセンターに提出し、その写しを保存する。
2. 研究協力者が症例報告書の作成補助を行う場合には、研究責任者又は研究分担者の監督のもと、医学的判断を伴わない範囲での原資料からの転記にとどめる。

14.3 症例報告書の記載上の注意

1. 黒色のボールペン又は黒インクのペンで記載する。
2. □は該当するものにレ印又は×印を記載する。
3. 観察・検査未実施でデータがない場合には、記載欄に斜線（/）を入れる。
4. 原資料との整合性を確認する。

14.4　症例報告書の変更又は修正

1. 症例報告書の変更又は修正の際には、変更又は修正箇所を二重線（=）で消し、変更又は修正箇所の近隣に正しい内容を記載し、変更又は修正日を併記の上、捺印又は署名する。当初の記載内容を不明瞭にしないよう修正液、砂消しゴム等は使用しない。
2. 重要事項［同意、エンドポイントの評価（有害事象名、発現日、Grade、重篤度、処置、転帰/判定日、因果関係、コメント）］に関する変更又は修正では、変更又は修正日に加えて変更又は修正の理由を記載し、捺印又は署名する。
3. データセンターへ提出後の症例報告書の変更又は修正は、データセンターが指定する DCF（Data Clarification Form）を介して行う。

14.5　症例報告書の確認

1. 研究分担者が症例報告書を作成した場合には、研究責任者は、症例報告書をデータセンターに提出する前に、その記載内容を点検し、問題がないことを確認した上で記名捺印又は署名し、データセンターに提出する。
2. 研究責任者は、データセンターに提出する症例報告書の記載内容が正確かつ完全で読みやすく、提出時期が適切であること、及び被験者の識別に被験者識別コード及び登録番号を用いていることを保証する。

14.6　症例報告書の提出

研究責任者、研究分担者、研究協力者は、当該症例における各分冊の最終観察終了後又は臨床研究中止後 6 週以内に症例報告書を作成し、記名捺印又は署名した上でデータセンターへ提出し、その写しを保存する。

15 統計解析

15.1　統計解析方法

原則として、連続値で観察される項目は、要約統計量として例数、平均値（中央値）、標準偏差（四分位点）、範囲（最小値－最大値）を算出する。また、離散値で観察される項目は、要約統計量として各カテゴリの例数とその割合を算出する。

主要解析の統計学的検定は有意水準（両側）5%で実施する。

15.2　解析対象集団の定義

15.2.1　有効性解析対象集団

本臨床研究に登録され、かつ生体部分肝移植手術が実施され、術後 3 日目に NK 細胞が投与された被験者を有効性解析対象集団（最大の解析対象集団　Full Analysis Set：FAS）とする。

15.2.2　安全性解析対象集団

本臨床研究に登録され、かつ生体部分肝移植手術が実施され、術後 3 日目に NK 細胞が投与された被験者を安全性解析対象集団とする。

15.3　欠測値の取扱い

試験途中での中止や逸脱による脱落については、

- ✓ 当該被験者の中止・脱落直前までに観察されたデータを最終観察データとして解析を行う（Last Observation Carried Forward：LOCF）
- ✓ 欠測データの補完を行わず、観測値に基づき解析を行う（Observed Case : OC）
- ✓ 中止・脱落例を治療失敗例（菌血症発症例/ Failure）として解析を行う

の方法による解析結果の頑健性について確認する。なお、主要評価項目については治療失敗例（Failure）として解析する方法を主解析とする。

15.4　被験者背景及びベースラインの特性

安全性解析対象集団及び有効性解析対象集団それぞれについて、被験者背景およびベースライン特性を要約統計量を用いて要約し、一覧表に示す。なお、ベースラインとして用いるデータは術前のデータであり、データが複数存在する場合には、手術日に直近のデータを用いることとする。

また、本臨床研究を完遂した被験者数とその割合、中止した被験者数を中止時期毎に要約するとともに中止に至った理由についても要約する。

15.5　有効性の解析

15.5.1　主要評価項目

生体肝移植後 1 カ月以内の菌血症発症率

［主要解析］

FAS を対象とし、生体肝移植後 1 カ月以内の菌血症発症率及びその 95%信頼区間を算出する。また、先行研究において観測された非 NK 療法での菌血症発症率 30%を閾値として以下の統計学的仮説検定をカイ二乗検定により有意水準（両側）5%で行う。
帰無仮説「NK 療法における菌血症発症率 p が、閾値 30%と等しい（$p = 0.3$）」
対立仮説「NK 療法における菌血症発症率 p が、閾値 30%よりも少ない（$p < 0.3$）」

15.5.2 副次評価項目

① 全生存期間：
FAS を対象に、手術日を起点として死亡日（死亡原因を問わない）又は生存を確認した日までの期間を観察し、カプランマイヤー法を用いて全生存期間の生存曲線を推定し、全生存期間の中央値とその 95%信頼区間を算出する。また、6 カ月、1 年及び 3 年後の生存率とその 95%信頼区間を計算する。なお、信頼区間の算出には、Greenwood 法を用いる。

② 免疫応答への影響（CFSE-MLR によるドナー特異的免疫応答性の評価、DSA の検出、拒絶反応発症率、レシピエント末梢血 NK 細胞活性評価）：
FAS を対象に CFSE-MLR 及びレシピエント末梢血 NK 細胞活性評価については、各観測時点の要約統計量及びベースラインからの変化量（平均値（中央値）、標準偏差（四分位点））とその 95%信頼区間を算出する。また、DSA の検出、拒絶反応発症率については各カテゴリの例数とその割合を算出する。

③ 肝細胞癌再発／De novo 発癌
FAS を対象に、肝細胞癌の再発の有無及び再発日を観察し、肝細胞癌再発割合とその 95%信頼区間及び再発までの平均期間とその 95%信頼区間を算出する。なお、De novo 発癌についても同様に解析を行う。

④ 感染症発症率
細菌感染症、サイトメガロウイルス感染症、真菌感染症が発症した被験者の割合とその 95%信頼区間を算出する。また、遺伝子多型別の感染症発症率を算出し、遺伝子多型間の比較を Fisher の直接確率法により有意水準（両側）5%で比較する。

15.6 安全性の解析

有害事象

有害事象の事象別、及び重症度別に発現例数、発現件数及び発現割合を集計する。またプロトコル治療との因果関係が否定できない有害事象についても同様な集計を行う。

バイタルサイン

術後の予定された測定時点の観測値と術前検査での観測値（ベースライン）から各時点の変化量に対してバイタルサインの要約統計量を求め、一覧表に示す。

臨床検査値

術後の予定された測定時点の観測値と術前検査での観測値（ベースライン）から各時点の変化量に対して臨床検査値（血液学的検査、生化学的検査）の要約統計量を求め、一覧表に示す。なお、定性的臨床検査データについては、ベースラインと各測定時点の検査カテゴリ値のペアの頻度を分割表の形式で集計する。

15.7　解析計画の変更手順

試験開始後に解析計画の変更あるいは追加が生じた場合、その変更あるいは追加の妥当性、及び試験の評価への影響を検討し、解析計画の変更あるいは追加に至った経緯を総括報告書に記載する。主要な解析や重要な評価項目の解析については、実施計画書の変更も併せて行う。

16 臨床研究の品質管理

16.1　品質管理

研究責任者、研究分担者及び研究協力者は、再生医療等の安全性の確保等に関する法律、人を対象とする医学系研究に関する倫理指針及び本実施計画書を遵守して臨床研究を行う。また、研究責任者、研究分担者及び研究協力者は、臨床研究の実施に関わるそれぞれの手順書に従って臨床研究を行う。

16.2　モニタリング

本臨床研究は、侵襲を伴い介入を伴う研究に該当するため、研究責任者は広島大学病院総合医療研究推進センターにモニタリング業務を委託し、当該センターが所有する臨床研究の品質管理に関する標準手順書に準じてモニタリングを実施する。

なお、モニタリング担当者は別途定めた実施時期にモニタリングを行い、再生医療等の安全性の確保等に関する法律、人を対象とする医学系研究に関する倫理指針及び本実施計画書を遵守して適切に実施されていること及びデータの信頼性が十分に確保されていることを確認し、モニタリングの結果を研究責任者に報告する。

16.3 データ管理

研究責任者又は分担者は、被験者から得られたデータを症例報告書に遅滞なく記載し作成する。症例報告書、ドナー肝臓由来活性化リンパ球製造指図書、同意書、検査結果のコピーは症例ごとにファイルし、広島大学大学院医系科学研究科消化器・移植外科のカギがかかる書庫に保存する。なお、作成された症例報告書は遅滞なくデータセンターに提出し、データセンターは広島大学病院総合医療研究推進センターが所有する臨床研究の品質管理に関する標準手順書に準じてデータ管理を行う。

16.4 効果安全性評価委員会

16.4.1 効果安全性評価委員会による審議内容

効果安全性評価委員会は、本研究分担者を除く 3 名の医師とし、以下の事項について審議を行う。

- 1 年に 1 回の実施状況報告の内容
- NK 細胞が投与された 15 例目の手術後 1 カ月の観察期間が終了した時点で行う中間集計での有効性及び安全性の確認と研究継続の可否
- 重篤な有害事象の評価
- その他、研究責任者あるいは効果安全性評価委員会が必要と判断した事項

16.4.2 効果安全性評価委員会による勧告

効果安全性評価委員会により勧告すべき事項が提案された場合には、内容と理由を研究責任者に対して勧告する。

16.4.3 監査

本研究では、監査を実施しない。

16.5 研究者の教育および研修

研究責任者は、本研究に参加する研究者を対象として、研究開始前、および研究開始後は年度ごとに少なくとも 1 回、以下に例示するような事項について理解を深めるための、教育及び研修の場を設ける。

1. 厚生労働省「再生医療等の安全性の確保に関する法律」について
2. 特定細胞加工物等に関する知識（倫理的な考え方を含む）
3. 調製される細胞培養加工物等の安全な取扱いに関する知識及び技術
4. 施設・装置に関する知識及び技術

5. 調製工程の安全性に関する知識及び技術
6. 事故発生時の措置に関する知識及び技術
7. 「人を対象とする医学系研究に関する倫理指針」に準じて、研究倫理並びに研究実施に必要な知識及び技術

17 広島大学再生医療等委員会

17.1　広島大学再生医療等委員会による審議

広島大学再生医療等委員会委員長は、臨床研究の進捗状況報告につき、1 年に 1 回審議を行う。また、広島大学再生医療等委員会員長は、研究責任者から依頼があった場合、もしくは自らが必要と判断した場合にも、審議を行うことができる。審議形式については、審議内容の重要度に応じて、委員会の招集、持ち回り、電話・メール等による意見聴取など委員長が決定する。

17.2　広島大学再生医療等委員会の審議内容

広島大学再生医療等委員会は以下の事項について審議する。

1. 進捗状況確認
2. 重篤な有害事象の発現による研究継続の可否
3. その他委員長が必要と判断した事項

17.3　広島大学再生医療等委員会による勧告

勧告すべき事項が提案された場合、全審議参加委員の合意のもとに、内容と理由を研究責任者に対して勧告する。全委員の合意が得られない場合には、少数意見も併記した上で、委員長が意見を取りまとめ、勧告を行う。

提供機関管理者は、再生医療等委員会から意見を受けて講じた再生医療等提供計画の変更その他の措置について、当該委員会に報告を行わなければならない。

18 臨床研究の倫理的実施

本臨床研究は、ヘルシンキ宣言に基づく倫理的原則に留意し、「再生医療等の安全性の確保等に関する法律」、「人を対象とする医学系研究に関する倫理指針」及び本実施計画書を遵守して実施する。

18.1　広島大学再生医療等委員会

広島大学再生医療等委員会は、広島大学病院管理者の諮問を受け、臨床研究実施計画書、説明文書（患者さん及び肝臓を提供する方へ）、症例報告書の様式の記載内容にもとづき、倫理的、科学的及び医学的妥当性の観点から臨床研究の実施及び継続について審議を行う。

18.2　臨床研究の進捗報告

再生医療等の提供状況について、次に掲げる事項について広島大学再生医療等委員会及び厚生労働大臣に報告しなければならない。

(1)　当該再生医療等を受けた者の数

(2)　当該再生医療等に係る疾病等の発生状況及びその後の経過

(3)　当該再生医療等の安全性及び科学的妥当性についての評価

(4)　当該再生医療等の提供を終了した場合にあっては、終了した日

本報告は、再生医療等提供計画を厚生労働大臣に提出した日から起算して、1 年ごとに、当該期間満了後 90 日以内に行わなければならない。広島大学再生医療等委員会への報告は、別紙様式第 3 による報告書、厚生労働大臣への報告は、別紙様式第 4 による報告書を提出して行う。

18.3　被験者の人権及び個人情報の保護に関する事項

18.3.1　被験者の人権

本研究は、世界医師会による「ヘルシンキ宣言」、「再生医療等の安全性の確保等に関する法律」及び「人を対象とする医学系研究に関する倫理指針」を遵守して行う。研究責任者及び研究分担者は、被験者の人権の保護の観点から被験者の健康状態、症状、年齢、性別、同意能力等を十分考慮し、本臨床研究への参加を求めることの適否については慎重に検討する。また、社会的に弱い立場にある者を被験者とする場合には、特に慎重な配慮を払う。

18.3.2　個人情報の保護

被験者の同意取得後はデータ管理、製造管理など、症例の取り扱いにおいては全て連結可能匿名化された被験者識別コード又は登録番号により管理され、匿名化コードと氏名の対照表及び氏名記載同意書は施錠可能な書類保管庫に厳重に保管する。また、公表に際しては被験者の名前が直接公表されることがない等、被験者の個人情報の保護については十分に配慮する。

個人情報の管理者：

所属　広島大学大学院医系科学研究科成人健康学　職名　教授　氏名　田邊　和照

19 記録等の保存

19.1　試料の保存

臨床研究期間中に採取された細胞等は基礎実験に使用し、その他の研究にも使用する可能性があるため、広島大学大学院医系科学研究科消化器・移植外科学で使い切るまで保存する。保存方法は、試料を符号化して匿名化の上管理するが、予後などとの関連を検討する可能性もあるため、個人識別情報管理者においては、連結可能匿名化の状態で行う。将来、試料を他の研究に用いる際には再度、広島大学再生医療等委員会における倫理審査を受ける。試料の一部及び投与細胞の一部は将来の有害事象原因検索のために10年間凍結保存を行う。試料の廃棄は、研究終了後最低10年間経過した後にオートクレーブで滅菌処理を行った後に感染性医療廃棄物として廃棄する。

19.2　資料の保存

症例報告書を始め臨床研究に係る文書及び記録等の保存は広島大学大学院医系科学研究科消化器・移植外科学で行う。

再生医療を受けた記録は、次に掲げる事項について再生医療等を受けた者ごとに作成する。

(1)　再生医療等を受けた者の住所・氏名・性別・及び生年月日

(2)　病名及び主要症状

(3)　使用した特定細胞加工物または再生医療等製品の種類、投与方法その他の再生医療等の内容及び評価

(4)　再生医療等に用いる細胞に関する情報（当該細胞が採取された場所、年月日、当該細胞提供者の適格性の確認の結果及び当該細胞について適切性を確認した検査の結果等）

(5)　特定細胞加工物の製造を委託した場合は委託先及び委託業務の内容

(6)　再生医療等を行った年月日

(7)　再生医療等を行った医師の氏名

臨床研究に係る文書（再生医療等提供計画、同意に係る文書及び特定細胞加工物概要書）及び記録等は、臨床研究を中止又は終了し総括報告書を提出した日から少なくとも30年間保存する。本実施計画書及び症例報告書は変更・修正があった場合はその履歴を適切に保存する。

記録等の管理責任者：

所属　広島大学大学院医系科学研究科成人健康学　職名　教授　氏名　田邊　和照

20 臨床研究総括報告書の作成

研究責任者は､臨床研究の中止又は終了後､速やかに臨床研究総括報告書を作成する。

21 臨床研究終了後の被験者情報の把握

研究終了後も定期的な外来受診を促す。定期的外来診療により合併症の有無、及び有効性について評価を行う。再生医療等の提供による重篤な有害事象は全て報告を行う。投与終了後 3 年間は外来通院を継続していただく。

なお、臨床研究終了後の定期的外来診療で得られたデータは、解析には含めない。疾病等の発生が生じた場合は広島大学再生医療等委員会に報告を行う。

22 臨床研究費用並びに健康被害の補償

22.1　臨床研究の資金源及び利益相反

研究費（運営費交付金）

本臨床試験は広島大学　消化器･移植外科学に分配される運営費交付金により実施する。なお、利害の衝突に関しては、広島大学の臨床研究利益相反管理委員会で審査を受けており、開示すべき利益相反はない。

22.2　臨床研究に関する費用負担

本臨床研究で､広島大学病院にて登録された症例の臨床研究実施にかかる費用は広島大学大学院医系科学研究科消化器・移植外科学の研究費で負担する。

22.3　健康被害の補償等

再生医療を受ける者 : 本臨床研究に起因する有害事象が発生した場合、研究責任者は医学上最善の処置を取る事により被験者の回復に努める。また、本臨床研究は臨床研究補償保険に加入しており、本研究の実施に起因して、過失によらず死亡または重篤な有害事象等の健康被害が生じた際には､その被害が被験者の責に帰すべき事由により引き起こされた等の免責事由に相当する場合を除いて､再生医療等臨床研究保険によって補償される。

細胞提供者：治療に用いる細胞は、通常の肝臓移植ドナー手術の際に肝臓が体外に摘出されてから清潔に回収される。従って、細胞提供者は本治療参加により健康被害が発生することはない。肝臓移植ドナー手術に関連した健康被害が発生した場合は、医療保険による治療を行う。

23 臨床研究成果の帰属及び臨床研究結果の登録・公表に関するとり決め

本臨床研究により生じる知的財産権は研究者に帰属するものとする。

本臨床研究の実施に先立ち、大学病院医療情報ネットワーク研究（UMIN）センターが設立したデータベースに事前登録する。本臨床研究の結果は、総括報告書としてまとめることとする。また必要に応じて論文又は学会発表として公表する。公表に際しては被験者の名前が直接公表されることがない等、被験者の個人情報の保護については十分に配慮する。

臨床研究実施体制

23.1　研究責任者

氏　名	機関名、部署・所属、役職、電話番号	臨床研究において果たす役割
大段秀樹	広島大学大学院医系科学研究科<br>消化器・移植外科学　教授<br>[redacted]	研究実施責任者<br>患者の選定、手術、評価

23.2　研究分担者

氏　名	機関名、部署・所属、役職、電話番号	臨床研究において果たす役割
大平真裕	広島大学病院　未来医療センター<br>助教<br>[redacted]	患者の選定、手術、評価<br>臨床研究管理者
田中友加	広島大学大学院医系科学研究科<br>消化器・移植外科学　准教授<br>[redacted]	免疫学的評価
田中純子	広島大学大学院医系科学研究科<br>統合健康科学部門<br>疫学・疾病制御学　教授<br>[redacted]	統計解析
小林剛	広島大学大学院医系科学研究科	患者の選定、手術、評価

	消化器外科・移植外科　講師<br>[redacted]	
井手健太郎	広島大学病院　消化器外科・移植外科<br>助教<br>[redacted]	患者の選定、手術、評価
田原裕之	広島大学病院　消化器外科・移植外科<br>助教<br>[redacted]	患者の選定、手術、評価

23.3　連絡先

名称	名称、所属、代表者（役職・氏名）[redacted]
実施医療機関<br>研究責任者	名称：広島大学大学院医系科学研究科<br>所属：消化器・移植外科学<br>代表者：　教授　大段秀樹<br>[redacted]
研究事務局	名称：広島大学大学院医系科学研究科　消化器・移植外科<br>担当者：助教　大平真裕<br>[redacted]
データセンター<br>モニタリング	名称：広島大学病院　総合医療研究推進センター　データサイエンス部門<br>[redacted]
相談窓口	名称：広島大学病院　未来医療センター<br>担当者：大平真裕<br>[redacted]

24 文献

Colonna, J. O., 2nd, Winston, D. J., Brill, J. E., et al. (1988). Infectious complications in liver transplantation. *Arch Surg, 123*(3), 360.

Fishman, J. A., & Rubin, R. H. (1998). Infection in organ-transplant recipients. *N Engl J Med, 338*(24), 1741.

Horan, T. C., Andrus, M., & Dudeck, M. A. (2008). CDC/NHSN surveillance definition of health care-associated infection and criteria for specific types of infections in the acute care setting. *Am J Infect Control, 36*(5), 309.

Ishiyama, K., Ohdan, H., Ohira, M., et al. (2006). Difference in cytotoxicity against hepatocellular carcinoma between liver and periphery natural killer cells in humans. *Hepatology, 43*(2), 362.

Kawecki, D., Chmura, A., Pacholczyk, M., et al. (2009). Bacterial infections in the early period after liver transplantation: etiological agents and their susceptibility. *Med Sci Monit, 15*(12), CR628.

Kim, S. I., Kim, Y. J., Jun, Y. H., et al. (2009). Epidemiology and risk factors for bacteremia in 144 consecutive living-donor liver transplant recipients. *Yonsei Med J, 50*(1), 112.

Ochi, M., Ohdan, H., Mitsuta, H., et al. (2004). Liver NK cells expressing TRAIL are toxic against self hepatocytes in mice. *Hepatology, 39*(5), 1321.

Ohira, M., Ishiyama, K., Tanaka, Y., et al. (2009). Adoptive immunotherapy with liver allograft-derived lymphocytes induces anti-HCV activity after liver transplantation in humans and humanized mice. *J Clin Invest, 119*(11), 3226.

Ohira, M., Nishida, S., Tryphonopoulos, P., et al. (2012). Clinical-scale isolation of interleukin-2-stimulated liver natural killer cells for treatment of liver transplantation with hepatocellular carcinoma. *Cell Transplant, 21*(7), 1397.

Ohira, M., Ohdan, H., Mitsuta, H., et al. (2006). Adoptive transfer of TRAIL-expressing natural killer cells prevents recurrence of hepatocellular carcinoma after partial hepatectomy. *Transplantation, 82*(12), 1712.

Soave, R. (2001). Prophylaxis strategies for solid-organ transplantation. *Clin Infect Dis, 33 Suppl 1*, S26.

Tashiro, H., Ishiyama, K., Ohira, M., et al. (2011). Impact of adjuvant immunotherapy using liver allograft-derived lymphocytes on bacteremia in living-donor liver transplantation. *Transplantation, 92*(5), 575.

Winston, D. J., Emmanouilides, C., & Busuttil, R. W. (1995). Infections in liver transplant recipients. *Clin Infect Dis, 21*(5), 1077.
